# Disparities in the burden of tuberculosis associated with urbanization across 178 countries and territories: an observational study

**DOI:** 10.3389/fpubh.2025.1658814

**Published:** 2025-09-09

**Authors:** Yaping Wang, Qiao Liu, Zhongdan Chen, Min Liu, Bin Chen, Yanlin Zhao, Jue Liu

**Affiliations:** ^1^Department of Epidemiology and Biostatistics, School of Public Health, Peking University, Beijing, China; ^2^Key Laboratory of Epidemiology of Major Diseases (Peking University), Ministry of Education, Beijing, China; ^3^World Health Organization Representative Office for China, Beijing, China; ^4^Institute of Remote Sensing and Geographic Information System, Peking University, Beijing, China; ^5^National Center for Tuberculosis Control and Prevention, Chinese Center for Disease Control and Prevention, Beijing, China; ^6^Institute for Global Health and Development, Peking University, Beijing, China; ^7^Global Center for Infectious Disease and Policy Research & Global Health and Infectious Diseases Group, Peking University, Beijing, China

**Keywords:** urbanization, tuberculosis, multi-drug resistant tuberculosis, economic inequalities, prevention

## Abstract

**Background:**

Tuberculosis (TB) remains the leading cause of death from a single infectious agent. However, there is limited quantitative evidence on the impact of urbanization on TB burden. We aimed to assess the relationship between urbanization and the global TB burden.

**Methods:**

Using multi-source data, we developed a composite index of urbanization across 178 countries and territories from 2012 to 2019, incorporating the proportion of urban population, the proportion of population using improved sanitation, nighttime light intensity, normalized difference vegetation index, and per capita gross domestic product. Fixed-effects linear models were applied to estimate the rate ratios (RRs) and 95% confidence intervals (CIs) for the association between urbanization and the incidence, prevalence, and mortality of total TB and three subtypes: drug-susceptible TB (DS-TB), multidrug-resistant TB (MDR-TB), and extensively drug-resistant TB (XDR-TB).

**Results:**

Overall, higher urbanization scores were associated with significant reductions in the burden of MDR-TB and XDR-TB but showed no effect on total TB or DS-TB. For MDR-TB, each additional urbanization score was associated with a 1.0% decrease in incidence (RR = 0.990; 95% CI: 0.985–0.996), a 1.1% decrease in prevalence (0.989; 0.984–0.994), and a 0.7% decrease in mortality (0.993; 0.988–0.998). For XDR-TB, the corresponding reductions were 0.9% in incidence (0.991; 0.986–0.996), 1.0% in prevalence (0.990; 0.985–0.995), and 0.7% in mortality (0.993; 0.988–0.998). These relationships persisted when considering a one-year lag in urbanization. In subgroup analyses, however, urbanization was associated with increased MDR-TB and XDR-TB burdens in upper-middle-income countries.

**Conclusion:**

Urbanization was linked to reduced MDR-TB and XDR-TB burdens globally, but to increased burdens in upper-middle-income countries. Building well-managed and healthy cities is essential not only for sustainable urbanization but also for strengthening TB prevention and control, especially in rapidly transitioning upper-middle-income countries.

## Introduction

Urbanization is one of the significant demographic shifts of the past century and continues to shape the 21st century. In 2018, an estimated 55% of the world’s population lived in urban areas, a proportion projected to rise to 68% by 2050 (1). This growth is unevenly distributed, with 96% of the increase expected in less developed regions (1). Urbanization is characterized by the expansion of urban populations, socioeconomic transformations, and the fragmentation of ecological systems, all of which have profound implications for human health and well-being. The health effects of urbanization have been most extensively studied in relation to mental health and noncommunicable chronic diseases (NCDs) (2, 3). A systematic review found that the effects of urbanization on depression, the most common mental disorder, were complex (2). Four of seven studies revealed that living in urban areas was associated with elevated odds or more symptoms of depression, whereas the other three studies found that urbanization had protective effects on depression (2). Similarly, some studies have found that the prevalence of type 2 diabetes, physical inactivity, and high body mass index was strongly associated with urban residency (3, 4), whereas one study indicated that urbanization may partially mitigate the adverse influence of population aging (5). Collectively, these findings indicate that urbanization exerts complex and sometimes contradictory effects on both mental and physical health.

Tuberculosis (TB), although preventable and curable, remains the leading cause of death from a single infectious agent, apart from coronavirus disease (COVID-19) (6). According to the World Health Organization (WHO), globally in 2023, the number of people who developed TB was 10.8 million, and TB caused 1.25 million deaths (6). Due to COVID-19-related disruptions in the prevention and control of TB, the global targets for the End TB Strategy have either been missed or remain off track (6). Theoretically, given that TB transmission is shaped by social and ecological environments, urbanization could plausibly influence its dynamics (7). Recently, increasing income inequality in cities may exacerbate risks of TB, as inadequate housing, poor sanitation, and limited access to essential services that fuel TB as a disease of poverty remain barriers for disadvantage (1, 7). A crucial issue is what urbanization could provide to help the world return to the right track or accelerate the process of ending TB. Most studies on the relationship between urbanization and health focused on mental disorders or chronic diseases, rarely on infectious diseases (2–4). For those studies evaluating the effects of urbanization on infectious disease, researchers have concentrated largely on zoonotic and vector-borne diseases that are directly affected by environmental change, rather than airborne diseases such as TB (8, 9). Consequently, robust evidence on how urbanization influences TB burden is still lacking.

To address this gap, we investigated the impact of urbanization on the incidence, prevalence, and mortality of TB, including total TB, drug-susceptible TB (DS-TB), multidrug-resistant TB (MDR-TB), and extensively drug-resistant TB (XDR-TB), across 178 countries (hereinafter referred to as “countries”) for the period 2012–2019 using data from multiple sources.

## Methods

### Data source

We obtained country-level data on TB burden from 1990 to 2019 through the Global Health Data Exchange (GHDx). Data for constructing the urbanization index were sourced from the World Bank (gross domestic product [GDP] per capita, proportion of urban population), Earth Observation Group (nighttime light [NTL]), NASA (normalized difference vegetation index [NDVI]), and our World in Data (proportion of population using improved sanitation). Data on covariates, including demographic characteristics (population density, proportion of adults aged ≥65 years), socioeconomic status (gross national income [GNI] per capita), health services (universal health coverage [UHC] index), meteorological factors (temperature, relative humidity, wind speed), and air pollution (fine particulate matter [PM_2.5_]), were collected from the United Nations, World Bank, WHO, and National Centers for Environmental Information (NCEI).

Considering the available data across countries and to ensure the quality of the data used, we finally included 178 of 204 countries defined by the Global Burden of Disease 2019 (GBD 2019). Since 2012, the NTL map has been produced by the Visible and Infrared Imaging Suite (VIIRS) instead of the Operational Linescan Sensor (OLS) onboard Defense Meteorological Satellite Program (DMSP) satellites, astoundingly improving low-light imaging (10). Therefore, we set the study period from 2012 to 2019. The datasets used in this study are summarized in a diagram illustrating the data preparation procedures (Supplementary Figure S1), and detailed information is described in the following subsections.

### The burden of TB

TB burden, including total TB, DS-TB, MDR-TB, and XDR-TB, was assessed by age-standardized incidence, prevalence, and mortality using data from the GBD 2019. As a systematic scientific study, GBD quantifies the comparative magnitude of health loss due to diseases, injuries, and risk factors by age, sex, and location over time (11). They collected and collated all available data on TB from vital registration systems, surveillance systems, verbal autopsies, prevalence surveys, and population-based tuberculin surveys (12). The data from surveillance and surveys reported to WHO were also utilized to estimate the burden of TB and identify the drug resistance according to the drug-sensitivity results (12). Based on real-world data, the mortality of TB was estimated using the Cause of Death Ensemble modeling (CODEm) strategy, and incidence, prevalence, and cause-specific mortality of TB were modeled by DisMod-MR 2.1, a Bayesian meta-regression modeling tool. The validity of these methods has been demonstrated in previous research (13).

### Urbanization index

Most previous studies have relied on single indicators, such as GDP, the proportion of urban or rural populations, or population density, to assess urbanization, focusing solely on the scale of cities (8, 14). To provide a more comprehensive measure of the urban environment, we developed a composite index using five indicators: the proportion of urban population indicating population migration, the proportion of population using improved sanitation representing healthcare development, NTL presenting the urban construction, NDVI indicating the environmental change, and per capita GDP for economic development.

In comparison to prior research, we additionally added NDVI and NTL as indicators. The NDVI was assessed using remote sensing-based spectral indices that identify vegetation using reflectance measurements and was generated every 16 days at spatial resolutions of 250, 500, or 1,000 meters (15), which measures the relative abundance and spatial distribution of vegetation. NDVI has been widely used to define greenness exposure. We obtained a monthly product (MOD13A3, version 6.1) of NDVI at a spatial resolution of 1 km × 1 km. We then averaged the gridded maps of NDVI into monthly country-level values and finally calculated the annual average NDVI for each country. NTL can provide mixed information on urbanization, including details regarding traffic road networks, city scale, and population distribution (16). We obtained an annual product of global NTL (VIIRS Nighttime Lights, version 2) that uses a reference grid of 15-arc second grid (~500 m at the Equator). We divided it into annual country-level values using the “terra” package in R software.

The composite urbanization score for each country was calculated as the weighted sum of these five indicators. We applied the entropy evaluation method, an objective measure that assigns weights to multiple indicators based on their contribution to the overall system, to determine the weights of each indicator in each year (see Supplementary Table S1 for detailed steps) (17). The average weights for five indicators across 2012–2019 were 0.25 (GDP per capita), 0.62 (NTL), 0.06 (NDVI), 0.02 (sanitation), and 0.05 (proportion of urban population).

### Covariates

Based on current research, we included the following covariates: the proportion of older adults, GNI per capita, population density, UHC index, PM_2.5_, temperature, relative humidity, and wind speed. The proportion of older adults was defined as the percentage of the population aged 65 years and above of the total population. The UHC index measures the coverage of essential health services, typically defined as the average coverage of essential services including reproductive, maternal, newborn, and child health, infectious diseases, non-communicable diseases, and service capacity and access, among the general and most disadvantaged populations (18). All daily mean meteorological factors (temperature, dew point temperature, and wind speed) were derived from the synoptic/hourly observations contained in USAF DATSAV3 Surface data and Federal Climate Complex Integrated Surface Data by NCEI. We first estimated daily relative humidity based on temperature and dew point temperature (19). Then, we calculated the annual average temperature, relative humidity, and wind speed for each monitoring station and finally calculated the average values of all stations at the country level. As a common measure of air pollution, the mean annual concentration of PM_2.5_ was calculated by averaging values from all monitors and was population-weighted in the country.

### Statistical analyses

We used median (IQR) to describe the distribution of single indicators and the composite index of urbanization. The estimated annual percentage change (EAPC) was employed to reflect the change in urbanization level and TB across 178 countries from 2012 to 2019. EAPC is an effective and widely used measure to observe the temporal trends of a specific indicator (13). To calculate the EAPC a regression line was fitted (Equation 1):

(1) y=α+βx+ε

Where y is the natural logarithm form of urbanization score or age-standardized rate of TB; x is calendar year; ε is an independent error term in accordance with a normal distribution. Then, EAPC was calculated as $100\times (e^{\beta }-1)$ , with a 95% confidence interval (CI) derived from the estimation of β. If EAPC and its lower CI boundary are positive (negative), the trends of urbanization and TB are defined as upward (downward) trends. We next used linear regressions to evaluate two sets of relationships: 1) associations between TB, urbanization score and income level (indicated by GNI per capita) in 2019; and 2) associations between the EAPC of TB, the EAPC of urbanization score and income level in 2019.

Two-way fixed-effects models are widely used for causal inference in econometrics and other social sciences and have recently been applied in environmental studies because fixed-effects models can control for all entity confounders that vary across individuals but not over time, as well as all temporal confounders that vary by time but not individuals (20, 21). The specific two-way fixed-effects models (Equation 2) employed in our study to explore the relationship between urbanization and the burden of TB were as follows:

(2) $$\begin{array}{c} \log(Y_{\mathrm{it}})=\beta_{i}+\beta_{t}+\beta_{1}{\text{score}}_{\mathrm{it}}+\beta_{2}\log_{10}{\left(\mathrm{GNI}\right)}_{\mathrm{it}}\\ +\beta_{3}{\text{density}}_{\mathrm{it}}+\beta_{4}{\mathrm{old}}_{\mathrm{it}}+\beta_{5}{\mathrm{UHC}}_{\mathrm{it}}\\ +\beta_{6}{\mathrm{PM}}_{\mathrm{it}}+\beta_{7}{\mathrm{TP}}_{\mathrm{it}}+\beta_{8}{\mathrm{RH}}_{\mathrm{it}}+\beta_{9}{\mathrm{WS}}_{\mathrm{it}}+\varepsilon_{\mathrm{it}} \end{array}$$

Where *i* signifies the number of countries; *i* = 1, 2, …, *N* = 178; *t* indicates time in the analysis, *t* = 2012, 2013, …, *T* = 2019; $Y_{\mathrm{it}}$ represents the incidence, prevalence, or mortality of TB, and is presented in natural logarithm format in the model since these values are highly skewed. $\beta_{i}$ and $\beta_{t}$ construct the intercept in the model. $\beta_{i}$ varies among countries but remains the same over time, while $\beta_{t}$ is country-invariant but varies between years. $\beta_{1}$ and $\beta_{9}$ imply that all countries have the same slope over time. ${\text{score}}_{\mathrm{it}}$ represents the urbanization score, and $\log_{10}{\left(\mathrm{GNI}\right)}_{\mathrm{it}}$ represents the log format of the GNI. Other adjusted confounders are “density” (population density), “old” (proportion of older adults), “UHC” (UHC index), “PM” (mean concentration of PM_2.5_), “TP” (mean temperature), “RH” (mean relative humidity), and “WS” (mean wind speed). $\varepsilon_{\mathrm{it}}$ is the error term. Before analyzing the data using fixed-effects models, we constructed generalized linear models for each year to calculate the variance inflation factors and finally found no multicollinearity among the variables.

Furthermore, to investigate the air pollution and economic inequality in urbanization-attributed impact of TB, we developed subgroup analyses by dividing 178 countries and territories according to WHO guidelines on concentration level of PM_2.5_ (<10 μg/m^3^, 10–14 μg/m^3^, 15–24 μg/m^3^, 25–34 μg/m^3^, ≥35 μg/m^3^), WHO region (Africa, Americas, Eastern Mediterranean, Europe, Southeast Asia, Western Pacific) and income group (high, upper-middle, lower-middle, low), respectively. To examine the robustness of our results, we conducted three sensitivity analyses: (1) using a lower 95% uncertainty interval (UI) of TB burden estimated by GBD; (2) using an upper 95% UI of TB burden estimated by GBD; (3) using the socioeconomic development index to replace the UHC index in the same models.

All statistical analyses were performed in the R program (Version 4.3.0), and data visualization was conducted in Origin 2020b (OriginLab). Two-sided *p* values less than 0.05 were considered statistically significant.

## Results

### Urbanization level

**Figure 1:**
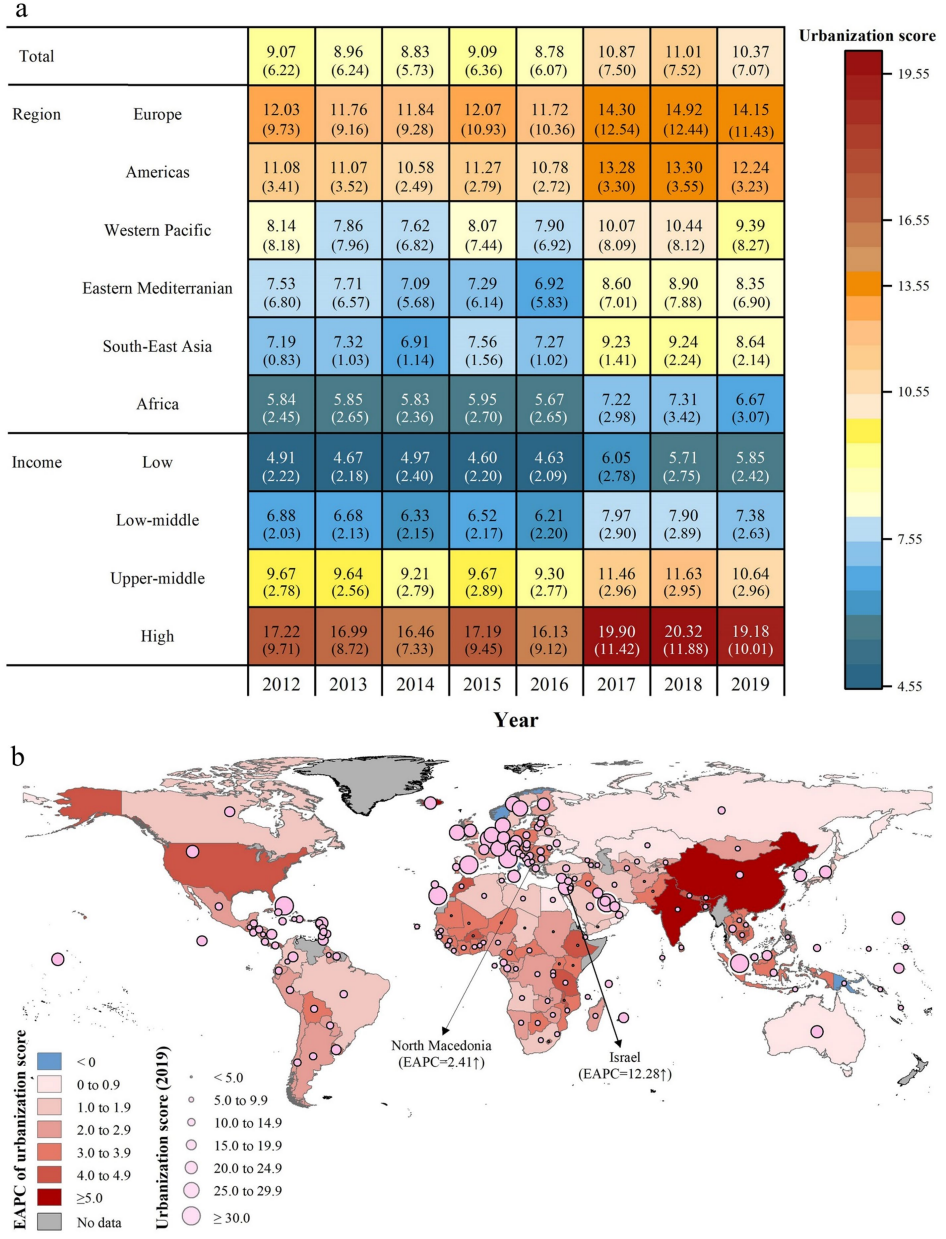
Trends of urbanization levels among 178 countries from 2012 to 2019. **(a)** The median (IQR) urbanization score of 178 countries and territories by WHO region and income level from 2012 to 2019; **(b)** Estimated annual percentage change (EAPC, %) of urbanization levels in 178 countries from 2012 to 2019.

Supplementary Table S2 presents the annual distribution characteristics of the indicators used to construct the urbanization index. Across 178 countries between 2012 and 2019, the urbanization score ranged from 0.72 (Niger in 2016) to 80.29 (Italy in 2016; Supplementary Table S3). Globally, the urbanization score increased from 9.07 (IQR: 6.22) in 2012 to 10.37 (7.06) in 2019 (Figure 1a). Of the 178 countries, 82 (46.1%) exhibited a significant upward trend in urbanization, while the remaining countries showed no prominent change. Rapid urbanization was most evident in Africa and Asia, whereas Europe and North America, where urbanization was already high, exhibited only slight increases (Figure 1b). Specifically, Israel had the highest rate of urbanization, with an annual increase of 12.28% (EAPC = 12.28, 95% CI: 7.00–17.81), while North Macedonia had the slowest rate of urbanization (EAPC = 2.41, 95% CI: 0.04–4.84).

### The global burden of TB

In 2019, the Central African Republic (506.25 per 100,000 population), Burundi (482.36 per 100,000 population), and Lesotho (427.82 per 100,000 population) were the top three countries with the highest incidence of total TB. From 2012 to 2019, the incidence of total TB declined in 158 countries and territories (Supplementary Figure S2a; Supplementary Table S4), with the most rapid fall in Kazakhstan (EAPC = −5.80, 95% CI: −7.11 to −4.47). The Philippines (EAPC = 5.12, 95% CI: 3.06–7.22), South Africa (EAPC = 0.54, 95% CI: 0.01 to 1.08), and Timor-Leste (EAPC = 0.35, 95% CI: 0.29–0.42) were the only three countries with a significant uptrend in total TB incidence from 2012 to 2019. There were 169 and 161 countries with downward trends in prevalence and mortality, respectively (Supplementary Figures S2b,c; Supplementary Tables S5–S6). A similar prominent declining trend was also observed in over 100 countries for the burden of DS-TB and MDR-TB (Supplementary Figures S3, S4; Supplementary Table S7). However, from 2012 to 2019, the incidence, prevalence, and mortality of XDR-TB were significantly aggravated in 124, 115, and 96 countries, respectively (Supplementary Figure S5; Supplementary Table S7).

### Economic inequality in the change of urbanization level and TB burden

**Figure 2:**
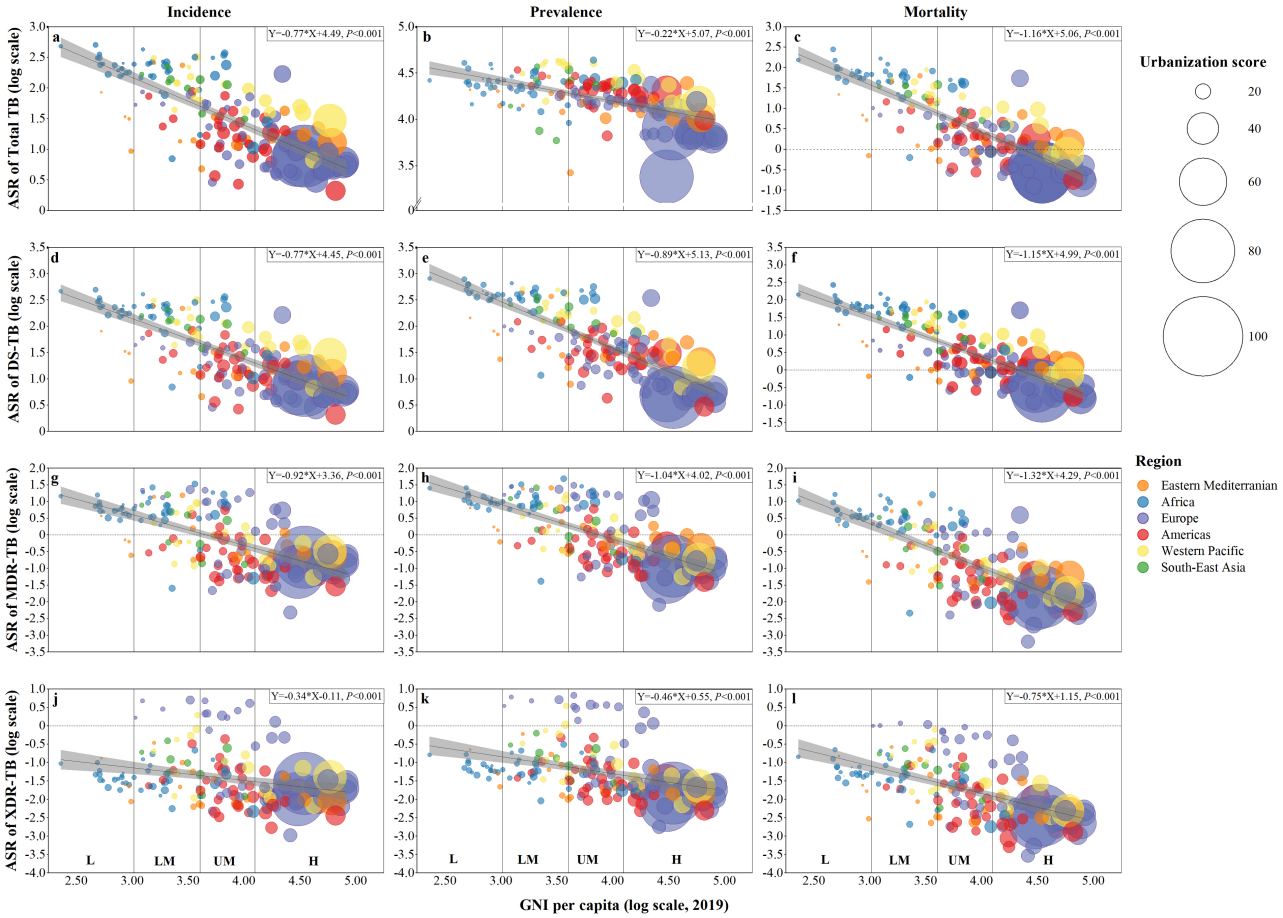
Association of income levels with urbanization score and the burden of tuberculosis in 2019. **(a)** incidence of total tuberculosis; **(b)** prevalence of total tuberculosis; **(c)** mortality of total tuberculosis; **(d)** incidence of drug-susceptible tuberculosis; **(e)** prevalence of drug-susceptible tuberculosis; **(f)** mortality of drug-susceptible tuberculosis; **(g)** incidence of multidrug-resistant tuberculosis; **(h)** prevalence of multidrug-resistant tuberculosis; **(i)** mortality of multidrug-resistant tuberculosis; **(j)** incidence of extensively drug-resistant tuberculosis; **(k)** prevalence of extensively drug-resistant tuberculosis; **(l)** mortality of extensively drug-resistant tuberculosis. In all figures, income levels are measured by gross national income (GNI) per capita and divided into four income levels: low, lower-middle, upper-middle, and high. Each dot represents a country, and the size of the dot is shown by the urbanization score in 2019. The lines are derived by fitting the burden of tuberculosis and GNI using Pearson correlation analyses, and the gray areas represent the confidence intervals (*p*-value <0.05 is considered statistically significant). ASR, age-standardized rate.

In 2019, the lower urbanization level was predominantly found in Africa as well as in low- and lower-middle-income countries (Figures 1a, 2). The burden of total TB and three subtypes of TB decreased with growing country income (all *P* for Pearson’s correlation <0.001). Notably, the burden of total TB, DS-TB, and MDR-TB was mostly concentrated in Africa and Southeast Asia (Figures 2a–i), while the burden of XDR-TB was more prevalent in Europe and the Western Pacific (Figures 2j–l).

**Figure 3:**
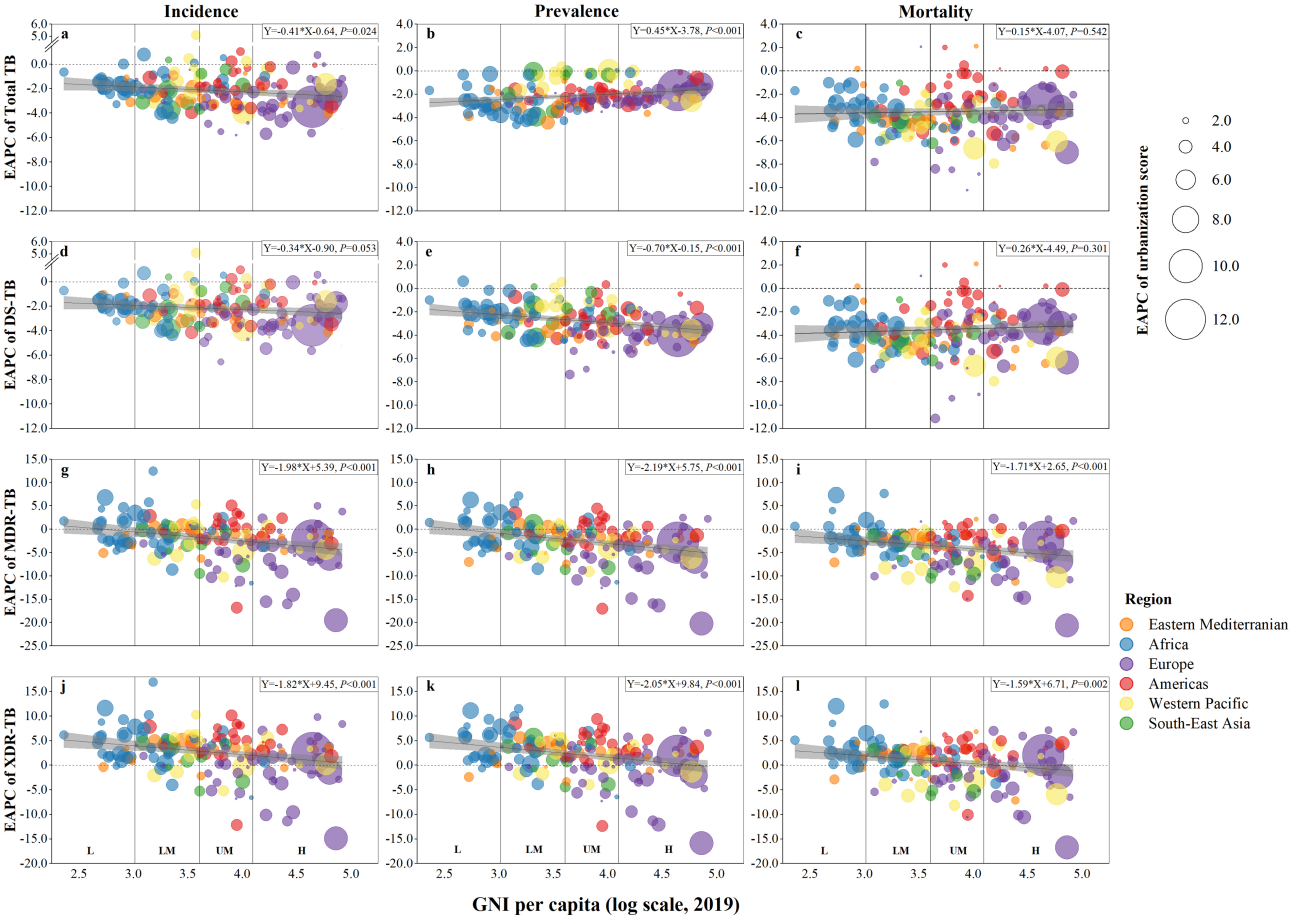
Association of income levels with estimated annual percentage change (EAPC, %) of urbanization score and the burden of tuberculosis from 2012 to 2019. **(a)** incidence of total tuberculosis; **(b)** prevalence of total tuberculosis; **(c)** mortality of total tuberculosis; **(d)** incidence of drug-susceptible tuberculosis; **(e)** prevalence of drug-susceptible tuberculosis; **(f)** mortality of drug-susceptible tuberculosis; **(g)** incidence of multidrug-resistant tuberculosis; **(h)** prevalence of multidrug-resistant tuberculosis; **(i)** mortality of multidrug-resistant tuberculosis; **(j)** incidence of extensively drug-resistant tuberculosis; **(k)** prevalence of extensively drug-resistant tuberculosis; **(l)** mortality of extensively drug-resistant tuberculosis. In all figures, income levels are measured by gross national income (GNI) per capita in 2019 and divided into four income levels: low, lower-middle, upper-middle, and high. Each dot represents a country, and the size of the dot is proportional to the estimated annual percentage change of the urbanization score from 2012 to 2019. The lines are derived by fitting the burden of tuberculosis and GNI using Pearson correlation analyses or binomial models, and the gray areas indicate the confidence intervals (*p*-value <0.05 is considered statistically significant).

Although the most rapid increment of urbanization was in high-income countries (Israel, Ireland, and Iceland), which were located in Europe, the highest average level of increment for urbanization among countries was in Southeast Asia, followed by the Eastern Mediterranean, Africa, and the Western Pacific (Figure 3; Supplementary Table S8). Except for the prevalence and mortality of total TB, and the incidence and mortality of DS-TB, the declining speed of TB was accelerated with increasing income (Figures 3a,b,e,g–l, all *P* for Pearson’s correlation <0.05). Additionally, the decline in the prevalence of total TB was mitigated by increasing income (Figure 3b, *p* <0.001 for Pearson’s correlation).

### Relationship between urbanization and the burden of TB

**Figure 4:**
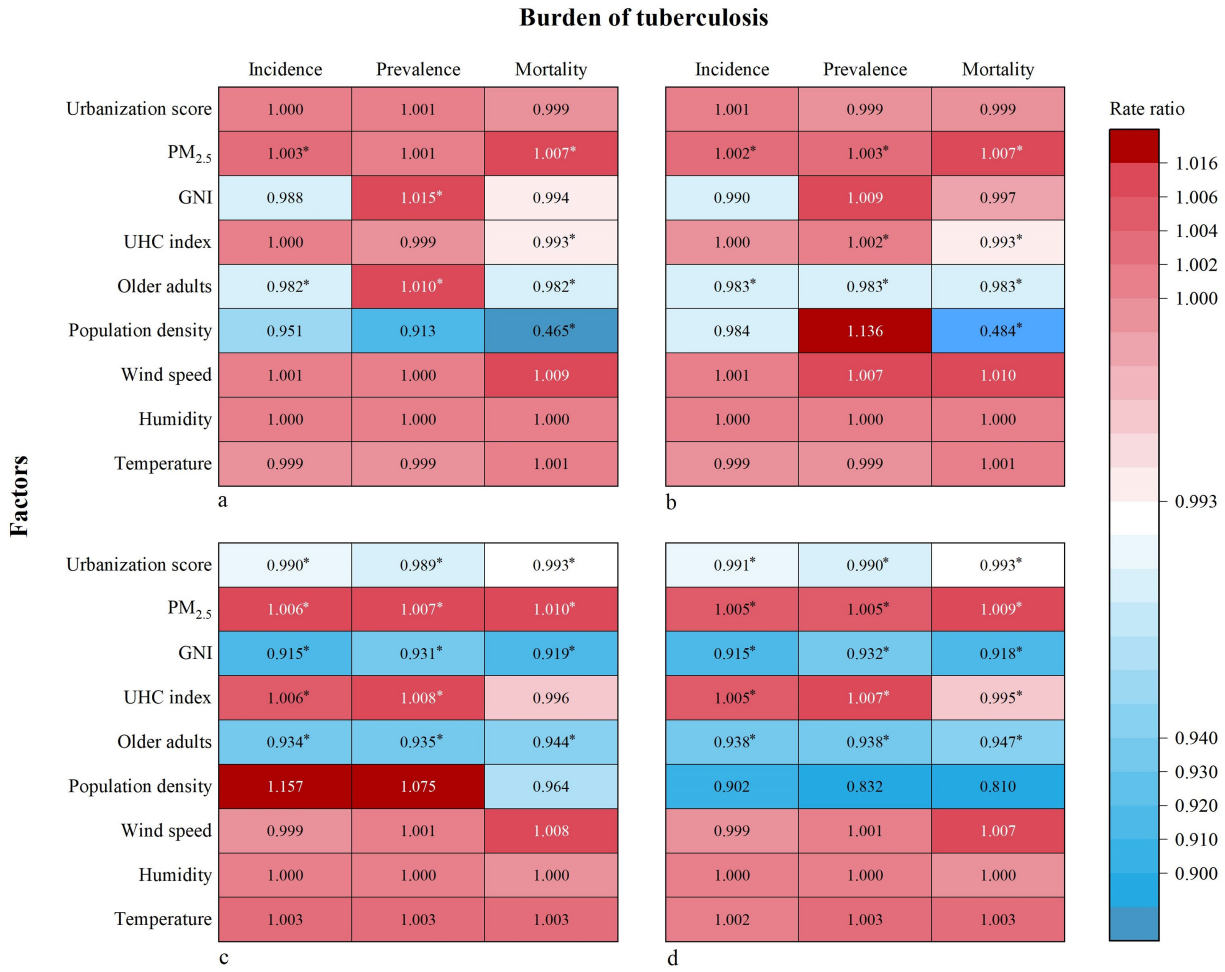
Association between urbanization level and burden of tuberculosis. **(a)** incidence, prevalence, and mortality of total tuberculosis; **(b)** incidence, prevalence, and mortality of drug-susceptible tuberculosis; **(c)** incidence, prevalence, and mortality of multidrug-resistant tuberculosis; **(d)** incidence, prevalence, and mortality of extensively drug-resistant tuberculosis. PM_2.5_, fine particulate matter; GNI, gross national income; UHC, universal health coverage. *p < 0.05.

The urbanization level significantly mitigated the burden of MDR-TB and XDR-TB but had no impact on the burden of total TB and DS-TB (Figure 4; Supplementary Table S9). For MDR-TB, each additional urbanization score was related to a 1.0% decrement in incidence (RR = 0.990, 95% CI: 0.985–0.996), a 1.1% decrement in prevalence (RR = 0.989, 95% CI: 0.984–0.994), and a 0.7% decrement in mortality (RR = 0.993, 95% CI: 0.988–0.998). For XDR-TB, the corresponding reductions with each urbanization score growing were 0.9% in incidence (RR = 0.991, 95% CI: 0.986–0.996), 1.0% in prevalence (RR = 0.990, 95% CI: 0.985–0.995), and 0.7% in mortality (RR = 0.993, 95% CI: 0.988–0.998). Besides the urbanization level, the increment of GNI and the proportion of older adults also alleviated the burden of MDR-TB and XDR-TB (Figure 4). In contrast, higher PM_2.5_ concentrations exacerbated TB burden, particularly the mortality of all subtypes of TB. The main results were robust when we used the lower and upper uncertain intervals of the GBD estimated TB burden or replaced the UHC index with the socioeconomic development index as sensitivity analyses (Supplementary Table S10).

### Lag effects and subgroup analyses

**Figure 5:**
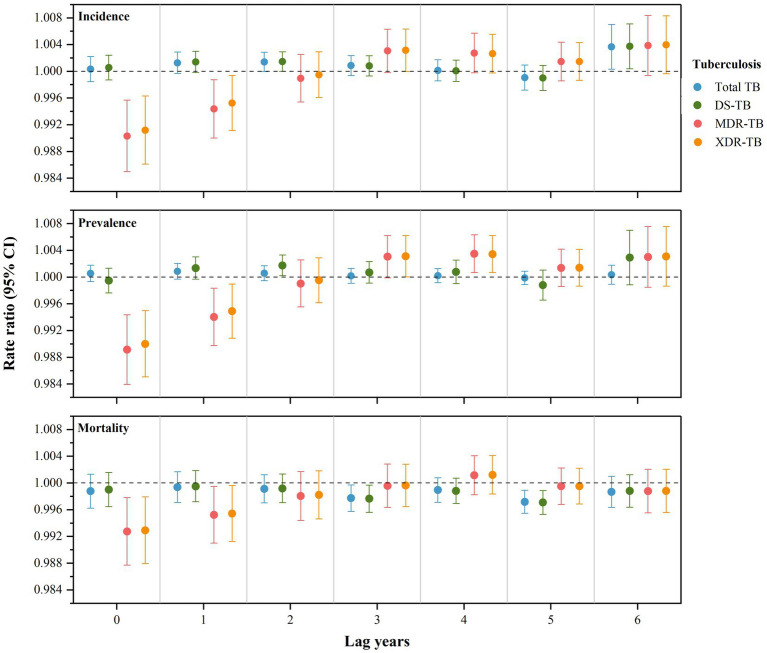
Lag effects of urbanization level on burden of tuberculosis in 178 countries and territories. TB, tuberculosis; DS-TB, drug-susceptible tuberculosis; MDR-TB, multidrug-resistant tuberculosis; XDR-TB, extensively drug-resistant tuberculosis; CI, confidence interval.

Urbanization levels had significant short-term protective lag effects (lag 0 or 1 year) on the burden of MDR-TB and XDR-TB, as well as long-term lag effects (lag 3 or 5 years) on the mortality of total TB and DS-TB (Figure 5; Supplementary Table S11). The protective effect on the burden of MDR-TB and XDR-TB diminished with increasing lag years. For the effects of specific single indicators of urbanization on TB burden, both GDP per capita and NDVI had a positive influence on alleviating the burden of MDR-TB and XDR-TB (Supplementary Figure S6). However, the increase in the proportion of the population using improved sanitation increased the burden of MDR-TB and XDR-TB.

**Figure 6:**
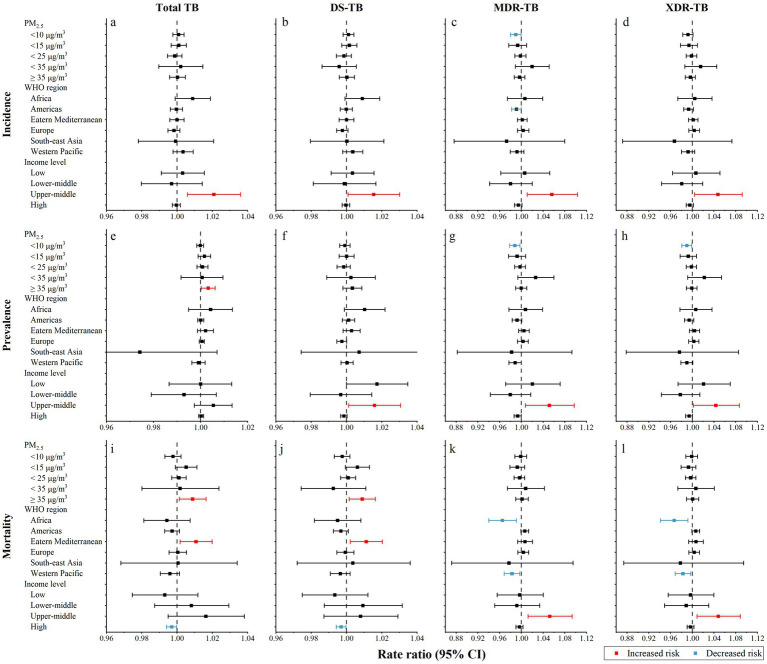
Subgroup analyses on the association between urbanization level and the burden of tuberculosis. **(a)** incidence of total tuberculosis; **(b)** incidence of drug-susceptible tuberculosis; **(c)** incidence of multidrug-resistant tuberculosis; **(d)** incidence of extensively drug-resistant tuberculosis; **(e)** prevalence of total tuberculosis; **(f)** prevalence of drug-susceptible tuberculosis; **(g)** prevalence of multidrug-resistant tuberculosis; **(h)** prevalence of extensively drug-resistant tuberculosis; **(i)** mortality of total tuberculosis; **(j)** mortality of drug-susceptible tuberculosis; **(k)** mortality of multidrug-resistant tuberculosis; **(l)** mortality of extensively drug-resistant tuberculosis. PM_2.5_, fine particulate matter; CI, confidence interval; TB, tuberculosis; DS-TB, drug-susceptible tuberculosis; MDR-TB, multidrug-resistant tuberculosis; XDR-TB, extensively drug-resistant tuberculosis.

In subgroup analyses, we found that positive relationships between urbanization and TB burden, especially MDR-TB and XDR-TB, were pronounced in upper-middle-income countries (UMICs), whereas in high-income countries, we observed negative relationships between urbanization and total TB and DS-TB mortality (Figure 6). Additionally, urbanization decreased the risk of MDR-TB and XDR-TB burden in countries like those in Africa and the Western Pacific. It increased the risk of a total TB and DS-TB burden in countries in the Eastern Mediterranean.

## Discussion

Using data from 178 countries and territories between 2012 and 2019, our study found that all countries with significant changes in urbanization levels were in upward trends, and developing countries had a higher average urbanization increment than developed countries. As national income increased, the decline in TB burden accelerated. We further revealed that urbanization decreased the burden of MDR-TB and XDR-TB, although this association varied depending on the economic context and diminished over longer lag periods. These findings underscore the importance of promoting healthy urbanization as a key measure to achieve the global target of ending TB by 2030.

TB has long been recognized as a disease of poverty. Both across and within countries, individuals with low socioeconomic status are more vulnerable to TB infection due to higher exposure risk (22–24). Undernutrition, closely tied to poverty, weakens the immune system, which is essential for preventing infections and limiting the progression from latent to active TB (22, 23). With urbanization, rural populations often gain access to improved diets with higher protein intake and better nutritional knowledge, which can strengthen immune responses against TB (25–27). Consistent with other studies, we found that as income increases, countries are expected to slow their rate of urbanization. The rapid urbanization rate was particularly high in low-income countries as well as countries in Africa and Asia (1, 24). Some studies have revealed that urban sprawl is driven by population growth (8, 14, 24). According to World Population Prospects, the largest increases in population from 2022 to 2030 and from 2022 to 2050 are in Sub-Saharan Africa, followed by Central and Southern Asia (28). Moreover, with the shift of economic production from agriculture to industry in most developing countries, the extent of investment in city construction has accelerated more than in developed countries. Therefore, this might be the reason that the urban rate was higher in Africa and Asia than in other regions.

We found that urbanization contributed to the decline in the burden of MDR-TB and XDR-TB, and this relationship was pronounced in countries in Africa and the Western Pacific. GDP per capita and NDVI were key contributors to this association. As discussed above, with income growth, the urban population can access improved facilities, safe food, adequate nutrition, and easy access to health services, which protect them from TB infection. Green spaces, as reflected by higher NDVI, improve air quality, reduce noise and extreme temperatures, and encourage physical activity, all of which are linked to lower TB incidence and mortality (29). The significant protective effects of urbanization on the burden of drug-resistant TB (DR-TB), not DS-TB, could result from the relatively higher benefits provided by improved sanitation, NDVI, access to and quality of health services, compared to adverse effects of increased population density and an aggravating environment (30).

In contrast, we found that the urbanization process increased the incidence of all types of TB as well as the prevalence and mortality of MDR-TB and XDR-TB in UMICs. It indicated that the effects of urbanization on TB present a dual impact. The adverse effects of urbanization on TB in UMICs can be attributed to its impact on facilitating pathogen transmission. One explanation is that rapid and dense urban growth in UMICs facilitates the transmission of TB through shared airspaces (31). Unlike high-income countries, where economic growth has paralleled urban expansion, UMICs often experience urban slums and overstretched health systems (32, 33). Although UMICs are expected to improve UHC coverage to 72.4% by 2030, this will remain lower than in high-income countries (34, 35). In addition, the disease patterns in UMICs have been changing from communicable, maternal, neonatal, and nutritional diseases to non-communicable diseases. Compared to high-income countries, health services in UMICs are still unable to adequately address the adverse effects of the aging population and the coexistence of chronic diseases and TB due to the rapid rate of urban growth (36, 37). It was reported that the leading 10 level three causes of disability-adjusted life years (DALYs) in high socio-demographic index countries were non-communicable diseases, except for COVID-19 and injuries in 2021 (38). And over half of people in UMICs were considered at high risk of diabetes, relatively higher than low-income (20.6%) and lower-middle-income (38.0%) countries (39). These chronic diseases, especially diabetes, could increase the risk of tuberculosis and might mitigate the advantages of a higher UHC index of UMICs than low-income countries. Therefore, constructing a well-planned urbanization process in accordance with existing resources and population growth is a pending issue for governments in UMICs.

Another key driver of rising DR-TB risk in UMICs is the transmission of resistant strains and widespread antibiotic misuse. It was demonstrated that, early in the DR-TB epidemic, the acquisition of drug resistance because of inadequate therapy of DS-TB was the cause for the increase in DR-TB cases (40). However, currently, most DR-TB cases are due to the person-to-person transmission of drug-resistant strains (40). Dynamic population flow has increased the potential transmission of drug-resistant strains across the world, and crowded populations in urban areas have increased the possibility of transmission. Another factor, antibiotic overuse and misuse, stimulated the rapid emergence of DR-TB strains and genes (41). Based on a modeling study and a systematic analysis, most UMICs had higher total antimicrobial resistance abundance and lower antimicrobial resistance governance scores (42, 43). Therefore, governments in UMICs need to pay more attention to the protection and improvement of the urban environment, the integrated management of infectious diseases and chronic diseases, and the quality of primary health services. Another potential interpretation might be the limitation of urbanization measurement in our study; some indicators may not accurately represent the unusual situation during the urbanization process. For example, countries with informal economies may have a higher level of NTL, resulting in higher urbanization rates but more possibilities for TB transmission.

We also found an increased risk of total TB and DS-TB related to urbanization in countries in the Eastern Mediterranean. One potential interpretation is the complex disease pattern in this region: some countries still struggle to control infectious diseases, while others face a greater threat to chronic diseases (44). For example, over half of adults were overweight in about 70% of countries, and more than one in four adults were obese in about 60% of countries in this region (45). The prevalence of human immunodeficiency virus (HIV) infection is low in the Eastern Mediterranean region; however, it continues to grow due to the late diagnoses and suboptimal regimens provided by the weak health systems, increasing the risk of TB (45, 46). The demographic distribution in this region is distinctive. Residence in urban settlements is the dominant pattern in this region, with approximately 7 out of 10 people living in cities, resulting in high rates but low-quality urbanization (45). War, conflict, and social or political unrest have led to the forced displacement and migration of millions of people and the rapid influx of refugees into some countries, which could result in crowded cities, strain existing fragile health systems and limited resources, and provide chances of TB transformation (44, 47). Furthermore, economic development in most Eastern Mediterranean countries relied on industrial production, which could produce PM_2.5_ besides vast deserts. As we know, PM_2.5_ is linked to increased risk of TB due to its effects on the human immune system and its role as a carrier through which bacteria enter the body (48). Therefore, with the high urbanization rate attributed to migrants into urban areas, people in the Eastern Mediterranean would have an increased risk of TB.

Based on the mixed effects of urbanization on TB and the Sustainable Development Goals related to urbanization, governments should make greater efforts in the future to build well-planned, managed, and financially sustainable cities. The key components priority to be anchored might include: (1) improved UHC and measures for communicable diseases, including robust primary care systems and TB-specific services to ensure early diagnosis and treatment adherence, particularly for marginalized populations, such as migrants and refugees; (2) rational land use, entailing determination of where, what size, and how to combine land to provide adequate residence for people, for green space and for essential infrastructure; and (3) effective approaches to financing urbanization and economic development, such as establishing urban service funds at the national level, supporting the development of appropriate and affordable housing finance products, and developing vertical and horizontal models of financial resources to decrease inequalities between urban and rural areas (1). Compact urban development generates immense economic, social, environmental, and other intangible benefits that can significantly improve the quality of life for all, which provides potential benefits for the prevention and control of TB. For countries at the upper-middle-income level, as they undergo urbanization, more attention should be given to concerns such as green recovery, economic equality, environmental protection, urban population density, and primary health care to end TB, especially DR-TB. Additionally, during the process of urbanization, governments should also make greater efforts to improve infrastructure (e.g., water and sanitation), rationally use antibiotics, and strengthen effective measures for managing age- and TB-related medical care to meet the needs of the rapidly growing urban population.

Although our study first constructed a complex indicator to evaluate the urbanization level and explore the complex relationship between urbanization and the burden of TB, there were still some limitations in our study. First, although GBD utilized abundant and authoritative data to perform a sophisticated estimation of TB incidence, prevalence, and mortality, it still fell short of the reality of some countries without notifiable data. Second, our study only provided a macroscopic relationship between urbanization and TB. Hence, the mechanisms and specific causes of this relationship require further study, especially those at the sub-country level. Third, although we developed an indicator based on five variables to assess urbanization levels, there remains a need for a more comprehensive indicator to encompass the various domains of urbanization because of its complex definitions. Moreover, our urbanization index cannot capture the qualitative aspects of urban development, such as governance quality, social equity, or effective planning and management.

## Conclusion

Considering the disparities in the burden of tuberculosis associated with urbanization levels, we advocate that governments should, as early as possible, build well-managed and healthy cities with both appropriate population density and habitable environments, not only to support sustainable urbanization but also help the world prevent and control TB. This could be a cost-effective measure for both developing urbanization and ending TB.

## Data Availability

The original contributions presented in the study are included in the article/Supplementary material, further inquiries can be directed to the corresponding author.
