# Supplementary material for: Disparities in the burden of tuberculosis associated with urbanization across 178 countries and territories: an observational study
Source: Front Public Health. 2025 Sep 9;13:1658814. doi: 10.3389/fpubh.2025.1658814 (PMC12454390; doi:10.3389/fpubh.2025.1658814)
Supplement: Supplementary file 1 [file Table_1.DOCX]

Supplementary materials

**Disparities in the burden of tuberculosis associated with urbanization in 178 countries and territories: an observational study**

**Contents**

[Table S1: Steps of Entropy evaluation method for defining the weights of 5 indicators 1](#_Toc205923289)

[Table S2: Characteristics of single urbanization indicators 2](#_Toc205923290)

[Table S3: Urbanization score in 178 countries and territories from 2012 to 2019 3](#_Toc205923291)

[Table S4: Estimated annual percentage change (EAPC, %) for incidence of TB from 2012 to 2019 8](#_Toc205923292)

[Table S5: Estimated annual percentage change (EAPC, %) for prevalence of TB from 2012 to 2019 17](#_Toc205923293)

[Table S6: Estimated annual percentage change (EAPC, %) for mortality of TB from 2012 to 2019 26](#_Toc205923294)

[Table S7: The number of countries (n, %) and the median EAPC (%) with significant change of incidence, prevalence and mortality of tuberculosis from 2012 to 2019 35](#_Toc205923295)

[Table S8: The number of countries (n, %) and the median EAPC (%) with significant change of urbanization level from 2012 to 2019 37](#_Toc205923296)

[Table S9: Results of the fixed-effects models on influence factors associated with burden of tuberculosis 38](#_Toc205923297)

[Table S10: Results of sensitivity analyses on the association between urbanization level and burden of tuberculosis 39](#_Toc205923298)

[Table S11: Lag effects of urbanization level on burden of tuberculosis in 178 countries and territories 40](#_Toc205923299)

[Figure S1: Diagram of data preparation and analysis procedure 41](#_Toc205923300)

[Figure S2: Estimated annual percentage change (EAPC, %) for incidence, prevalence, and mortality of total tuberculosis from 2012 to 2019 43](#_Toc205923301)

[Figure S3: Estimated annual percentage change (EAPC, %) for burden of drug-susceptible tuberculosis from 2012 to 2019 44](#_Toc205923302)

[Figure S4: Estimated annual percentage change (EAPC, %) for burden of multidrug-resistant tuberculosis from 2012 to 2019 45](#_Toc205923303)

[Figure S5: Estimated annual percentage change (EAPC, %) for incidence, prevalence, and mortality of extensively drug-resistant tuberculosis from 2012 to 2019 46](#_Toc205923304)

[Figure S6: Association between single indicators of urbanization and the burden of tuberculosis 47](#_Toc205923305)

# Table S1: Steps of Entropy evaluation method for defining the weights of 5 indicators

| **Step** | **Function** | **Description** |
| --- | --- | --- |
| Step 1 | $X_{ij}^{'}=\frac{X_{ij}-min(X_{j})}{\max\left( X_{j} \right)- min(X_{j})}$ | $X_{ij}$ is the original value of the *j*-th indicator of the *i*-th study county, $X_{ij}^{'}$ is the standardized value of the indicator, and min ($X_{j}$) and max ($X_{j}$) is the minimum and maximum values of the *j*-th indicator. |
| Step 2 | $p_{ij}=\frac{X_{ij}^{'}}{\sum_{i=1}^{n} X_{ij}^{'}}$ | $p_{ij}$ is the proportion of the *i*-th study county under the *j*-th indicator in the total countries. n is the total number of counties. |
| Step 3 | $e_{j}=-k\sum_{i=1}^{n} p_{ij}\ln\left( p_{ij} \right)$  $k=\frac{1}{ln(n)}$ | $e_{j}$is the entropy value of the *j*-th indicator. |
| Step 4 | $d_{j}=1-e_{j}$ | $d_{j}$is the diversity factor or information utility value of the *j*-th indicator. |
| Step 5 | $w_{j}=\frac{d_{j}}{\sum_{j=1}^{m} d_{j}}$ | $w_{j}$is the weight value of the *j*-th indicator. |
| Step 6 | Repeat steps 1 to 5 for all indicators in each year during 2012-2019. | |

# Table S2: Characteristics of single urbanization indicators

| **Indicators** | **2012** | **2013** | **2014** | **2015** | **2016** | **2017** | **2018** | **2019** |
| --- | --- | --- | --- | --- | --- | --- | --- | --- |
| **Median (IQR)** |  |  |  |  |  |  |  |  |
| Proportion of urban population | 57.12 (38.76, 75.69) | 57.26 (39.21, 76.15) | 57.41 (39.67, 76.6) | 57.58 (40.14, 76.89) | 58 (40.67, 77.17) | 58.27 (41.03, 77.3) | 58.89 (41.45, 77.55) | 59.54 (41.89, 77.79) |
| Proportion of population using improved sanitation | 93.09 (63.97, 99.03) | 93.42 (65.71, 99.2) | 93.77 (67.05, 99.34) | 94.53 (68.23, 99.42) | 95.19 (69.21, 99.46) | 95.5 (70.93, 99.47) | 95.76 (72.38, 99.59) | 95.98 (73.97, 99.76) |
| Nighttime light | 0.11 (0.03, 0.23) | 0.11 (0.06, 0.22) | 0.16 (0.03, 0.35) | 0.15 (0.07, 0.36) | 0.1 (0.01, 0.36) | 0.3 (0.21, 0.56) | 0.28 (0.2, 0.56) | 0.26 (0.16, 0.54) |
| NDVI | 0.55 (0.35, 0.66) | 0.55 (0.36, 0.66) | 0.57 (0.35, 0.67) | 0.56 (0.36, 0.66) | 0.56 (0.35, 0.67) | 0.55 (0.35, 0.66) | 0.56 (0.35, 0.67) | 0.56 (0.36, 0.67) |
| GDP (per capita) | 5979.12 (1761.11, 15308.86) | 6203.2 (1881.78, 15925.43) | 6247.71 (2003.03, 16301.99) | 5538.57 (1991.87, 14939.46) | 5540.21 (1982.31, 15604.77) | 5908.87 (2004.07, 16317.54) | 6127.28 (2002.34, 17082.06) | 6171.14 (2092.74, 17345.49) |

NDVI: normalized difference vegetation index; GDP: gross domestic product.

# Table S3: Urbanization score in 178 countries and territories from 2012 to 2019

| Country name | 2012 | 2013 | 2014 | 2015 | 2016 | 2017 | 2018 | 2019 | EAPC (95% CI) |
| --- | --- | --- | --- | --- | --- | --- | --- | --- | --- |
| Afghanistan | 2.45 | 2.50 | 2.26 | 2.36 | 2.22 | 2.87 | 2.84 | 2.84 | 2.79 (-0.57, 6.26) |
| Albania | 8.82 | 8.82 | 8.83 | 8.84 | 8.64 | 10.52 | 10.92 | 10.42 | 3.31 (0.87, 5.80) * |
| Algeria | 6.80 | 6.78 | 6.30 | 6.51 | 6.06 | 7.42 | 7.66 | 7.01 | 1.50 (-1.37, 4.45) |
| Angola | 8.19 | 8.35 | 7.99 | 7.94 | 7.46 | 9.56 | 9.66 | 8.74 | 1.99 (-1.16, 5.25) |
| Antigua and Barbuda | 21.42 | 22.82 | 17.49 | 21.11 | 20.08 | 22.85 | 22.27 | 21.91 | 0.95 (-2.51, 4.52) |
| Argentina | 11.10 | 10.95 | 10.46 | 11.59 | 10.78 | 13.55 | 12.84 | 11.57 | 2.16 (-0.82, 5.22) |
| Armenia | 7.81 | 8.05 | 7.46 | 7.77 | 7.39 | 8.89 | 9.40 | 8.54 | 2.25 (-0.47, 5.05) |
| Australia | 22.38 | 21.32 | 19.08 | 20.55 | 18.08 | 22.38 | 22.70 | 20.63 | 0.11 (-3.15, 3.48) |
| Austria | 18.65 | 18.10 | 17.79 | 18.30 | 17.64 | 21.37 | 22.13 | 20.24 | 2.52 (-0.09, 5.20) |
| Azerbaijan | 8.29 | 8.48 | 7.85 | 8.20 | 7.48 | 8.98 | 9.38 | 8.58 | 1.27 (-1.38, 3.99) |
| Bahamas | 52.09 | 48.16 | 31.58 | 31.55 | 31.48 | 37.02 | 38.04 | 37.07 | -3.61 (-10.09, 3.35) |
| Bahrain | 14.57 | 14.79 | 12.76 | 13.77 | 13.32 | 15.94 | 16.54 | 16.15 | 2.30 (-0.86, 5.57) |
| Bangladesh | 7.11 | 7.67 | 6.91 | 7.56 | 7.62 | 9.74 | 10.08 | 9.83 | 5.72 (2.46, 9.08) * |
| Barbados | 15.09 | 15.44 | 12.37 | 13.76 | 13.08 | 15.76 | 15.44 | 14.76 | 0.62 (-2.90, 4.27) |
| Belarus | 11.39 | 11.09 | 10.64 | 10.62 | 9.86 | 12.54 | 12.92 | 11.94 | 1.82 (-1.45, 5.20) |
| Belgium | 29.92 | 30.31 | 27.82 | 28.99 | 27.74 | 33.73 | 35.68 | 33.79 | 2.66 (-0.29, 5.69) |
| Belize | 9.62 | 9.53 | 9.21 | 9.67 | 9.30 | 11.41 | 11.51 | 10.33 | 2.47 (-0.24, 5.25) |
| Benin | 5.50 | 5.48 | 5.47 | 5.39 | 5.36 | 6.82 | 6.90 | 6.36 | 3.41 (0.40, 6.52) * |
| Bhutan | 7.19 | 7.32 | 7.07 | 7.55 | 7.27 | 9.23 | 9.24 | 8.64 | 3.90 (1.11, 6.77) * |
| Bolivia | 8.99 | 9.66 | 9.48 | 9.88 | 10.00 | 11.84 | 11.71 | 11.07 | 3.75 (1.75, 5.80) * |
| Bosnia and Herzegovina | 8.98 | 8.76 | 8.83 | 8.83 | 8.60 | 10.44 | 10.84 | 9.96 | 2.74 (0.18, 5.37) * |
| Botswana | 7.51 | 7.46 | 7.56 | 7.58 | 7.34 | 9.65 | 9.47 | 8.50 | 3.34 (0.08, 6.71) * |
| Brazil | 12.30 | 12.29 | 11.76 | 11.87 | 11.30 | 14.05 | 14.04 | 12.94 | 1.81 (-0.95, 4.66) |
| Brunei Darussalam | 20.38 | 19.07 | 17.84 | 17.39 | 15.84 | 19.32 | 19.97 | 18.59 | -0.32 (-3.59, 3.07) |
| Bulgaria | 10.44 | 10.34 | 10.27 | 10.49 | 10.10 | 12.34 | 12.95 | 11.92 | 3.10 (0.49, 5.78) * |
| Burkina Faso | 3.84 | 3.96 | 3.76 | 3.97 | 3.95 | 4.99 | 5.23 | 4.83 | 4.67 (1.61, 7.82) * |
| Burundi | 4.65 | 4.67 | 4.51 | 4.58 | 4.37 | 5.49 | 5.58 | 5.17 | 2.62 (-0.24, 5.56) |
| Cabo Verde | 6.30 | 6.42 | 5.93 | 6.35 | 6.15 | 7.41 | 7.50 | 6.84 | 2.40 (-0.22, 5.09) |
| Cambodia | 5.47 | 5.43 | 5.49 | 5.61 | 5.47 | 7.23 | 7.04 | 6.52 | 4.05 (0.87, 7.33) * |
| Cameroon | 7.30 | 7.31 | 7.40 | 7.56 | 7.16 | 9.00 | 9.13 | 8.25 | 3.02 (0.29, 5.82) * |
| Canada | 19.48 | 18.33 | 17.56 | 19.35 | 17.79 | 21.44 | 20.68 | 19.92 | 1.53 (-0.94, 4.06) |
| Central African Republic | 5.78 | 5.85 | 5.80 | 5.95 | 5.67 | 7.27 | 7.31 | 6.61 | 3.26 (0.30, 6.31) * |
| Chad | 1.87 | 1.87 | 1.67 | 1.71 | 1.56 | 2.03 | 2.18 | 1.92 | 1.75 (-2.32, 5.99) |
| Chile | 11.46 | 11.45 | 10.51 | 11.15 | 10.79 | 13.21 | 13.38 | 12.09 | 2.18 (-0.75, 5.19) |
| China | 7.46 | 7.61 | 7.42 | 8.20 | 8.05 | 10.43 | 11.04 | 10.55 | 6.51 (3.41, 9.70) * |
| Colombia | 15.05 | 15.37 | 13.11 | 14.46 | 13.96 | 17.25 | 17.63 | 15.14 | 1.82 (-1.83, 5.61) |
| Comoros | 6.42 | 6.34 | 6.24 | 6.23 | 6.11 | 7.68 | 7.74 | 6.95 | 2.58 (-0.40, 5.65) |
| Congo | 8.69 | 8.68 | 8.39 | 8.46 | 8.12 | 10.09 | 10.52 | 9.54 | 2.57 (-0.38, 5.60) |
| Costa Rica | 12.30 | 12.36 | 11.92 | 12.89 | 12.40 | 15.30 | 15.52 | 14.31 | 3.53 (0.90, 6.23) * |
| Croatia | 7.06 | 7.30 | 7.14 | 7.37 | 7.17 | 9.00 | 9.17 | 8.31 | 3.58 (0.80, 6.43) * |
| Cuba | 11.27 | 11.08 | 10.82 | 10.93 | 10.59 | 12.99 | 13.56 | 12.61 | 2.79 (0.02, 5.64) * |
| Cyprus | 11.00 | 11.04 | 10.76 | 11.34 | 11.06 | 13.43 | 13.75 | 12.71 | 3.33 (0.88, 5.84) * |
| Czechia | 16.53 | 15.84 | 14.04 | 14.99 | 14.41 | 17.52 | 18.83 | 17.81 | 2.42 (-1.21, 6.18) |
| Cote d'Ivoire | 22.26 | 19.24 | 19.15 | 19.17 | 19.38 | 23.59 | 22.92 | 21.43 | 1.49 (-1.81, 4.90) |
| Democratic Republic of the Congo | 6.63 | 6.72 | 6.49 | 6.73 | 6.43 | 7.99 | 8.34 | 7.46 | 3.00 (0.12, 5.96) |
| Denmark | 22.81 | 21.96 | 21.65 | 22.64 | 21.43 | 26.31 | 27.04 | 25.13 | 2.71 (0.06, 5.44) * |
| Djibouti | 5.13 | 5.31 | 5.13 | 5.52 | 5.31 | 6.55 | 6.80 | 6.27 | 4.04 (1.47, 6.69) * |
| Dominica | 11.06 | 11.11 | 10.83 | 11.49 | 11.09 | 13.00 | 13.23 | 12.65 | 2.81 (0.89, 4.76) * |
| Dominican Republic | 11.22 | 11.20 | 10.64 | 11.20 | 11.17 | 13.89 | 13.92 | 12.73 | 3.35 (0.42, 6.36) * |
| Ecuador | 10.09 | 10.02 | 9.72 | 10.22 | 10.00 | 12.41 | 12.21 | 11.08 | 2.84 (0.07, 5.68) * |
| Egypt | 5.15 | 5.07 | 4.65 | 5.01 | 4.65 | 5.41 | 5.49 | 5.18 | 0.99 (-1.31, 3.34) |
| El Salvador | 10.04 | 10.14 | 9.84 | 10.32 | 9.93 | 12.24 | 12.37 | 11.49 | 3.08 (0.54, 5.68) * |
| Equatorial Guinea | 13.20 | 12.84 | 12.05 | 11.09 | 10.42 | 12.66 | 13.10 | 11.71 | -0.77 (-4.06, 2.63) |
| Estonia | 12.08 | 12.06 | 12.06 | 12.9 | 11.83 | 15.55 | 15.27 | 14.93 | 4.06 (1.16, 7.03) * |
| Eswatini | 7.11 | 7.23 | 6.55 | 6.58 | 6.22 | 8.25 | 8.57 | 7.64 | 2.40 (-1.63, 6.60) |
| Ethiopia | 2.54 | 2.63 | 2.56 | 2.68 | 2.57 | 3.31 | 3.54 | 3.16 | 4.54 (1.27, 7.90) * |
| Finland | 19.26 | 18.67 | 18.23 | 18.91 | 18.24 | 21.68 | 22.62 | 20.81 | 2.39 (-0.06, 4.90) |
| France | 18.33 | 18.06 | 17.52 | 18.01 | 17.18 | 20.88 | 21.38 | 19.77 | 2.23 (-0.32, 4.84) |
| Gabon | 11.51 | 11.47 | 10.95 | 11.20 | 10.68 | 13.28 | 13.71 | 12.46 | 2.38 (-0.55, 5.39) |
| Gambia | 6.51 | 6.46 | 6.02 | 6.41 | 6.09 | 7.48 | 7.58 | 6.95 | 2.23 (-0.57, 5.11) |
| Georgia | 8.67 | 8.69 | 8.67 | 8.82 | 8.52 | 10.21 | 10.61 | 9.69 | 2.69 (0.37, 5.08) * |
| Germany | 21.24 | 19.14 | 20.36 | 20.24 | 19.4 | 23.44 | 23.92 | 22.09 | 2.13 (-0.55, 4.88) |
| Ghana | 7.10 | 7.33 | 7.06 | 7.22 | 6.94 | 8.86 | 9.16 | 8.29 | 3.44 (0.36, 6.62) |
| Greece | 13.68 | 13.33 | 12.91 | 13.13 | 12.46 | 15.11 | 15.54 | 14.33 | 1.82 (-0.81, 4.51) |
| Grenada | 9.81 | 9.66 | 9.21 | 9.98 | 9.62 | 11.98 | 11.79 | 10.88 | 2.98 (0.17, 5.88) * |
| Guatemala | 8.72 | 8.74 | 8.48 | 9.05 | 8.61 | 10.84 | 10.85 | 9.89 | 3.21 (0.39, 6.10) * |
| Guinea | 5.67 | 5.76 | 5.82 | 5.86 | 5.64 | 7.19 | 7.16 | 6.67 | 3.43 (0.69, 6.23) * |
| Guinea-Bissau | 5.50 | 5.66 | 5.53 | 5.78 | 5.60 | 7.10 | 7.17 | 6.59 | 3.86 (1.05, 6.75) * |
| Guyana | 8.90 | 8.87 | 8.48 | 8.91 | 8.53 | 10.75 | 10.62 | 9.82 | 2.72 (-0.11, 5.64) |
| Haiti | 7.22 | 7.02 | 6.79 | 7.25 | 6.99 | 8.96 | 9.06 | 8.59 | 4.00 (0.89, 7.21) * |
| Honduras | 8.91 | 8.99 | 8.64 | 9.08 | 8.72 | 10.89 | 10.98 | 10.03 | 3.00 (0.22, 5.85) * |
| Hungary | 11.98 | 11.87 | 11.96 | 12.07 | 11.80 | 14.49 | 15.10 | 14.13 | 3.54 (0.96, 6.17) * |
| Iceland | 17.75 | 17.13 | 18.29 | 20.43 | 21.00 | 27.35 | 27.34 | 23.63 | 6.86 (3.02, 10.85) * |
| India | 5.46 | 5.77 | 5.62 | 5.96 | 5.64 | 7.42 | 7.76 | 7.69 | 5.70 (2.62, 8.88) * |
| Indonesia | 9.32 | 9.50 | 8.90 | 9.39 | 9.33 | 11.23 | 11.52 | 10.64 | 3.12 (0.53, 5.78) * |
| Iran | 8.21 | 7.92 | 7.21 | 7.86 | 7.65 | 9.15 | 8.87 | 8.38 | 1.69 (-1.03, 4.48) |
| Iraq | 7.53 | 7.80 | 7.02 | 6.91 | 6.60 | 8.40 | 9.16 | 8.95 | 3.04 (-1.01, 7.25) |
| Ireland | 19.91 | 19.48 | 19.87 | 23.86 | 22.84 | 28.74 | 30.49 | 29.12 | 7.36 (4.45, 10.36) * |
| Israel | 15.88 | 16.70 | 15.63 | 22.77 | 25.70 | 31.81 | 31.13 | 29.61 | 12.27 (7.00, 17.81) * |
| Italy | 79.74 | 79.51 | 80.11 | 79.32 | 80.29 | 76.32 | 75.93 | 77.96 | -0.62 (-1.26, 0.02) |
| Jamaica | 13.38 | 13.45 | 11.65 | 12.12 | 12.23 | 14.58 | 14.56 | 13.64 | 1.46 (-1.67, 4.68) |
| Japan | 20.83 | 18.44 | 17.12 | 18.3 | 18.37 | 21.73 | 21.86 | 20.61 | 1.80 (-1.53, 5.23) |
| Jordan | 7.54 | 7.62 | 7.16 | 7.66 | 7.24 | 8.80 | 8.93 | 8.31 | 2.47 (-0.06, 5.06) |
| Kazakhstan | 8.27 | 8.63 | 7.86 | 7.93 | 7.10 | 8.86 | 8.89 | 8.41 | 0.62 (-2.34, 3.67) |
| Kenya | 4.00 | 4.19 | 3.89 | 4.17 | 3.84 | 4.73 | 5.49 | 4.69 | 3.59 (-0.02, 7.32) |
| Kiribati | 4.73 | 4.74 | 4.50 | 4.77 | 4.63 | 5.76 | 5.82 | 5.32 | 3.08 (0.24, 6.00) * |
| Kuwait | 23.11 | 21.61 | 17.44 | 16.6 | 15.33 | 18.31 | 19.46 | 17.62 | -2.76 (-7.30, 2.00) |
| Kyrgyzstan | 4.81 | 4.99 | 4.54 | 4.81 | 4.6 | 5.63 | 5.58 | 5.37 | 2.33 (-0.30, 5.03) |
| Laos | 7.11 | 7.28 | 7.20 | 7.65 | 7.46 | 9.27 | 9.37 | 8.44 | 3.88 (1.18, 6.64) * |
| Latvia | 11.59 | 11.47 | 11.42 | 12.07 | 11.25 | 14.11 | 14.67 | 14.01 | 3.79 (1.10, 6.55) * |
| Lebanon | 12.24 | 12.94 | 10.69 | 12.12 | 11.56 | 13.95 | 15.31 | 13.97 | 3.04 (-0.69, 6.91) |
| Lesotho | 4.03 | 4.00 | 3.91 | 4.01 | 3.94 | 5.10 | 5.28 | 4.70 | 3.95 (0.54, 7.47) * |
| Liberia | 7.32 | 7.16 | 6.83 | 7.29 | 7.03 | 8.69 | 8.90 | 8.00 | 2.89 (-0.06, 5.93) |
| Libya | 9.23 | 8.48 | 7.41 | 7.74 | 7.28 | 9.45 | 9.86 | 8.81 | 1.32 (-3.15, 5.99) |
| Lithuania | 11.79 | 11.52 | 11.28 | 11.80 | 11.65 | 13.96 | 14.74 | 14.17 | 3.81 (1.31, 6.38) * |
| Luxembourg | 36.69 | 35.8 | 34.37 | 35.65 | 34.33 | 40.69 | 41.96 | 38.92 | 2.01 (-0.40, 4.48) |
| Madagascar | 5.84 | 4.50 | 6.91 | 4.60 | 4.63 | 6.94 | 5.71 | 6.28 | 2.06 (-4.92, 9.55) |
| Malawi | 3.78 | 3.79 | 3.68 | 3.76 | 3.62 | 5.01 | 5.04 | 4.73 | 4.73 (0.84, 8.77) * |
| Malaysia | 12.94 | 12.51 | 12.53 | 13.22 | 12.44 | 15.5 | 15.91 | 14.66 | 3.22 (0.48, 6.02) * |
| Maldives | 6.80 | 7.07 | 6.69 | 7.75 | 7.05 | 8.82 | 8.93 | 8.45 | 4.15 (1.45, 6.93) * |
| Mali | 3.56 | 3.58 | 3.34 | 3.66 | 3.46 | 4.36 | 4.59 | 4.19 | 3.80 (0.65, 7.04) * |
| Malta | 16.69 | 16.85 | 15.80 | 17.00 | 16.42 | 20.47 | 21.53 | 20.14 | 3.99 (1.00, 7.06) * |
| Marshall Islands | 8.25 | 7.79 | 7.29 | 7.94 | 7.53 | 9.71 | 10.10 | 8.84 | 3.13 (-0.66, 7.06) |
| Mauritania | 3.66 | 3.79 | 3.45 | 3.76 | 3.59 | 4.55 | 4.75 | 4.37 | 3.83 (0.61, 7.16) * |
| Mauritius | 13.88 | 13.96 | 11.79 | 12.02 | 10.99 | 13.60 | 14.43 | 15.04 | 1.27 (-3.04, 5.77) |
| Mexico | 10.34 | 10.37 | 10.10 | 10.63 | 9.83 | 12.11 | 12.36 | 11.35 | 2.4 (-0.16, 5.02) |
| Micronesia (Federated States of) | 7.76 | 7.57 | 7.56 | 7.66 | 7.75 | 9.52 | 9.62 | 8.66 | 3.23 (0.42, 6.12) * |
| Mongolia | 6.67 | 6.73 | 6.36 | 6.52 | 6.09 | 7.45 | 7.83 | 7.35 | 2.22 (-0.57, 5.08) |
| Montenegro | 9.92 | 9.81 | 9.82 | 9.84 | 9.63 | 11.69 | 12.12 | 11.29 | 2.97 (0.48, 5.53) * |
| Morocco | 6.26 | 6.35 | 5.87 | 6.26 | 6.21 | 8.29 | 8.18 | 7.23 | 4.00 (0.13, 8.02) * |
| Mozambique | 4.91 | 5.00 | 4.97 | 4.94 | 4.72 | 6.43 | 6.43 | 5.85 | 3.90 (0.27, 7.67) * |
| Namibia | 5.00 | 4.65 | 4.63 | 4.67 | 4.38 | 5.91 | 5.97 | 5.12 | 2.51 (-1.52, 6.07) |
| Nauru | 13.61 | 13.99 | 12.74 | 13.39 | 13.29 | 16.06 | 16.18 | 15.74 | 2.93 (0.28, 5.66) * |
| Nepal | 4.92 | 5.00 | 4.81 | 5.25 | 4.92 | 6.36 | 6.46 | 6.00 | 4.18 (1.15, 7.30) * |
| Netherlands | 23.13 | 21.70 | 22.11 | 23.07 | 22.01 | 26.86 | 27.51 | 26.42 | 3.21 (0.68, 5.80) * |
| Nicaragua | 8.64 | 8.60 | 8.25 | 8.66 | 8.33 | 10.45 | 10.38 | 9.42 | 2.67 (-0.21, 5.63) |
| Niger | 0.85 | 0.87 | 0.77 | 0.86 | 0.72 | 1.05 | 1.17 | 0.92 | 3.29 (-2.29, 9.18) |
| Nigeria | 6.05 | 6.18 | 6.01 | 6.14 | 5.78 | 7.22 | 7.45 | 6.84 | 2.75 (-0.01, 5.59) |
| North Macedonia | 9.15 | 8.94 | 8.94 | 9.09 | 8.85 | 10.5 | 10.93 | 9.88 | 2.41 (0.04, 4.84) * |
| Norway | 29.64 | 28.46 | 26.03 | 24.92 | 23.13 | 27.91 | 29.39 | 26.26 | -0.65 (-4.03, 2.84) |
| Oman | 11.34 | 10.82 | 10.05 | 10.30 | 9.36 | 11.43 | 11.98 | 10.80 | 0.55 (-2.61, 3.80) |
| Pakistan | 3.74 | 3.87 | 3.59 | 3.88 | 3.70 | 4.72 | 4.76 | 4.45 | 3.67 (0.63, 6.79) * |
| Palau | 12.85 | 12.78 | 12.56 | 14.00 | 13.59 | 16.33 | 16.29 | 14.98 | 3.69 (1.25, 6.19) * |
| Panama | 11.29 | 11.33 | 11.13 | 12.06 | 11.80 | 14.80 | 14.80 | 13.67 | 4.26 (1.53, 7.08) * |
| Papua New Guinea | 7.13 | 6.64 | 5.49 | 5.83 | 5.42 | 6.63 | 6.72 | 6.07 | -0.68 (-4.66, 3.47) |
| Paraguay | 9.67 | 9.52 | 9.35 | 9.84 | 9.20 | 11.36 | 11.65 | 10.51 | 2.55 (-0.15, 5.32) |
| Peru | 10.2 | 10.21 | 9.71 | 10.08 | 9.77 | 12.18 | 12.22 | 11.21 | 2.67 (-0.17, 5.59) |
| Philippines | 8.93 | 8.75 | 8.55 | 8.88 | 8.58 | 10.76 | 10.77 | 9.71 | 2.75 (-0.15, 5.74) |
| Poland | 11.44 | 10.93 | 11.16 | 11.43 | 11.26 | 13.46 | 14.10 | 12.95 | 3.25 (0.78, 5.78) * |
| Portugal | 12.72 | 13.05 | 12.49 | 12.84 | 12.56 | 15.14 | 15.81 | 14.72 | 3.06 (0.57, 5.61) * |
| Qatar | 29.75 | 28.79 | 25.86 | 25.79 | 23.02 | 28.35 | 34.24 | 35.78 | 2.80 (-2.55, 8.44) |
| Republic of Korea | 20.78 | 18.91 | 17.24 | 19.97 | 18.80 | 22.99 | 23.77 | 20.40 | 2.19 (-1.57, 6.09) |
| Republic of Moldova | 6.98 | 7.02 | 6.77 | 6.60 | 6.57 | 8.29 | 8.40 | 7.77 | 2.73 (-0.39, 5.94) |
| Romania | 9.39 | 9.31 | 9.25 | 9.40 | 9.27 | 11.45 | 11.9 | 10.98 | 3.56 (0.83, 6.37) * |
| Russian Federation | 10.53 | 10.29 | 9.74 | 9.58 | 8.99 | 11.02 | 11.21 | 10.61 | 0.94 (-1.96, 3.93) |
| Rwanda | 5.61 | 5.70 | 5.43 | 5.70 | 5.33 | 6.64 | 7.01 | 6.33 | 2.93 (-0.03, 5.97) |
| Saint Kitts and Nevis | 11.53 | 11.35 | 10.73 | 11.60 | 11.29 | 14.12 | 13.98 | 13.08 | 3.29 (0.40, 6.27) * |
| Saint Lucia | 9.51 | 9.64 | 8.94 | 9.61 | 9.25 | 11.65 | 11.65 | 10.76 | 3.10 (0.12, 6.17) * |
| Saint Vincent and the Grenadines | 10.04 | 9.96 | 9.50 | 10.06 | 9.72 | 12.15 | 12.1 | 11.37 | 3.08 (0.29, 5.94) * |
| Sao Tome and Principe | 7.11 | 7.50 | 6.63 | 7.60 | 7.73 | 9.18 | 9.85 | 9.20 | 5.08 (2.06, 8.18) * |
| Saudi Arabia | 11.84 | 11.45 | 10.64 | 11.16 | 10.29 | 12.54 | 13.55 | 12.47 | 1.94 (-1.28, 5.26) |
| Senegal | 5.42 | 5.43 | 5.05 | 5.36 | 5.19 | 6.43 | 6.55 | 6.01 | 2.85 (-0.08, 5.87) |
| Serbia | 10.27 | 10.19 | 10.02 | 9.95 | 9.89 | 11.87 | 13.05 | 11.80 | 3.29 (0.36, 6.31) * |
| Seychelles | 11.92 | 12.32 | 11.57 | 12.60 | 12.08 | 14.73 | 14.8 | 13.67 | 3.09 (0.57, 5.68) * |
| Sierra Leone | 6.59 | 6.66 | 6.49 | 6.65 | 6.26 | 8.06 | 8.20 | 7.35 | 2.89 (-0.25, 6.13) |
| Singapore | 35.86 | 21.56 | 31.62 | 35.93 | 34.49 | 42.44 | 42.07 | 41.30 | 6.35 (-0.48, 13.66) |
| Slovakia | 12.18 | 11.50 | 11.72 | 11.72 | 11.35 | 13.71 | 14.31 | 13.14 | 2.48 (-0.16, 5.19) |
| Slovenia | 15.33 | 14.02 | 13.82 | 14.35 | 14.00 | 17.21 | 18.16 | 16.58 | 2.99 (-0.25, 6.34) |
| Solomon Islands | 6.63 | 6.57 | 6.44 | 6.67 | 6.57 | 8.14 | 8.26 | 7.39 | 3.13 (0.30, 6.04) * |
| South Africa | 8.14 | 7.95 | 7.46 | 7.73 | 7.21 | 9.21 | 9.35 | 8.43 | 1.95 (-1.35, 5.36) |
| Spain | 59.63 | 67.15 | 53.06 | 55.50 | 52.03 | 60.64 | 62.81 | 66.73 | 0.94 (-2.90, 4.94) |
| Sri Lanka | 7.63 | 7.66 | 7.42 | 8.00 | 7.52 | 9.52 | 9.56 | 8.78 | 3.36 (0.53, 6.27) * |
| Sudan | 3.42 | 3.44 | 3.42 | 3.67 | 3.55 | 4.59 | 4.07 | 3.75 | 2.83 (-0.38, 6.16) |
| Suriname | 11.84 | 11.81 | 11.33 | 12.02 | 10.69 | 13.36 | 13.49 | 12.38 | 1.63 (-1.14, 4.47) |
| Sweden | 27.21 | 23.85 | 21.94 | 23.98 | 24.23 | 27.48 | 28.24 | 25.52 | 1.30 (-1.99, 4.70) |
| Switzerland | 27.06 | 25.98 | 25.22 | 27.97 | 26.07 | 30.64 | 30.91 | 28.71 | 2.16 (-0.12, 4.50) |
| Syrian Arab Republic | 5.75 | 5.50 | 5.08 | 5.34 | 4.83 | 6.05 | 6.31 | 6.20 | 1.98 (-1.46, 5.54) |
| Tajikistan | 4.08 | 4.18 | 3.82 | 3.93 | 3.55 | 4.41 | 4.43 | 4.24 | 1.05 (-1.82, 4.01) |
| Thailand | 9.33 | 9.36 | 8.82 | 9.42 | 9.04 | 11.52 | 11.60 | 10.80 | 3.46 (0.40, 6.61) * |
| Timor-Leste | 7.47 | 6.64 | 6.28 | 6.44 | 6.61 | 8.32 | 7.84 | 7.37 | 1.93 (-1.77, 5.78) |
| Togo | 5.31 | 5.37 | 5.30 | 5.41 | 5.16 | 6.66 | 6.87 | 6.15 | 3.51 (0.34, 6.79) * |
| Trinidad and Tobago | 16.50 | 16.16 | 14.17 | 15.01 | 14.21 | 17.24 | 17.21 | 16.03 | 0.77 (-2.31, 3.96) |
| Tunisia | 6.96 | 6.86 | 6.52 | 6.79 | 6.35 | 7.80 | 7.96 | 7.39 | 1.96 (-0.75, 4.73) |
| Turkey | 9.86 | 10.16 | 9.63 | 10.20 | 9.55 | 11.46 | 11.63 | 10.61 | 1.98 (-0.38, 4.39) |
| Turkmenistan | 6.24 | 6.39 | 6.03 | 6.28 | 5.89 | 7.14 | 7.23 | 7.22 | 2.51 (0.11, 4.97) * |
| Uganda | 4.31 | 4.39 | 4.19 | 4.44 | 4.14 | 5.20 | 5.48 | 4.90 | 3.12 (0.11, 6.22) * |
| Ukraine | 9.95 | 11.65 | 9.69 | 9.61 | 9.16 | 11.00 | 11.29 | 10.46 | 0.64 (-2.78, 4.17) |
| United Arab Emirates | 15.50 | 15.25 | 14.44 | 15.40 | 14.37 | 17.65 | 18.36 | 16.97 | 2.53 (-0.28, 5.42) |
| United Kingdom | 19.18 | 18.38 | 18.74 | 20.47 | 19.15 | 21.82 | 22.46 | 20.99 | 2.44 (0.56, 4.35) * |
| United Republic of Tanzania | 4.59 | 4.66 | 4.55 | 4.70 | 4.60 | 5.88 | 6.28 | 5.75 | 4.65 (1.45, 7.95) * |
| United States of America | 20.14 | 20.00 | 19.20 | 22.10 | 21.03 | 25.17 | 25.63 | 24.47 | 4.09 (1.71, 6.53) * |
| Uruguay | 13.45 | 13.75 | 13.22 | 13.68 | 13.46 | 16.89 | 16.70 | 15.35 | 3.16 (0.37, 6.04) * |
| Uzbekistan | 5.47 | 5.61 | 5.24 | 5.59 | 5.24 | 6.20 | 6.01 | 5.77 | 1.39 (-0.66, 3.47) |
| Vanuatu | 8.04 | 7.93 | 7.69 | 7.86 | 7.67 | 9.51 | 9.48 | 8.63 | 2.41 (-0.37, 5.28) |
| Viet Nam | 7.52 | 7.55 | 7.35 | 7.83 | 7.48 | 9.43 | 9.63 | 9.07 | 3.92 (1.17, 6.74) * |
| Yemen | 3.14 | 3.11 | 2.86 | 3.07 | 2.77 | 3.50 | 3.60 | 3.24 | 1.74 (-1.49, 5.09) |
| Zambia | 6.17 | 6.06 | 5.83 | 6.04 | 5.77 | 7.49 | 7.51 | 6.70 | 2.83 (-0.53, 6.31) |
| Zimbabwe | 5.16 | 5.21 | 5.07 | 5.16 | 4.82 | 6.53 | 6.29 | 5.45 | 2.43 (-1.27, 6.27) |

Countries were arranged in alphabetical order.

**P* for EAPC was <0.05.

EAPC: estimated annual percentage change.

# Table S4: Estimated annual percentage change (EAPC, %) for incidence of TB from 2012 to 2019

| Country name | Total TB | | |  | DS-TB | | |  | MDR-TB | | |  | XDR-TB | | |
| --- | --- | --- | --- | --- | --- | --- | --- | --- | --- | --- | --- | --- | --- | --- | --- |
|  | 2012^a^ | 2019^a^ | EAPC (95% CI) |  | 2012^a^ | 2019^a^ | EAPC (95% CI) |  | 2012^a^ | 2019^a^ | EAPC (95% CI) |  | 2012^b^ | 2019 ^b^ | EAPC (95% CI) |
| Afghanistan | 97.11 | 85.09 | -1.28 (-2.15, -0.39) * |  | 91.24 | 81.21 | -1.07 (-1.91, -0.22) * |  | 5.73 | 3.74 | -5.06 (-6.78, -3.31) * |  | 14.27 | 13.21 | -0.34 (-1.69, 1.02) |
| Albania | 3.61 | 2.92 | -3.36 (-4.29, -2.42) * |  | 3.56 | 2.90 | -3.28 (-4.20, -2.36) * |  | 0.05 | 0.02 | -10.27 (-13.13, -7.32) * |  | 0.70 | 0.49 | -5.12 (-7.70, -2.47) * |
| Algeria | 19.61 | 17.23 | -1.96 (-2.18, -1.75) * |  | 19.26 | 16.87 | -2.00 (-2.21, -1.80) * |  | 0.34 | 0.35 | 0.10 (-1.14, 1.35) |  | 0.89 | 1.23 | 4.41 (2.94, 5.91) * |
| Angola | 313.94 | 265.58 | -2.26 (-2.53, -1.99) * |  | 305.21 | 257.90 | -2.28 (-2.56, -1.99) * |  | 8.69 | 7.62 | -1.78 (-1.88, -1.68) * |  | 4.13 | 4.92 | 2.54 (2.24, 2.84) * |
| Antigua and Barbuda | 23.94 | 18.83 | -3.97 (-5.44, -2.49) * |  | 23.79 | 18.68 | -3.99 (-5.46, -2.50) * |  | 0.14 | 0.13 | -1.53 (-3.13, 0.11) |  | 0.80 | 1.06 | 2.93 (0.96, 4.93) * |
| Argentina | 12.11 | 12.10 | -0.30 (-1.48, 0.90) |  | 11.88 | 11.89 | -0.28 (-1.48, 0.93) |  | 0.21 | 0.19 | -1.52 (-1.84, -1.20) * |  | 1.89 | 2.37 | 3.32 (2.75, 3.90) * |
| Armenia | 33.97 | 23.23 | -4.86 (-6.24, -3.46) * |  | 26.97 | 19.00 | -4.5 0(-5.65, -3.34) * |  | 6.11 | 3.47 | -7.18 (-9.53, -4.76) * |  | 88.88 | 76.09 | -1.64 (-3.52, 0.27) |
| Australia | 5.56 | 5.24 | -0.94 (-1.20, -0.69) * |  | 5.42 | 5.05 | -1.10 (-1.38, -0.83) * |  | 0.12 | 0.16 | 3.97 (2.77, 5.19) * |  | 1.09 | 2.05 | 8.92 (7.51, 10.34) * |
| Austria | 6.79 | 5.93 | -1.93 (-2.58, -1.29) * |  | 6.47 | 5.72 | -1.76 (-2.42, -1.10) * |  | 0.29 | 0.19 | -6.46 (-6.88, -6.04) * |  | 2.53 | 2.33 | -1.49 (-2.03, -0.94) * |
| Azerbaijan | 55.66 | 48.08 | -1.66 (-2.33, -0.99) * |  | 44.10 | 36.15 | -2.36 (-3.00, -1.72) * |  | 10.02 | 9.79 | -0.04 (-1.08, 1.01) |  | 154.43 | 214.43 | 5.02 (3.77, 6.28) * |
| Bahamas | 13.30 | 11.86 | -1.59 (-1.69, -1.50) * |  | 13.11 | 11.66 | -1.61 (-1.77, -1.46) * |  | 0.18 | 0.18 | -0.41 (-4.37, 3.72) |  | 1.07 | 1.45 | 3.50 (-0.49, 7.65) |
| Bahrain | 20.81 | 16.69 | -2.97 (-3.18, -2.75) * |  | 20.15 | 16.20 | -2.91 (-3.24, -2.59) * |  | 0.64 | 0.48 | -4.6 (-7.88, -1.20) * |  | 1.67 | 1.68 | -0.50 (-4.09, 3.22) |
| Bangladesh | 183.47 | 139.38 | -3.73 (-3.94, -3.52) * |  | 178.00 | 134.23 | -3.83 (-4.03, -3.63) * |  | 5.38 | 5.02 | -0.86 (-1.42, -0.30) * |  | 9.48 | 12.31 | 3.89 (3.69, 4.10) * |
| Barbados | 7.71 | 6.27 | -3.23 (-4.10, -2.36) * |  | 7.69 | 6.24 | -3.25 (-4.12, -2.37) * |  | 0.02 | 0.02 | 0.50 (-1.09, 2.11) |  | 0.11 | 0.17 | 5.12 (3.10, 7.18) * |
| Belarus | 36.09 | 23.56 | -5.51 (-7.14, -3.85) * |  | 21.48 | 12.97 | -6.55 (-8.58, -4.47) * |  | 12.72 | 8.69 | -4.93 (-6.02, -3.82) * |  | 188.40 | 190.49 | 0.46 (-0.22, 1.15) |
| Belgium | 8.06 | 7.37 | -1.35 (-1.46, -1.23) * |  | 7.88 | 7.22 | -1.31 (-1.43, -1.19) * |  | 0.17 | 0.13 | -3.54 (-4.62, -2.44) * |  | 1.49 | 1.66 | 1.41 (0.62, 2.21) * |
| Belize | 28.61 | 24.66 | -1.91 (-2.62, -1.20) * |  | 28.49 | 24.53 | -1.93 (-2.65, -1.20) * |  | 0.11 | 0.12 | 1.43 (-0.95, 3.88) |  | 0.61 | 0.97 | 6.11 (3.29, 9.00) * |
| Benin | 176.34 | 147.51 | -2.35 (-2.86, -1.83) * |  | 173.70 | 144.73 | -2.40 (-2.92, -1.88) * |  | 2.63 | 2.76 | 0.98 (0.56, 1.41) * |  | 1.22 | 1.78 | 5.76 (5.26, 6.26) * |
| Bhutan | 126.12 | 99.22 | -3.18 (-3.46, -2.89) * |  | 120.96 | 95.12 | -3.17 (-3.47, -2.87) * |  | 5.07 | 4.01 | -3.36 (-3.55, -3.18) * |  | 9.12 | 9.82 | 0.95 (0.55, 1.36) * |
| Bolivia | 114.24 | 87.50 | -3.95 (-4.26, -3.63) * |  | 109.99 | 83.03 | -4.14 (-4.45, -3.82) * |  | 4.01 | 4.14 | -0.15 (-2.44, 2.19) |  | 22.97 | 32.73 | 4.32 (1.63, 7.08) * |
| Bosnia and Herzegovina | 14.92 | 12.42 | -2.36 (-2.68, -2.04) * |  | 14.83 | 12.35 | -2.35 (-2.67, -2.04) * |  | 0.08 | 0.06 | -4.69 (-6.04, -3.33) * |  | 1.25 | 1.24 | 0.30 (-0.86, 1.47) |
| Botswana | 383.97 | 345.19 | -1.24 (-1.63, -0.86) * |  | 369.77 | 328.53 | -1.40 (-1.79, -1.02) * |  | 14.14 | 16.55 | 2.46 (1.77, 3.15) * |  | 6.73 | 10.68 | 6.96 (6.03, 7.91) * |
| Brazil | 29.37 | 27.72 | -0.54 (-1.00, -0.07) * |  | 28.65 | 26.80 | -0.66 (-1.12, -0.19) * |  | 0.69 | 0.85 | 3.35 (2.66, 4.04) * |  | 3.91 | 6.72 | 8.29 (7.35, 9.24) * |
| Brunei Darussalam | 57.50 | 50.16 | -1.90 (-2.39, -1.41) * |  | 57.34 | 49.98 | -1.91 (-2.38, -1.43) * |  | 0.15 | 0.16 | 1.00 (-3.45, 5.65) |  | 1.34 | 2.06 | 5.54 (0.76, 10.54) * |
| Bulgaria | 18.16 | 14.10 | -3.53 (-3.66, -3.39) * |  | 17.27 | 13.69 | -3.25 (-3.47, -3.04) * |  | 0.78 | 0.34 | -11.07 (-12.7, -9.42) * |  | 11.26 | 7.36 | -5.60 (-6.68, -4.51) * |
| Burkina Faso | 222.35 | 194.73 | -1.64 (-2.09, -1.18) * |  | 215.54 | 189.05 | -1.62 (-2.09, -1.15) * |  | 6.77 | 5.64 | -2.18 (-3.28, -1.06) * |  | 3.16 | 3.64 | 2.36 (1.60, 3.14) * |
| Burundi | 515.91 | 482.36 | -0.64 (-1.20, -0.08) * |  | 502.76 | 467.55 | -0.71 (-1.29, -0.13) * |  | 13.09 | 14.71 | 1.80 (1.03, 2.58) * |  | 6.27 | 9.49 | 6.20 (5.22, 7.19) * |
| Cabo Verde | 122.73 | 93.53 | -3.51 (-4.02, -3.00) * |  | 118.94 | 90.39 | -3.56 (-4.08, -3.03) * |  | 3.77 | 3.12 | -2.12 (-3.12, -1.12) * |  | 1.81 | 2.01 | 2.03 (1.17, 2.89) * |
| Cambodia | 384.86 | 311.59 | -3.05 (-3.42, -2.68) * |  | 380.91 | 309.15 | -3.02 (-3.37, -2.68) * |  | 3.70 | 2.23 | -6.26 (-10.59, -1.73) * |  | 24.62 | 20.38 | -2.01 (-6.41, 2.59) |
| Cameroon | 185.16 | 153.57 | -2.21 (-2.93, -1.48) * |  | 180.67 | 150.03 | -2.19 (-2.92, -1.45) * |  | 4.48 | 3.52 | -2.88 (-3.87, -1.88) * |  | 2.09 | 2.27 | 1.59 (0.85, 2.33) * |
| Canada | 4.31 | 4.31 | -0.06 (-0.62, 0.50) |  | 4.25 | 4.25 | -0.07 (-0.63, 0.49) |  | 0.05 | 0.05 | -0.17 (-0.97, 0.63) |  | 0.47 | 0.67 | 4.62 (3.89, 5.36) * |
| Central African Republic | 560.27 | 506.25 | -1.45 (-1.55, -1.34) * |  | 553.00 | 498.84 | -1.47 (-1.57, -1.37) * |  | 7.24 | 7.36 | 0.17 (-0.28, 0.62) |  | 3.46 | 4.75 | 4.50 (3.84, 5.16) * |
| Chad | 246.59 | 213.37 | -1.92 (-2.37, -1.47) * |  | 239.51 | 207.66 | -1.90 (-2.37, -1.43) * |  | 7.04 | 5.67 | -2.83 (-3.27, -2.39) * |  | 3.30 | 3.66 | 1.63 (1.38, 1.87) * |
| Chile | 11.47 | 10.07 | -1.74 (-2.72, -0.75) * |  | 11.35 | 9.95 | -1.75 (-2.73, -0.77) * |  | 0.12 | 0.11 | -1.05 (-2.52, 0.44) |  | 1.06 | 1.36 | 3.56 (2.16, 4.99) * |
| China | 57.32 | 42.93 | -3.95 (-4.32, -3.57) * |  | 54.56 | 41.00 | -3.92 (-4.19, -3.66) * |  | 2.59 | 1.77 | -4.70 (-7.36, -1.96) * |  | 17.11 | 16.12 | -0.32 (-2.91, 2.33) |
| Colombia | 21.31 | 18.65 | -1.93 (-2.49, -1.38) * |  | 20.47 | 17.79 | -2.03 (-2.57, -1.49) * |  | 0.79 | 0.79 | -0.01 (-0.94, 0.92) |  | 4.56 | 6.27 | 4.56 (3.42, 5.70) * |
| Comoros | 290.50 | 270.23 | -0.89 (-1.82, 0.05) |  | 277.38 | 243.42 | -1.86 (-2.66, -1.07) * |  | 13.05 | 26.63 | 12.48 (7.79, 17.37) * |  | 6.35 | 17.18 | 16.94 (11.96, 22.15) * |
| Congo | 256.26 | 232.56 | -1.14 (-1.52, -0.76) * |  | 251.93 | 227.79 | -1.18 (-1.58, -0.79) * |  | 4.31 | 4.74 | 1.36 (0.51, 2.21) * |  | 2.07 | 3.06 | 5.68 (4.64, 6.74) * |
| Costa Rica | 9.87 | 7.61 | -3.59 (-5.13, -2.02) * |  | 9.68 | 7.45 | -3.62 (-5.16, -2.06) * |  | 0.18 | 0.15 | -2.22 (-4.16, -0.24) * |  | 1.02 | 1.18 | 2.22 (0.35, 4.13) * |
| Croatia | 9.83 | 7.04 | -4.36 (-5.78, -2.91) * |  | 9.77 | 7.01 | -4.34 (-5.75, -2.91) * |  | 0.05 | 0.03 | -8.59 (-12.47, -4.54) * |  | 0.80 | 0.56 | -3.97 (-7.94, 0.17) |
| Cuba | 6.70 | 5.11 | -3.85 (-5.34, -2.34) * |  | 6.59 | 4.98 | -3.99 (-5.49, -2.46) * |  | 0.10 | 0.12 | 2.32 (2.15, 2.50) * |  | 0.58 | 0.94 | 7.05 (6.95, 7.15) * |
| Cyprus | 3.30 | 2.72 | -3.27 (-4.88, -1.63) * |  | 3.10 | 2.66 | -2.74 (-4.23, -1.23) * |  | 0.18 | 0.05 | -16.76 (-20.67, -12.66) * |  | 1.59 | 0.65 | -12.12 (-15.79, -8.29) * |
| Czechia | 5.03 | 4.01 | -3.31 (-4.69, -1.91) * |  | 4.90 | 3.92 | -3.31 (-4.68, -1.92) * |  | 0.11 | 0.08 | -4.28 (-5.89, -2.64) * |  | 1.63 | 1.74 | 0.93 (-0.43, 2.30) |
| Cote d'Ivoire | 198.61 | 169.76 | -1.86 (-2.64, -1.08) * |  | 192.73 | 164.24 | -1.91 (-2.70, -1.11) * |  | 5.85 | 5.48 | -0.39 (-1.18, 0.40) |  | 2.79 | 3.54 | 3.93 (3.14, 4.73) * |
| Democratic Republic of the Congo | 342.31 | 312.44 | -1.09 (-1.43, -0.75) * |  | 335.62 | 304.86 | -1.16 (-1.51, -0.80) * |  | 6.66 | 7.54 | 1.73 (1.02, 2.45) * |  | 3.19 | 4.86 | 6.10 (5.19, 7.02) * |
| Denmark | 5.83 | 4.74 | -2.79 (-3.49, -2.09) * |  | 5.73 | 4.65 | -2.84 (-3.58, -2.10) * |  | 0.09 | 0.08 | -0.65 (-2.08, 0.80) |  | 0.80 | 1.07 | 3.81 (2.26, 5.38) * |
| Djibouti | 299.39 | 235.69 | -2.98 (-3.56, -2.40) * |  | 284.84 | 220.11 | -3.21 (-3.80, -2.62) * |  | 14.48 | 15.49 | 0.92 (-0.38, 2.23) |  | 6.96 | 9.99 | 5.19 (3.70, 6.70) * |
| Dominica | 25.55 | 23.92 | -0.64 (-1.14, -0.14) * |  | 25.39 | 23.75 | -0.66 (-1.17, -0.14) * |  | 0.14 | 0.16 | 1.60 (-0.50, 3.74) |  | 0.81 | 1.29 | 6.54 (3.91, 9.23) * |
| Dominican Republic | 42.75 | 41.87 | 0.25 (-1.85, 2.39) |  | 42.59 | 41.63 | 0.22 (-1.87, 2.36) |  | 0.16 | 0.22 | 5.15 (1.45, 8.98) * |  | 0.89 | 1.76 | 10.18 (5.90, 14.63) * |
| Ecuador | 40.83 | 34.80 | -2.17 (-3.31, -1.02) * |  | 38.62 | 32.90 | -2.17 (-3.3, -1.03) * |  | 2.08 | 1.76 | -2.38 (-3.75, -1.00) * |  | 11.95 | 13.91 | 2.13 (0.90, 3.37) * |
| Egypt | 16.37 | 13.61 | -2.78 (-3.11, -2.45) * |  | 15.52 | 12.96 | -2.72 (-3.04, -2.40) * |  | 0.82 | 0.62 | -3.99 (-5.08, -2.88) * |  | 2.07 | 2.19 | 0.75 (0.06, 1.45) * |
| El Salvador | 13.87 | 12.17 | -1.87 (-2.06, -1.68) * |  | 13.60 | 11.93 | -1.87 (-2.07, -1.67) * |  | 0.26 | 0.22 | -2.03 (-2.40, -1.66) * |  | 1.46 | 1.74 | 2.59 (2.49, 2.69) * |
| Equatorial Guinea | 183.89 | 159.40 | -1.95 (-2.47, -1.43) * |  | 180.31 | 155.33 | -2.04 (-2.57, -1.49) * |  | 3.57 | 4.04 | 1.62 (1.13, 2.11) * |  | 1.71 | 2.61 | 5.96 (5.32, 6.61) * |
| Estonia | 17.96 | 12.73 | -4.62 (-6.89, -2.29) * |  | 13.02 | 10.00 | -3.42 (-5.46, -1.35) * |  | 4.31 | 2.23 | -9.08 (-11.99, -6.08) * |  | 63.00 | 48.94 | -3.65 (-6.15, -1.08) * |
| Eswatini | 327.80 | 319.58 | 0.11 (-0.61, 0.83) |  | 293.86 | 285.50 | 0.03 (-0.70, 0.77) |  | 33.78 | 33.86 | 0.75 (-0.52, 2.04) |  | 15.87 | 21.85 | 5.36 (4.25, 6.49) * |
| Ethiopia | 289.83 | 254.39 | -1.94 (-2.8, -1.08) * |  | 282.83 | 246.23 | -2.05 (-2.94, -1.16) * |  | 6.96 | 8.11 | 1.85 (1.23, 2.48) * |  | 3.34 | 5.23 | 6.22 (5.43, 7.01) * |
| Finland | 5.29 | 4.30 | -2.80 (-4.15, -1.44) * |  | 5.15 | 4.14 | -2.95 (-4.35, -1.53) * |  | 0.13 | 0.14 | 1.14 (0.56, 1.73) * |  | 1.19 | 1.78 | 5.84 (5.12, 6.57) * |
| France | 6.96 | 6.44 | -1.19 (-1.47, -0.91) * |  | 6.83 | 6.30 | -1.23 (-1.51, -0.94) * |  | 0.12 | 0.12 | 0.17 (-0.05, 0.39) |  | 1.09 | 1.54 | 4.89 (4.66, 5.12) * |
| Gabon | 273.53 | 239.00 | -1.78 (-1.98, -1.57) * |  | 266.34 | 231.62 | -1.84 (-2.06, -1.62) * |  | 7.16 | 7.33 | 0.26 (-0.35, 0.88) |  | 3.42 | 4.73 | 4.60 (3.76, 5.44) * |
| Gambia | 299.19 | 237.50 | -3.27 (-3.35, -3.18) * |  | 291.62 | 232.10 | -3.24 (-3.31, -3.17) * |  | 7.53 | 5.36 | -4.59 (-5.58, -3.59) * |  | 3.50 | 3.46 | -0.09 (-0.70, 0.52) |
| Georgia | 68.08 | 54.97 | -2.77 (-3.11, -2.42) * |  | 55.18 | 42.83 | -3.28 (-3.71, -2.85) * |  | 11.18 | 9.96 | -1.53 (-2.08, -0.97) * |  | 171.58 | 218.27 | 3.51 (2.74, 4.28) * |
| Germany | 5.70 | 6.16 | 0.76 (-0.83, 2.38) |  | 5.52 | 5.89 | 0.58 (-0.95, 2.14) |  | 0.16 | 0.24 | 5.05 (1.59, 8.62) * |  | 1.46 | 2.96 | 9.82 (6.15, 13.62) * |
| Ghana | 240.77 | 199.14 | -2.14 (-3.46, -0.81) * |  | 234.74 | 194.01 | -2.16 (-3.49, -0.81) * |  | 6.00 | 5.10 | -1.67 (-2.62, -0.71) * |  | 2.83 | 3.29 | 2.78 (1.67, 3.91) * |
| Greece | 4.48 | 3.86 | -1.73 (-2.99, -0.45) * |  | 4.24 | 3.73 | -1.46 (-2.66, -0.24) * |  | 0.22 | 0.12 | -8.21 (-10.37, -6.00) * |  | 1.86 | 1.46 | -3.01 (-4.67, -1.31) * |
| Grenada | 13.88 | 11.17 | -3.65 (-5.02, -2.27) * |  | 13.81 | 11.07 | -3.70 (-5.08, -2.31) * |  | 0.07 | 0.09 | 3.45 (1.35, 5.59) * |  | 0.37 | 0.69 | 8.36 (5.82, 10.97) * |
| Guatemala | 19.27 | 16.81 | -1.87 (-1.99, -1.75) * |  | 18.74 | 16.35 | -1.87 (-1.99, -1.74) * |  | 0.50 | 0.42 | -2.32 (-2.61, -2.02) * |  | 2.85 | 3.30 | 2.22 (2.09, 2.35) * |
| Guinea | 206.34 | 180.75 | -1.64 (-2.51, -0.76) * |  | 201.40 | 176.83 | -1.61 (-2.51, -0.71) * |  | 4.91 | 3.89 | -2.82 (-3.66, -1.97) * |  | 2.29 | 2.51 | 1.69 (1.10, 2.27) * |
| Guinea-Bissau | 176.45 | 155.58 | -1.48 (-2.29, -0.67) * |  | 172.63 | 152.75 | -1.44 (-2.27, -0.61) * |  | 3.80 | 2.81 | -3.54 (-4.87, -2.18) * |  | 1.79 | 1.81 | 0.82 (-0.28, 1.93) |
| Guyana | 48.73 | 41.98 | -2.03 (-3.03, -1.02) * |  | 48.43 | 41.63 | -2.05 (-3.03, -1.06) * |  | 0.28 | 0.33 | 1.29 (-2.73, 5.47) |  | 1.65 | 2.59 | 5.60 (1.29, 10.10) * |
| Haiti | 79.48 | 73.77 | -1.1 (-1.42, -0.78) * |  | 79.23 | 73.45 | -1.12 (-1.43, -0.80) * |  | 0.24 | 0.30 | 2.89 (0.69, 5.14) * |  | 1.33 | 2.34 | 7.86 (5.16, 10.63) * |
| Honduras | 34.32 | 31.64 | -0.93 (-1.43, -0.42) * |  | 33.45 | 30.79 | -0.95 (-1.45, -0.44) * |  | 0.82 | 0.78 | -0.41 (-0.91, 0.11) |  | 4.68 | 6.17 | 4.19 (3.48, 4.91) * |
| Hungary | 8.06 | 5.22 | -5.68 (-7.59, -3.73) * |  | 7.78 | 5.14 | -5.45 (-7.24, -3.63) * |  | 0.24 | 0.07 | -15.49 (-21.36, -9.19) * |  | 3.47 | 1.54 | -10.09 (-15.72, -4.09) * |
| Iceland | 6.24 | 5.32 | -2.17 (-3.09, -1.24) * |  | 5.98 | 5.27 | -1.75 (-2.52, -0.97) * |  | 0.24 | 0.05 | -19.44 (-23.12, -15.59) * |  | 2.05 | 0.66 | -14.86 (-18.28, -11.3) * |
| India | 256.39 | 223.01 | -2.12 (-2.76, -1.47) * |  | 239.92 | 206.53 | -2.27 (-2.92, -1.62) * |  | 16.18 | 16.09 | -0.10 (-0.79, 0.59) |  | 29.12 | 39.44 | 4.32 (3.80, 4.84) * |
| Indonesia | 181.00 | 137.38 | -3.68 (-4.43, -2.92) * |  | 179.80 | 136.81 | -3.65 (-4.38, -2.92) * |  | 1.13 | 0.53 | -9.46 (-13.90, -4.79) * |  | 7.44 | 4.81 | -5.22 (-9.69, -0.52) * |
| Iran | 14.23 | 12.29 | -2.33 (-2.94, -1.72) * |  | 13.99 | 12.07 | -2.36 (-2.95, -1.75) * |  | 0.23 | 0.22 | -1.10 (-2.08, -0.12) * |  | 0.59 | 0.76 | 3.34 (2.45, 4.24) * |
| Iraq | 43.99 | 34.02 | -3.27 (-3.77, -2.77) * |  | 42.88 | 33.01 | -3.33 (-3.84, -2.82) * |  | 1.08 | 0.98 | -1.12 (-1.56, -0.68) * |  | 2.80 | 3.46 | 3.31 (2.88, 3.75) * |
| Ireland | 6.74 | 5.49 | -2.83 (-4.10, -1.55) * |  | 6.61 | 5.40 | -2.78 (-4.05, -1.50) * |  | 0.12 | 0.08 | -6.15 (-7.56, -4.71) * |  | 1.05 | 0.96 | -1.33 (-2.48, -0.17) * |
| Israel | 4.02 | 3.10 | -3.46 (-4.98, -1.92) * |  | 3.79 | 2.91 | -3.56 (-5.16, -1.94) * |  | 0.21 | 0.17 | -2.38 (-2.70, -2.06) * |  | 1.84 | 2.18 | 2.49 (2.45, 2.53) * |
| Italy | 8.97 | 7.78 | -2.26 (-3.94, -0.55) * |  | 8.63 | 7.53 | -2.17 (-3.84, -0.46) * |  | 0.31 | 0.22 | -5.21 (-7.00, -3.38) * |  | 2.84 | 2.75 | -0.63 (-2.30, 1.07) |
| Jamaica | 4.69 | 3.71 | -3.43 (-4.71, -2.12) * |  | 4.64 | 3.65 | -3.49 (-4.81, -2.15) * |  | 0.05 | 0.06 | 0.69 (-1.23, 2.64) |  | 0.30 | 0.45 | 4.95 (2.88, 7.07) * |
| Japan | 8.39 | 6.69 | -3.08 (-3.49, -2.68) * |  | 8.33 | 6.63 | -3.10 (-3.50, -2.70) * |  | 0.06 | 0.06 | -1.49 (-2.43, -0.53) * |  | 0.56 | 0.69 | 3.36 (2.09, 4.64) * |
| Jordan | 6.00 | 4.83 | -2.80 (-3.48, -2.11) * |  | 5.67 | 4.63 | -2.63 (-3.25, -2.00) * |  | 0.32 | 0.20 | -6.24 (-8.08, -4.36) * |  | 0.83 | 0.69 | -2.16 (-3.95, -0.33) * |
| Kazakhstan | 89.86 | 57.80 | -5.80 (-7.11, -4.47) * |  | 56.45 | 43.28 | -3.28 (-4.25, -2.30) * |  | 29.15 | 11.91 | -12.08 (-13.94, -10.17) * |  | 425.62 | 260.95 | -6.79 (-8.21, -5.35) * |
| Kenya | 282.89 | 269.26 | -0.65 (-0.87, -0.43) * |  | 278.57 | 265.02 | -0.66 (-0.84, -0.47) * |  | 4.30 | 4.21 | -0.10 (-2.68, 2.54) |  | 2.05 | 2.71 | 4.22 (1.73, 6.76) * |
| Kiribati | 217.84 | 219.67 | 0.46 (-0.40, 1.31) |  | 217.22 | 218.98 | 0.45 (-0.40, 1.31) |  | 0.59 | 0.63 | 1.08 (-0.42, 2.60) |  | 3.77 | 5.73 | 6.18 (4.10, 8.30) * |
| Kuwait | 22.68 | 17.37 | -3.61 (-4.7, -2.52) * |  | 22.32 | 17.08 | -3.62 (-4.73, -2.49) * |  | 0.35 | 0.28 | -3.48 (-4.21, -2.75) * |  | 0.90 | 0.98 | 0.82 (-0.12, 1.77) |
| Kyrgyzstan | 99.65 | 86.67 | -2.04 (-3.39, -0.67) * |  | 67.90 | 59.76 | -1.93 (-3.33, -0.50) * |  | 27.52 | 22.08 | -3.03 (-4.29, -1.75) * |  | 423.44 | 483.71 | 1.90 (0.40, 3.42) * |
| Laos | 135.47 | 121.14 | -1.36 (-1.83, -0.90) * |  | 134.50 | 120.52 | -1.34 (-1.78, -0.89) * |  | 0.91 | 0.57 | -5.58 (-9.45, -1.53) * |  | 6.05 | 5.19 | -1.32 (-5.21, 2.74) |
| Latvia | 30.74 | 22.62 | -4.09 (-4.79, -3.38) * |  | 26.71 | 19.98 | -3.86 (-4.37, -3.34) * |  | 3.50 | 2.16 | -6.46 (-8.56, -4.32) * |  | 53.65 | 47.35 | -1.58 (-3.61, 0.48) |
| Lebanon | 14.82 | 12.01 | -3.13 (-3.83, -2.43) * |  | 14.58 | 11.78 | -3.18 (-3.88, -2.46) * |  | 0.23 | 0.22 | -0.93 (-1.40, -0.46) * |  | 0.59 | 0.78 | 3.56 (2.84, 4.30) * |
| Lesotho | 415.62 | 427.82 | 0.80 (-0.06, 1.66) |  | 399.42 | 408.60 | 0.71 (-0.19, 1.62) |  | 16.12 | 19.10 | 2.88 (1.87, 3.91) * |  | 7.66 | 12.32 | 7.41 (6.54, 8.30) * |
| Liberia | 202.14 | 168.17 | -2.43 (-2.81, -2.04) * |  | 198.12 | 164.89 | -2.43 (-2.83, -2.03) * |  | 4.00 | 3.26 | -2.40 (-3.39, -1.40) * |  | 1.88 | 2.10 | 1.93 (1.22, 2.65) * |
| Libya | 16.19 | 15.11 | -0.75 (-1.09, -0.40) * |  | 15.87 | 14.80 | -0.75 (-1.10, -0.40) * |  | 0.31 | 0.29 | -0.54 (-1.49, 0.41) |  | 0.78 | 1.04 | 4.17 (2.74, 5.61) * |
| Lithuania | 44.55 | 34.63 | -3.36 (-3.90, -2.82) * |  | 35.79 | 27.43 | -3.55 (-4.06, -3.04) * |  | 7.61 | 5.91 | -3.40 (-4.07, -2.72) * |  | 115.89 | 129.46 | 1.67 (1.22, 2.13) * |
| Luxembourg | 6.74 | 6.14 | -1.58 (-2.26, -0.90) * |  | 6.44 | 5.96 | -1.36 (-2.05, -0.66) * |  | 0.27 | 0.16 | -7.72 (-8.54, -6.88) * |  | 2.37 | 2.00 | -2.86 (-4.05, -1.66) * |
| Madagascar | 419.12 | 355.80 | -2.14 (-2.39, -1.88) * |  | 412.31 | 346.31 | -2.28 (-2.55, -2.00) * |  | 6.78 | 9.43 | 4.41 (2.20, 6.68) * |  | 3.28 | 6.09 | 8.80 (6.42, 11.24) * |
| Malawi | 284.49 | 248.29 | -1.82 (-2.18, -1.46) * |  | 281.31 | 243.24 | -1.95 (-2.32, -1.59) * |  | 3.17 | 5.02 | 6.83 (6.37, 7.29) * |  | 1.49 | 3.24 | 11.66 (10.84, 12.47) * |
| Malaysia | 96.67 | 96.97 | 0.23 (-0.82, 1.29) |  | 95.72 | 96.09 | 0.24 (-0.79, 1.28) |  | 0.89 | 0.80 | -0.93 (-4.00, 2.23) |  | 5.92 | 7.33 | 3.48 (0.41, 6.65) * |
| Maldives | 34.61 | 31.42 | -1.69 (-2.99, -0.38) * |  | 34.35 | 31.27 | -1.66 (-2.94, -0.36) * |  | 0.24 | 0.13 | -7.66 (-12.30, -2.78) * |  | 1.59 | 1.22 | -3.30 (-7.95, 1.58) |
| Mali | 195.91 | 161.62 | -2.41 (-2.9, -1.92) * |  | 190.62 | 157.27 | -2.41 (-2.91, -1.91) * |  | 5.27 | 4.32 | -2.42 (-3.18, -1.65) * |  | 2.47 | 2.79 | 2.02 (1.54, 2.51) * |
| Malta | 8.68 | 8.82 | -0.26 (-1.19, 0.69) |  | 8.47 | 8.74 | -0.05 (-1.04, 0.95) |  | 0.19 | 0.07 | -13.98 (-15.38, -12.55) * |  | 1.70 | 0.86 | -9.53 (-10.73, -8.32) * |
| Marshall Islands | 136.55 | 115.07 | -2.40 (-2.54, -2.27) * |  | 133.72 | 113.76 | -2.29 (-2.36, -2.21) * |  | 2.67 | 1.20 | -10.21 (-13.87, -6.40) * |  | 16.62 | 10.90 | -5.18 (-8.42, -1.82) * |
| Mauritania | 116.85 | 86.99 | -4.03 (-4.18, -3.89) * |  | 113.98 | 85.00 | -4.01 (-4.16, -3.86) * |  | 2.86 | 1.98 | -4.88 (-6.06, -3.69) * |  | 1.35 | 1.27 | -0.60 (-1.55, 0.35) |
| Mauritius | 12.28 | 10.55 | -2.26 (-4.55, 0.10) |  | 12.15 | 10.49 | -2.18 (-4.46, 0.15) |  | 0.12 | 0.05 | -11.53 (-15.80, -7.05) * |  | 0.77 | 0.47 | -6.55 (-10.45, -2.48) * |
| Mexico | 15.35 | 15.77 | 1.04 (-0.05, 2.16) |  | 14.92 | 15.25 | 0.97 (-0.12, 2.08) |  | 0.41 | 0.48 | 3.04 (1.66, 4.44) * |  | 2.30 | 3.80 | 8.11 (6.87, 9.37) * |
| Micronesia (Federated States of) | 67.64 | 63.07 | -0.87 (-1.06, -0.68) * |  | 67.43 | 62.85 | -0.88 (-1.07, -0.68) * |  | 0.20 | 0.20 | -0.01 (-1.42, 1.41) |  | 1.26 | 1.78 | 4.89 (3.01, 6.79) * |
| Mongolia | 105.76 | 91.85 | -1.36 (-2.52, -0.19) * |  | 96.17 | 80.98 | -1.77 (-2.88, -0.65) * |  | 8.31 | 8.92 | 1.40 (-0.64, 3.48) |  | 127.90 | 195.42 | 6.55 (4.19, 8.97) * |
| Montenegro | 6.94 | 5.75 | -2.50 (-2.78, -2.22) * |  | 6.83 | 5.69 | -2.46 (-2.75, -2.16) * |  | 0.09 | 0.05 | -6.48 (-8.42, -4.50) * |  | 1.38 | 1.17 | -1.58 (-3.80, 0.69) |
| Morocco | 94.86 | 79.51 | -1.85 (-3.00, -0.70) * |  | 93.23 | 77.85 | -1.90 (-3.06, -0.73) * |  | 1.59 | 1.60 | 0.58 (-0.16, 1.32) |  | 4.05 | 5.65 | 5.27 (4.36, 6.19) * |
| Mozambique | 353.17 | 308.38 | -1.35 (-2.36, -0.33) * |  | 338.12 | 292.55 | -1.47 (-2.48, -0.44) * |  | 14.98 | 15.73 | 1.14 (0.21, 2.08) * |  | 7.15 | 10.15 | 5.51 (4.40, 6.63) * |
| Namibia | 361.48 | 312.21 | -2.05 (-2.43, -1.67) * |  | 342.33 | 293.40 | -2.15 (-2.48, -1.83) * |  | 19.06 | 18.70 | -0.31 (-1.67, 1.07) |  | 9.03 | 12.06 | 4.09 (2.91, 5.28) * |
| Nauru | 73.80 | 62.42 | -2.14 (-2.66, -1.61) * |  | 73.59 | 62.22 | -2.14 (-2.66, -1.62) * |  | 0.20 | 0.18 | -1.05 (-1.98, -0.11) * |  | 1.27 | 1.66 | 3.86 (2.46, 5.29) * |
| Nepal | 212.71 | 168.14 | -3.10 (-3.39, -2.80) * |  | 204.46 | 160.04 | -3.23 (-3.52, -2.94) * |  | 8.10 | 7.91 | -0.11 (-0.66, 0.44) |  | 14.36 | 19.38 | 4.59 (4.25, 4.94) * |
| Netherlands | 5.35 | 4.62 | -1.97 (-2.65, -1.30) * |  | 5.23 | 4.52 | -1.95 (-2.63, -1.26) * |  | 0.11 | 0.09 | -3.51 (-3.92, -3.09) * |  | 1.01 | 1.13 | 1.33 (0.98, 1.68) * |
| Nicaragua | 28.13 | 23.61 | -2.38 (-2.50, -2.25) * |  | 27.65 | 23.14 | -2.41 (-2.54, -2.29) * |  | 0.46 | 0.43 | -0.56 (-1.15, 0.03) |  | 2.61 | 3.42 | 4.04 (3.61, 4.46) * |
| Niger | 181.73 | 161.68 | -1.48 (-1.97, -0.98) * |  | 176.95 | 157.79 | -1.46 (-1.96, -0.94) * |  | 4.76 | 3.86 | -2.50 (-3.22, -1.78) * |  | 2.26 | 2.49 | 1.73 (1.15, 2.32) * |
| Nigeria | 228.34 | 183.02 | -2.95 (-3.49, -2.41) * |  | 219.06 | 175.30 | -2.98 (-3.55, -2.40) * |  | 9.24 | 7.68 | -2.31 (-2.99, -1.62) * |  | 4.33 | 4.95 | 2.17 (1.74, 2.61) * |
| North Macedonia | 12.27 | 10.46 | -2.15 (-2.36, -1.94) * |  | 11.97 | 10.29 | -2.03 (-2.23, -1.83) * |  | 0.27 | 0.14 | -8.62 (-9.05, -8.18) * |  | 3.96 | 3.06 | -3.46 (-3.70, -3.23) * |
| Norway | 7.76 | 5.32 | -5.25 (-6.37, -4.12) * |  | 7.52 | 5.17 | -5.23 (-6.34, -4.10) * |  | 0.22 | 0.14 | -6.52 (-7.86, -5.15) * |  | 1.92 | 1.74 | -1.42 (-2.29, -0.53) * |
| Oman | 20.28 | 14.75 | -4.54 (-5.34, -3.74) * |  | 19.90 | 14.47 | -4.54 (-5.38, -3.70) * |  | 0.37 | 0.28 | -4.43 (-6.01, -2.82) * |  | 0.94 | 0.98 | -0.06 (-1.98, 1.90) |
| Pakistan | 311.26 | 256.88 | -2.61 (-2.98, -2.24) * |  | 297.44 | 242.45 | -2.78 (-3.17, -2.40) * |  | 13.58 | 14.09 | 0.60 (0.41, 0.80) * |  | 24.47 | 34.53 | 5.06 (4.67, 5.46) * |
| Palau | 41.40 | 39.92 | -0.36 (-0.60, -0.13) * |  | 41.27 | 39.78 | -0.37 (-0.60, -0.13) * |  | 0.12 | 0.12 | 0.66 (-0.24, 1.57) |  | 0.75 | 1.11 | 5.66 (4.25, 7.09) * |
| Panama | 36.02 | 32.40 | -1.58 (-2.36, -0.78) * |  | 35.11 | 31.59 | -1.58 (-2.37, -0.77) * |  | 0.85 | 0.75 | -1.86 (-2.16, -1.56) * |  | 4.86 | 5.91 | 2.73 (2.14, 3.32) * |
| Papua New Guinea | 133.79 | 122.41 | -1.38 (-1.67, -1.09) * |  | 130.55 | 117.32 | -1.60 (-2.01, -1.19) * |  | 3.02 | 4.66 | 4.76 (-0.66, 10.48) |  | 21.00 | 42.50 | 8.86 (3.44, 14.56) * |
| Paraguay | 34.53 | 30.48 | -1.56 (-1.95, -1.17) * |  | 33.63 | 29.38 | -1.70 (-2.09, -1.32) * |  | 0.85 | 1.02 | 2.69 (1.77, 3.61) * |  | 4.83 | 8.04 | 7.63 (6.35, 8.92) * |
| Peru | 91.24 | 74.86 | -2.59 (-3.66, -1.50) * |  | 83.87 | 68.84 | -2.61 (-3.47, -1.75) * |  | 6.97 | 5.58 | -2.53 (-6.32, 1.41) |  | 40.13 | 44.04 | 1.81 (-1.99, 5.75) |
| Philippines | 228.97 | 322.36 | 5.12 (3.06, 7.22) * |  | 222.11 | 312.51 | 5.10 (3.02, 7.22) * |  | 6.43 | 9.02 | 5.39 (3.89, 6.92) * |  | 42.49 | 82.27 | 10.32 (8.56, 12.11) * |
| Poland | 15.49 | 12.02 | -3.48 (-4.26, -2.70) * |  | 15.34 | 11.90 | -3.49 (-4.26, -2.72) * |  | 0.13 | 0.10 | -3.64 (-5.49, -1.76) * |  | 1.95 | 2.10 | 1.17 (-0.64, 3.01) |
| Portugal | 14.13 | 11.62 | -2.59 (-3.15, -2.02) * |  | 13.90 | 11.43 | -2.59 (-3.14, -2.04) * |  | 0.21 | 0.17 | -2.64 (-4.27, -0.98) * |  | 1.88 | 2.12 | 1.99 (0.46, 3.55) * |
| Qatar | 17.26 | 12.99 | -4.04 (-4.70, -3.38) * |  | 16.97 | 12.72 | -4.10 (-4.76, -3.44) * |  | 0.28 | 0.26 | -0.97 (-1.47, -0.47) * |  | 0.71 | 0.92 | 3.61 (3.28, 3.93) * |
| Republic of Korea | 51.80 | 39.69 | -3.61 (-3.82, -3.39) * |  | 50.92 | 39.05 | -3.60 (-3.81, -3.39) * |  | 0.80 | 0.57 | -4.48 (-4.98, -3.97) * |  | 7.22 | 7.14 | 0.13 (-0.27, 0.54) |
| Republic of Moldova | 74.48 | 58.14 | -3.40 (-3.76, -3.04) * |  | 44.66 | 31.29 | -4.93 (-4.99, -4.87) * |  | 25.85 | 22.02 | -2.14 (-3.04, -1.24) * |  | 397.05 | 482.55 | 2.86 (1.74, 4.00) * |
| Romania | 46.57 | 39.11 | -2.28 (-3.60, -0.95) * |  | 44.67 | 37.53 | -2.27 (-3.52, -0.99) * |  | 1.64 | 1.29 | -3.39 (-6.03, -0.69) * |  | 25.71 | 28.34 | 1.16 (-1.61, 4.00) |
| Russian Federation | 101.35 | 71.76 | -4.79 (-7.61, -1.89) * |  | 68.70 | 48.90 | -4.70 (-7.52, -1.78) * |  | 28.45 | 18.75 | -5.80 (-8.62, -2.90) * |  | 420.25 | 410.82 | -0.38 (-2.84, 2.14) |
| Rwanda | 247.17 | 206.91 | -2.18 (-2.65, -1.70) * |  | 241.58 | 200.68 | -2.28 (-2.75, -1.81) * |  | 5.56 | 6.19 | 1.86 (0.63, 3.11) * |  | 2.66 | 3.99 | 6.23 (5.11, 7.36) * |
| Saint Kitts and Nevis | 23.42 | 20.17 | -1.60 (-2.80, -0.40) * |  | 23.32 | 20.05 | -1.63 (-2.81, -0.43) * |  | 0.09 | 0.11 | 2.40 (-0.68, 5.58) |  | 0.53 | 0.87 | 7.29 (3.68, 11.03) * |
| Saint Lucia | 14.16 | 12.26 | -1.94 (-2.11, -1.78) * |  | 14.10 | 12.20 | -1.96 (-2.12, -1.79) * |  | 0.05 | 0.06 | 0.26 (-0.94, 1.48) |  | 0.30 | 0.44 | 5.07 (3.38, 6.78) * |
| Saint Vincent and the Grenadines | 17.86 | 15.37 | -2.14 (-2.70, -1.58) * |  | 17.77 | 15.27 | -2.16 (-2.73, -1.59) * |  | 0.08 | 0.09 | 0.54 (-0.92, 2.02) |  | 0.48 | 0.72 | 5.26 (3.35, 7.21) * |
| Sao Tome and Principe | 100.94 | 77.10 | -3.75 (-3.83, -3.67) * |  | 97.44 | 74.29 | -3.76 (-3.83, -3.68) * |  | 3.49 | 2.78 | -3.58 (-4.81, -2.34) * |  | 1.68 | 1.80 | 0.44 (-0.98, 1.89) |
| Saudi Arabia | 56.53 | 42.53 | -3.28 (-4.27, -2.28) * |  | 55.08 | 41.31 | -3.33 (-4.32, -2.33) * |  | 1.42 | 1.19 | -1.68 (-2.88, -0.47) * |  | 3.59 | 4.19 | 2.97 (1.84, 4.11) * |
| Senegal | 185.95 | 158.02 | -2.09 (-2.83, -1.33) * |  | 182.16 | 154.46 | -2.12 (-2.90, -1.33) * |  | 3.77 | 3.53 | -0.56 (-1.90, 0.80) |  | 1.82 | 2.28 | 3.52 (2.23, 4.83) * |
| Serbia | 9.95 | 8.01 | -2.73 (-3.37, -2.09) * |  | 9.83 | 7.92 | -2.71 (-3.34, -2.09) * |  | 0.10 | 0.07 | -4.92 (-7.26, -2.52) * |  | 1.54 | 1.51 | 0.25 (-1.82, 2.37) |
| Seychelles | 29.75 | 28.05 | -1.00 (-1.66, -0.34) * |  | 29.66 | 27.99 | -0.99 (-1.65, -0.34) * |  | 0.08 | 0.06 | -3.69 (-6.87, -0.4) * |  | 0.56 | 0.58 | 0.67 (-2.52, 3.95) |
| Sierra Leone | 278.40 | 239.34 | -2.08 (-2.43, -1.73) * |  | 272.22 | 234.00 | -2.09 (-2.42, -1.75) * |  | 6.15 | 5.30 | -1.94 (-2.8, -1.07) * |  | 2.91 | 3.42 | 2.40 (1.77, 3.03) * |
| Singapore | 32.82 | 29.50 | -1.60 (-3.00, -0.19) * |  | 32.35 | 29.13 | -1.58 (-2.97, -0.16) * |  | 0.43 | 0.33 | -4.18 (-5.83, -2.51) * |  | 3.95 | 4.14 | 0.25 (-1.59, 2.11) |
| Slovakia | 5.27 | 4.13 | -3.60 (-4.50, -2.69) * |  | 5.21 | 4.09 | -3.56 (-4.45, -2.66) * |  | 0.05 | 0.03 | -7.92 (-9.98, -5.81) * |  | 0.81 | 0.64 | -3.15 (-5.14, -1.11) * |
| Slovenia | 6.10 | 4.46 | -4.29 (-5.90, -2.66) * |  | 6.08 | 4.45 | -4.26 (-5.86, -2.64) * |  | 0.02 | 0.00 | -15.95 (-20.01, -11.68) * |  | 0.26 | 0.11 | -11.34 (-15.39, -7.10) * |
| Solomon Islands | 47.08 | 44.32 | -0.63 (-0.96, -0.31) * |  | 46.94 | 44.17 | -0.64 (-0.97, -0.31) * |  | 0.13 | 0.14 | 0.59 (-0.41, 1.60) |  | 0.85 | 1.25 | 5.66 (4.14, 7.20) * |
| South Africa | 356.19 | 372.42 | 0.54 (0.01, 1.08) * |  | 346.54 | 362.37 | 0.54 (0.06, 1.01) * |  | 9.60 | 9.98 | 0.66 (-2.25, 3.65) |  | 4.57 | 6.44 | 5.07 (2.24, 7.97) * |
| Spain | 8.26 | 6.77 | -2.86 (-3.16, -2.56) * |  | 8.13 | 6.68 | -2.83 (-3.12, -2.53) * |  | 0.12 | 0.08 | -5.54 (-6.11, -4.97) * |  | 1.08 | 1.02 | -0.90 (-1.29, -0.50) * |
| Sri Lanka | 39.67 | 38.19 | -0.77 (-1.48, -0.05) * |  | 39.48 | 38.04 | -0.76 (-1.46, -0.05) * |  | 0.18 | 0.14 | -3.22 (-5.62, -0.75) * |  | 1.16 | 1.30 | 1.76 (-0.25, 3.82) |
| Sudan | 39.77 | 31.49 | -3.10 (-3.37, -2.82) * |  | 38.95 | 30.83 | -3.10 (-3.37, -2.82) * |  | 0.80 | 0.64 | -3.15 (-4.06, -2.22) * |  | 2.07 | 2.27 | 1.23 (0.01, 2.46) * |
| Suriname | 12.12 | 10.89 | -1.32 (-1.59, -1.05) * |  | 12.07 | 10.84 | -1.33 (-1.6, -1.06) * |  | 0.04 | 0.04 | 0.48 (-1.13, 2.11) |  | 0.23 | 0.34 | 5.28 (3.18, 7.43) * |
| Sweden | 8.74 | 8.75 | -0.03 (-0.46, 0.40) |  | 8.41 | 8.44 | 0.02 (-0.40, 0.44) |  | 0.31 | 0.27 | -1.73 (-2.30, -1.16) * |  | 2.73 | 3.44 | 3.18 (2.84, 3.53) * |
| Switzerland | 6.39 | 5.91 | -1.18 (-1.36, -0.99) * |  | 6.20 | 5.64 | -1.39 (-1.51, -1.27) * |  | 0.18 | 0.24 | 3.81 (1.14, 6.54) * |  | 1.64 | 3.01 | 8.43 (5.67, 11.26) * |
| Syrian Arab Republic | 10.92 | 9.41 | -2.14 (-2.33, -1.95) * |  | 10.60 | 9.15 | -2.10 (-2.30, -1.90) * |  | 0.32 | 0.25 | -3.55 (-3.78, -3.32) * |  | 0.83 | 0.88 | 0.72 (0.32, 1.12) * |
| Tajikistan | 60.37 | 53.52 | -1.28 (-2.01, -0.54) * |  | 50.85 | 44.24 | -1.50 (-2.24, -0.75) * |  | 8.25 | 7.61 | -0.92 (-1.81, -0.03) * |  | 127.67 | 166.74 | 4.02 (2.97, 5.08) * |
| Thailand | 118.63 | 113.74 | -0.43 (-1.28, 0.43) |  | 115.46 | 110.74 | -0.43 (-1.25, 0.39) |  | 2.98 | 2.75 | -0.69 (-2.85, 1.51) |  | 19.10 | 25.06 | 4.34 (2.53, 6.18) * |
| Timor-Leste | 122.54 | 125.60 | 0.35 (0.29, 0.42) * |  | 121.59 | 124.94 | 0.38 (0.30, 0.46) * |  | 0.89 | 0.61 | -4.67 (-7.28, -1.99) * |  | 5.90 | 5.55 | -0.36 (-2.91, 2.26) |
| Togo | 215.24 | 178.53 | -2.24 (-2.94, -1.54) * |  | 209.60 | 174.15 | -2.22 (-2.93, -1.51) * |  | 5.62 | 4.36 | -2.93 (-4.27, -1.56) * |  | 2.61 | 2.81 | 1.57 (0.57, 2.57) * |
| Trinidad and Tobago | 11.22 | 9.85 | -1.99 (-2.27, -1.70) * |  | 11.17 | 9.79 | -2.00 (-2.28, -1.71) * |  | 0.05 | 0.06 | 0.16 (-1.73, 2.09) |  | 0.30 | 0.44 | 4.71 (2.40, 7.08) * |
| Tunisia | 17.39 | 14.03 | -2.98 (-3.33, -2.63) * |  | 17.19 | 13.84 | -3.01 (-3.35, -2.66) * |  | 0.19 | 0.18 | -1.06 (-1.82, -0.29) * |  | 0.50 | 0.64 | 3.34 (2.76, 3.93) * |
| Turkey | 21.88 | 16.96 | -3.52 (-3.92, -3.12) * |  | 21.08 | 16.29 | -3.55 (-3.98, -3.12) * |  | 0.78 | 0.64 | -2.89 (-3.44, -2.34) * |  | 2.02 | 2.27 | 1.34 (0.61, 2.08) * |
| Turkmenistan | 57.08 | 47.27 | -2.23 (-2.91, -1.54) * |  | 47.11 | 37.90 | -2.58 (-3.26, -1.90) * |  | 8.63 | 7.68 | -1.44 (-2.70, -0.16) * |  | 134.10 | 168.34 | 3.41 (2.01, 4.84) * |
| Uganda | 213.13 | 209.84 | 0.03 (-0.36, 0.42) |  | 208.06 | 202.92 | -0.11 (-0.50, 0.29) |  | 5.04 | 6.87 | 4.71 (4.43, 4.99) * |  | 2.40 | 4.43 | 9.32 (9.00, 9.65) * |
| Ukraine | 82.50 | 78.05 | -0.45 (-1.70, 0.82) |  | 57.54 | 49.96 | -1.50 (-2.24, -0.75) * |  | 21.59 | 23.04 | 0.90 (-2.06, 3.94) |  | 337.36 | 504.89 | 5.80 (2.62, 9.09) * |
| United Arab Emirates | 27.25 | 21.88 | -3.19 (-3.95, -2.42) * |  | 26.76 | 21.42 | -3.23 (-4.01, -2.44) * |  | 0.48 | 0.44 | -1.33 (-1.84, -0.82) * |  | 1.23 | 1.57 | 3.17 (2.37, 3.98) * |
| United Kingdom | 15.34 | 9.78 | -5.63 (-6.49, -4.77) * |  | 15.13 | 9.65 | -5.62 (-6.48, -4.75) * |  | 0.20 | 0.12 | -6.89 (-7.28, -6.50) * |  | 1.73 | 1.50 | -1.85 (-2.58, -1.11) * |
| United Republic of Tanzania | 285.33 | 246.15 | -1.95 (-2.29, -1.60) * |  | 280.54 | 239.96 | -2.06 (-2.39, -1.73) * |  | 4.77 | 6.15 | 3.55 (1.85, 5.28) * |  | 2.31 | 3.97 | 7.87 (6.00, 9.76) * |
| United States of America | 2.68 | 2.12 | -3.46 (-4.58, -2.32) * |  | 2.64 | 2.09 | -3.47 (-4.59, -2.34) * |  | 0.04 | 0.03 | -3.05 (-4.77, -1.30) * |  | 0.32 | 0.37 | 1.83 (0.34, 3.35) * |
| Uruguay | 17.49 | 17.60 | -0.20 (-2.11, 1.75) |  | 17.43 | 17.53 | -0.20 (-2.10, 1.74) |  | 0.05 | 0.06 | 0.20 (-3.06, 3.56) |  | 0.50 | 0.71 | 4.33 (1.05, 7.71) * |
| Uzbekistan | 68.64 | 55.00 | -3.06 (-3.22, -2.89) * |  | 46.42 | 38.95 | -2.39 (-2.56, -2.21) * |  | 19.32 | 13.17 | -5.33 (-5.49, -5.16) * |  | 289.67 | 288.46 | -0.14 (-0.38, 0.11) |
| Vanuatu | 68.15 | 66.53 | -0.28 (-0.40, -0.16) * |  | 68.04 | 66.40 | -0.29 (-0.40, -0.17) * |  | 0.10 | 0.12 | 1.83 (-0.54, 4.26) |  | 0.69 | 1.11 | 6.40 (3.80, 9.07) * |
| Viet Nam | 180.62 | 153.24 | -2.31 (-2.43, -2.20) * |  | 172.48 | 146.66 | -2.30 (-2.38, -2.21) * |  | 7.66 | 6.03 | -2.98 (-4.83, -1.08) * |  | 48.71 | 54.94 | 2.04 (0.61, 3.50) * |
| Yemen | 37.44 | 34.23 | -1.10 (-1.56, -0.63) * |  | 36.62 | 33.47 | -1.10 (-1.54, -0.66) * |  | 0.80 | 0.73 | -1.01 (-2.58, 0.58) |  | 2.06 | 2.57 | 3.46 (2.04, 4.90) * |
| Zambia | 297.39 | 247.97 | -2.43 (-2.92, -1.95) * |  | 291.22 | 238.59 | -2.67 (-3.20, -2.14) * |  | 6.15 | 9.31 | 5.81 (3.86, 7.80) * |  | 2.96 | 6.01 | 10.26 (8.08, 12.48) * |
| Zimbabwe | 295.77 | 254.84 | -2.03 (-2.15, -1.92) * |  | 281.55 | 241.72 | -2.08 (-2.20, -1.96) * |  | 14.16 | 13.03 | -1.12 (-1.21, -1.03) * |  | 6.69 | 8.41 | 3.32 (3.01, 3.64) * |

Countries were arranged in alphabetical order.

TB: tuberculosis; DS-TB: drug-susceptible tuberculosis; MDR-TB: multidrug-resistant tuberculosis; XDR-TB: extensively drug-resistant tuberculosis.

a: per 100 000 population; b: per 10 000 000 population; CI: confidence interval.

# Table S5: Estimated annual percentage change (EAPC, %) for prevalence of TB from 2012 to 2019

| Country name | Total TB | | |  | DS-TB | | |  | MDR-TB | | |  | XDR-TB | | |
| --- | --- | --- | --- | --- | --- | --- | --- | --- | --- | --- | --- | --- | --- | --- | --- |
|  | 2012^a^ | 2019^a^ | EAPC (95% CI) |  | 2012^b^ | 2019^b^ | EAPC (95% CI) |  | 2012^c^ | 2019^c^ | EAPC (95% CI) |  | 2012^d^ | 2019^d^ | EAPC (95% CI) |
| Afghanistan | 268.30 | 209.72 | -3.87 (-4.73, -3.00) * |  | 178.74 | 144.56 | -3.04 (-3.98, -2.08) * |  | 108.80 | 64.63 | -7.00 (-7.32, -6.68) * |  | 271.93 | 228.10 | -2.36 (-2.68, -2.04) * |
| Albania | 180.67 | 149.41 | -2.70 (-2.74, -2.65) * |  | 9.45 | 7.56 | -3.80 (-5.25, -2.32) * |  | 1.22 | 0.56 | -10.80 (-14.10, -7.37) * |  | 17.96 | 12.35 | -5.59 (-8.58, -2.49) * |
| Algeria | 114.82 | 92.04 | -3.08 (-3.18, -2.98) * |  | 37.03 | 27.06 | -4.49 (-5.16, -3.81) * |  | 6.41 | 5.55 | -2.27 (-2.93, -1.62) * |  | 16.72 | 19.60 | 1.95 (1.11, 2.80) * |
| Angola | 375.92 | 333.68 | -1.60 (-1.75, -1.44) * |  | 623.10 | 529.50 | -2.31 (-2.44, -2.17) * |  | 166.55 | 151.35 | -1.43 (-1.78, -1.08) * |  | 79.83 | 97.64 | 2.74 (2.18, 3.30) * |
| Antigua and Barbuda | 213.42 | 183.22 | -2.15 (-2.34, -1.97) * |  | 46.50 | 33.92 | -5.42 (-7.92, -2.85) * |  | 2.20 | 2.09 | -2.10 (-4.20, 0.05) |  | 12.56 | 16.54 | 2.33 (-0.06, 4.77) |
| Argentina | 191.81 | 167.66 | -1.92 (-1.97, -1.88) * |  | 19.48 | 17.46 | -1.73 (-2.02, -1.43) * |  | 3.14 | 2.47 | -3.17 (-4.05, -2.28) * |  | 28.21 | 31.07 | 1.54 (0.87, 2.22) * |
| Armenia | 210.35 | 174.69 | -2.62 (-2.68, -2.57) * |  | 55.15 | 37.56 | -5.35 (-7.20, -3.46) * |  | 102.61 | 64.61 | -6.38 (-8.46, -4.25) * |  | 1498.69 | 1415.68 | -0.84 (-2.44, 0.77) |
| Australia | 104.64 | 95.78 | -1.24 (-1.27, -1.20) * |  | 4.50 | 3.97 | -1.99 (-2.61, -1.38) * |  | 0.98 | 1.27 | 3.12 (1.95, 4.31) * |  | 8.93 | 15.95 | 7.94 (6.59, 9.31) * |
| Austria | 73.90 | 65.99 | -1.59 (-1.76, -1.43) * |  | 7.39 | 6.08 | -3.26 (-4.73, -1.77) * |  | 3.16 | 1.90 | -7.70 (-8.94, -6.43) * |  | 27.19 | 23.91 | -2.57 (-3.72, -1.40) * |
| Azerbaijan | 257.90 | 213.47 | -2.67 (-2.69, -2.66) * |  | 97.42 | 75.72 | -3.44 (-3.87, -3.02) * |  | 172.39 | 154.57 | -1.48 (-1.69, -1.27) * |  | 2650.55 | 3386.76 | 3.55 (3.47, 3.63) * |
| Bahamas | 232.12 | 204.82 | -1.75 (-1.91, -1.58) * |  | 35.57 | 30.53 | -2.15 (-2.75, -1.55) * |  | 4.60 | 4.56 | -0.81 (-4.41, 2.93) |  | 27.37 | 36.03 | 3.14 (-0.51, 6.92) |
| Bahrain | 142.69 | 118.94 | -2.52 (-2.66, -2.38) * |  | 36.71 | 28.57 | -3.66 (-4.49, -2.82) * |  | 11.42 | 8.20 | -5.27 (-8.20, -2.25) * |  | 29.49 | 28.92 | -1.11 (-4.37, 2.26) |
| Bangladesh | 100.03 | 75.51 | -3.91 (-4.04, -3.79) * |  | 280.20 | 206.38 | -4.20 (-4.37, -4.03) * |  | 82.37 | 75.82 | -1.09 (-1.37, -0.82) * |  | 145.20 | 185.83 | 3.63 (3.46, 3.80) * |
| Barbados | 202.32 | 176.93 | -1.88 (-1.94, -1.81) * |  | 24.48 | 19.13 | -4.25 (-6.13, -2.32) * |  | 0.59 | 0.62 | -0.32 (-2.04, 1.43) |  | 3.34 | 4.89 | 4.21 (2.15, 6.32) * |
| Belarus | 181.81 | 143.22 | -3.33 (-3.72, -2.94) * |  | 47.84 | 28.59 | -6.90 (-9.60, -4.12) * |  | 259.18 | 172.28 | -5.54 (-7.35, -3.69) * |  | 3779.78 | 3774.68 | 0.04 (-1.24, 1.34) |
| Belgium | 74.66 | 66.17 | -1.68 (-1.86, -1.49) * |  | 8.17 | 6.72 | -3.34 (-4.74, -1.92) * |  | 1.63 | 1.15 | -5.54 (-7.89, -3.14) * |  | 14.29 | 14.40 | -0.56 (-2.65, 1.57) |
| Belize | 258.60 | 226.36 | -1.88 (-2.06, -1.70) * |  | 68.65 | 58.27 | -2.08 (-3.00, -1.15) * |  | 2.33 | 2.62 | 1.24 (-0.70, 3.21) |  | 13.21 | 20.71 | 5.95 (3.54, 8.42) * |
| Benin | 281.01 | 223.95 | -3.16 (-3.63, -2.69) * |  | 383.90 | 318.80 | -2.54 (-3.00, -2.08) * |  | 56.69 | 59.40 | 0.84 (0.56, 1.12) * |  | 26.42 | 38.33 | 5.59 (5.22, 5.96) * |
| Bhutan | 76.22 | 59.30 | -3.45 (-3.57, -3.33) * |  | 167.68 | 131.52 | -3.00 (-3.75, -2.25) * |  | 69.14 | 54.66 | -3.16 (-3.76, -2.55) * |  | 124.67 | 133.98 | 1.13 (0.31, 1.95) * |
| Bolivia | 235.01 | 186.39 | -3.49 (-3.95, -3.03) * |  | 168.46 | 124.09 | -4.28 (-4.32, -4.23) * |  | 59.95 | 60.95 | -0.18 (-2.52, 2.22) |  | 341.45 | 481.35 | 4.38 (1.61, 7.22) * |
| Bosnia and Herzegovina | 189.41 | 160.46 | -2.31 (-2.39, -2.23) * |  | 28.27 | 22.85 | -3.16 (-3.96, -2.36) * |  | 1.53 | 1.04 | -5.35 (-7.23, -3.43) * |  | 23.22 | 22.74 | -0.28 (-1.96, 1.43) |
| Botswana | 191.03 | 153.96 | -2.93 (-3.1, -2.76) * |  | 503.45 | 425.13 | -2.00 (-2.56, -1.43) * |  | 179.33 | 206.97 | 2.35 (1.33, 3.38) * |  | 85.43 | 133.53 | 6.83 (5.57, 8.11) * |
| Brazil | 273.80 | 243.03 | -1.67 (-1.71, -1.62) * |  | 40.47 | 34.80 | -2.10 (-2.31, -1.90) * |  | 8.90 | 10.08 | 1.79 (1.40, 2.18) * |  | 50.84 | 79.60 | 6.55 (5.90, 7.20) * |
| Brunei Darussalam | 274.51 | 235.75 | -2.14 (-2.3, -1.99) * |  | 53.16 | 45.05 | -2.05 (-2.72, -1.38) * |  | 1.34 | 1.45 | 0.85 (-3.61, 5.51) |  | 12.10 | 18.17 | 5.49 (0.64, 10.58) * |
| Bulgaria | 197.35 | 162.39 | -2.74 (-2.81, -2.66) * |  | 27.40 | 21.74 | -3.67 (-4.66, -2.68) * |  | 11.49 | 5.09 | -11.20 (-13.41, -8.93) * |  | 165.77 | 111.55 | -5.72 (-7.42, -3.99) * |
| Burkina Faso | 291.59 | 236.69 | -2.89 (-3.28, -2.50) * |  | 486.53 | 417.97 | -2.01 (-2.25, -1.77) * |  | 144.88 | 119.72 | -2.41 (-3.54, -1.28) * |  | 67.55 | 77.24 | 2.13 (1.36, 2.90) * |
| Burundi | 297.95 | 264.55 | -1.66 (-1.79, -1.53) * |  | 868.81 | 811.60 | -0.99 (-1.09, -0.88) * |  | 224.25 | 252.60 | 1.48 (0.25, 2.72) * |  | 108.27 | 162.97 | 5.67 (4.24, 7.11) * |
| Cabo Verde | 193.45 | 142.36 | -4.25 (-4.46, -4.03) * |  | 247.80 | 182.32 | -4.36 (-4.61, -4.11) * |  | 75.79 | 60.73 | -2.95 (-4.12, -1.76) * |  | 36.18 | 39.18 | 1.24 (0.22, 2.26) * |
| Cambodia | 351.85 | 312.85 | -1.66 (-1.78, -1.54) * |  | 607.72 | 514.94 | -2.58 (-3.41, -1.75) * |  | 58.01 | 36.56 | -5.86 (-10.59, -0.87) * |  | 387.96 | 333.34 | -1.66 (-6.53, 3.46) |
| Cameroon | 289.24 | 215.51 | -4.11 (-4.41, -3.82) * |  | 390.83 | 317.69 | -2.62 (-3.11, -2.14) * |  | 94.84 | 73.10 | -3.26 (-4.25, -2.26) * |  | 44.24 | 47.16 | 1.23 (0.57, 1.90) * |
| Canada | 90.90 | 90.04 | -0.13 (-0.15, -0.10) * |  | 4.25 | 4.15 | -0.47 (-0.88, -0.05) * |  | 0.52 | 0.51 | -0.60 (-1.31, 0.11) |  | 4.66 | 6.37 | 4.15 (3.47, 4.82) * |
| Central African Republic | 418.82 | 407.62 | -0.30 (-0.43, -0.17) * |  | 762.75 | 789.74 | 0.63 (0.31, 0.95) * |  | 96.85 | 112.09 | 2.16 (1.58, 2.74) * |  | 46.21 | 72.32 | 6.63 (5.82, 7.45) * |
| Chad | 335.04 | 271.78 | -2.93 (-3.17, -2.70) * |  | 542.95 | 469.84 | -1.98 (-2.46, -1.51) * |  | 154.42 | 124.09 | -2.92 (-3.29, -2.55) * |  | 72.46 | 80.06 | 1.51 (1.36, 1.66) * |
| Chile | 154.92 | 135.56 | -1.86 (-2.00, -1.72) * |  | 19.94 | 17.04 | -1.96 (-2.49, -1.42) * |  | 1.97 | 1.80 | -1.15 (-2.01, -0.28) * |  | 17.82 | 22.66 | 3.48 (2.73, 4.23) * |
| China | 255.77 | 259.75 | 0.06 (-0.86, 0.98) |  | 91.33 | 76.28 | -3.23 (-4.58, -1.87) * |  | 42.78 | 32.62 | -4.05 (-7.18, -0.82) * |  | 281.94 | 297.39 | 0.46 (-2.60, 3.62) |
| Colombia | 248.24 | 207.42 | -2.54 (-2.58, -2.51) * |  | 33.76 | 27.51 | -2.96 (-3.15, -2.76) * |  | 12.32 | 11.42 | -1.15 (-1.39, -0.91) * |  | 70.85 | 90.19 | 3.38 (2.94, 3.83) * |
| Comoros | 274.46 | 232.36 | -2.36 (-2.59, -2.13) * |  | 481.26 | 427.04 | -1.94 (-3.41, -0.45) * |  | 207.87 | 328.30 | 7.16 (3.62, 10.82) * |  | 100.89 | 211.81 | 11.54 (7.74, 15.46) * |
| Congo | 354.40 | 321.40 | -1.27 (-1.44, -1.10) * |  | 472.80 | 428.07 | -1.34 (-1.52, -1.17) * |  | 79.62 | 87.28 | 1.11 (0.21, 2.02) * |  | 38.27 | 56.31 | 5.40 (4.30, 6.50) * |
| Costa Rica | 222.71 | 191.31 | -2.15 (-2.24, -2.07) * |  | 19.62 | 15.37 | -3.46 (-4.89, -2.00) * |  | 3.54 | 3.05 | -2.03 (-3.86, -0.16) * |  | 20.32 | 24.11 | 2.46 (0.72, 4.23) * |
| Croatia | 144.32 | 121.36 | -2.43 (-2.58, -2.28) * |  | 15.70 | 11.71 | -4.24 (-5.99, -2.45) * |  | 0.80 | 0.42 | -8.45 (-12.45, -4.26) * |  | 12.41 | 9.20 | -3.77 (-7.86, 0.51) |
| Cuba | 181.09 | 152.51 | -2.47 (-2.57, -2.37) * |  | 21.67 | 15.41 | -5.17 (-7.63, -2.64) * |  | 3.16 | 3.45 | 0.88 (-0.52, 2.30) |  | 17.99 | 27.27 | 5.68 (4.43, 6.95) * |
| Cyprus | 74.26 | 66.68 | -1.50 (-1.64, -1.36) * |  | 5.10 | 4.30 | -3.05 (-4.53, -1.55) * |  | 2.82 | 0.79 | -16.96 (-20.81, -12.93) * |  | 24.47 | 9.90 | -12.36 (-15.97, -8.58) * |
| Czechia | 145.19 | 123.51 | -2.28 (-2.34, -2.23) * |  | 10.42 | 8.26 | -3.82 (-5.65, -1.95) * |  | 2.14 | 1.62 | -4.27 (-6.06, -2.44) * |  | 32.05 | 35.48 | 1.02 (-0.51, 2.58) |
| Cote d'Ivoire | 295.75 | 237.38 | -3.05 (-3.56, -2.54) * |  | 407.98 | 345.59 | -2.11 (-2.77, -1.44) * |  | 121.51 | 113.24 | -0.58 (-1.18, 0.02) |  | 57.89 | 73.06 | 3.73 (3.16, 4.32) * |
| Democratic Republic of the Congo | 390.70 | 354.37 | -1.27 (-1.47, -1.06) * |  | 689.55 | 622.18 | -1.39 (-1.54, -1.25) * |  | 132.81 | 151.48 | 1.64 (0.70, 2.60) * |  | 64.08 | 97.73 | 5.92 (4.82, 7.02) * |
| Denmark | 75.49 | 67.40 | -1.60 (-1.75, -1.45) * |  | 7.53 | 5.80 | -4.25 (-6.08, -2.39) * |  | 1.01 | 0.95 | -1.86 (-3.35, -0.35) * |  | 9.36 | 11.92 | 2.40 (0.75, 4.07) * |
| Djibouti | 263.14 | 191.41 | -4.41 (-4.52, -4.31) * |  | 489.04 | 371.50 | -3.66 (-4.38, -2.93) * |  | 245.40 | 260.20 | 0.55 (-1.33, 2.47) |  | 119.11 | 167.87 | 4.61 (2.56, 6.70) * |
| Dominica | 254.54 | 231.23 | -1.34 (-1.45, -1.23) * |  | 60.14 | 54.70 | -1.19 (-1.83, -0.54) * |  | 2.80 | 3.23 | 1.72 (-0.34, 3.82) |  | 15.85 | 25.50 | 6.50 (3.95, 9.10) * |
| Dominican Republic | 262.79 | 225.26 | -2.21 (-2.64, -1.77) |  | 98.17 | 93.13 | -0.41 (-1.94, 1.14) |  | 3.45 | 4.72 | 4.47 (1.28, 7.75) * |  | 19.51 | 37.30 | 9.44 (5.72, 13.29) * |
| Ecuador | 176.60 | 144.19 | -2.85 (-2.90, -2.80) * |  | 74.65 | 58.03 | -3.22 (-4.35, -2.08) * |  | 38.36 | 30.24 | -3.14 (-4.35, -1.91) * |  | 220.32 | 238.83 | 1.30 (0.24, 2.37) * |
| Egypt | 334.40 | 289.35 | -2.08 (-2.45, -1.71) * |  | 49.47 | 39.59 | -3.76 (-4.90, -2.59) * |  | 25.41 | 18.23 | -5.08 (-6.84, -3.28) * |  | 63.64 | 64.33 | -0.34 (-1.79, 1.12) |
| El Salvador | 281.51 | 240.21 | -2.26 (-2.37, -2.14) * |  | 26.87 | 22.41 | -2.80 (-3.28, -2.32) * |  | 4.97 | 4.13 | -2.79 (-3.61, -1.97) * |  | 28.39 | 32.62 | 1.73 (1.08, 2.39) * |
| Equatorial Guinea | 306.66 | 271.34 | -1.61 (-1.79, -1.43) * |  | 376.78 | 321.72 | -2.38 (-2.99, -1.77) * |  | 71.99 | 80.47 | 1.16 (0.45, 1.88) * |  | 34.60 | 51.92 | 5.43 (4.56, 6.31) * |
| Estonia | 158.66 | 132.07 | -2.55 (-2.75, -2.36) * |  | 32.71 | 25.25 | -3.47 (-5.45, -1.45) * |  | 95.19 | 53.21 | -8.18 (-10.76, -5.53) * |  | 1409.22 | 1165.95 | -2.89 (-5.14, -0.59) * |
| Eswatini | 316.31 | 257.10 | -2.96 (-3.15, -2.77) * |  | 479.00 | 436.49 | -0.92 (-1.51, -0.34) * |  | 503.46 | 487.92 | 0.15 (-0.98, 1.30) |  | 237.14 | 314.80 | 4.70 (3.73, 5.68) * |
| Ethiopia | 357.16 | 352.08 | -0.25 (-0.53, 0.03) |  | 462.08 | 396.56 | -2.11 (-2.77, -1.44) * |  | 111.93 | 128.72 | 1.81 (1.14, 2.49) * |  | 53.40 | 83.05 | 6.21 (5.28, 7.15) * |
| Finland | 72.60 | 65.07 | -1.54 (-1.68, -1.41) * |  | 6.80 | 5.42 | -3.55 (-5.12, -1.96) * |  | 1.67 | 1.79 | 0.53 (-0.40, 1.46) |  | 15.27 | 22.54 | 5.05 (4.00, 6.12) * |
| France | 73.10 | 65.31 | -1.6 (-1.75, -1.45) * |  | 8.26 | 6.84 | -2.96 (-4.05, -1.85) * |  | 1.36 | 1.24 | -1.59 (-2.60, -0.58) * |  | 12.27 | 15.65 | 3.16 (2.27, 4.06) * |
| Gabon | 316.02 | 278.33 | -1.64 (-1.91, -1.37) * |  | 379.34 | 337.24 | -1.61 (-1.69, -1.53) * |  | 91.62 | 97.13 | 0.66 (-0.11, 1.43) |  | 44.05 | 62.67 | 4.91 (3.94, 5.88) * |
| Gambia | 245.08 | 189.41 | -3.60 (-3.92, -3.28) * |  | 799.27 | 620.54 | -3.80 (-4.20, -3.39) * |  | 202.86 | 141.39 | -5.13 (-6.16, -4.09) * |  | 94.21 | 91.22 | -0.60 (-1.28, 0.08) |
| Georgia | 231.54 | 197.38 | -2.27 (-2.52, -2.02) * |  | 88.67 | 67.91 | -3.75 (-5.17, -2.30) * |  | 145.62 | 135.45 | -1.17 (-1.74, -0.59) * |  | 2229.10 | 2967.79 | 3.94 (3.50, 4.39) * |
| Germany | 74.89 | 67.45 | -1.48 (-1.57, -1.38) * |  | 6.68 | 5.97 | -2.25 (-3.34, -1.14) * |  | 1.79 | 2.29 | 2.55 (0.42, 4.73) * |  | 16.45 | 28.84 | 7.12 (4.80, 9.48) * |
| Ghana | 319.59 | 247.99 | -3.56 (-4.1, -3.01) * |  | 437.26 | 360.95 | -2.28 (-3.41, -1.13) * |  | 108.21 | 91.49 | -1.83 (-2.61, -1.05) * |  | 50.89 | 59.03 | 2.61 (1.74, 3.49) * |
| Greece | 74.25 | 68.72 | -1.06 (-1.13, -0.99) * |  | 6.37 | 5.56 | -1.78 (-2.53, -1.03) * |  | 2.97 | 1.58 | -8.36 (-10.06, -6.63) * |  | 25.41 | 19.92 | -3.21 (-4.40, -2.01) * |
| Grenada | 247.86 | 211.32 | -2.25 (-2.52, -1.99) * |  | 39.32 | 29.91 | -4.72 (-6.87, -2.51) * |  | 1.66 | 2.19 | 2.95 (0.9, 5.04) * |  | 9.44 | 17.31 | 7.67 (5.22, 10.17) * |
| Guatemala | 329.23 | 294.50 | -1.54 (-1.72, -1.37) * |  | 37.66 | 32.15 | -2.18 (-2.38, -1.98) * |  | 9.79 | 8.02 | -2.68 (-3.19, -2.16) * |  | 55.83 | 63.33 | 1.88 (1.61, 2.15) * |
| Guinea | 322.48 | 259.92 | -3.01 (-3.44, -2.57) * |  | 462.20 | 402.58 | -1.83 (-2.65, -1) * |  | 110.15 | 87.07 | -2.98 (-3.67, -2.28) * |  | 51.43 | 56.17 | 1.51 (1.10, 1.93) * |
| Guinea-Bissau | 326.71 | 264.20 | -2.97 (-3.28, -2.66) * |  | 354.73 | 312.64 | -1.45 (-2.27, -0.62) * |  | 77.14 | 56.83 | -3.56 (-4.95, -2.15) * |  | 36.11 | 36.66 | 0.86 (-0.28, 2.02) |
| Guyana | 306.68 | 260.66 | -2.31 (-2.46, -2.16) * |  | 107.16 | 87.04 | -2.73 (-3.02, -2.43) * |  | 5.07 | 5.86 | 1.51 (-1.63, 4.76) |  | 29.00 | 46.28 | 6.11 (2.55, 9.80) * |
| Haiti | 381.96 | 341.81 | -1.58 (-1.6, -1.55) * |  | 130.98 | 121.83 | -0.72 (-1.18, -0.26) * |  | 3.80 | 4.82 | 3.48 (1.45, 5.55) * |  | 21.28 | 38.10 | 8.55 (5.99, 11.18) * |
| Honduras | 335.97 | 295.54 | -1.83 (-1.95, -1.71) * |  | 61.90 | 54.73 | -1.52 (-1.87, -1.18) * |  | 14.76 | 13.70 | -0.85 (-1.23, -0.47) * |  | 84.63 | 108.20 | 3.71 (3.15, 4.27) * |
| Hungary | 163.22 | 136.69 | -2.51 (-2.55, -2.47) * |  | 15.08 | 10.47 | -5.4 (-7.47, -3.28) * |  | 4.19 | 1.35 | -14.79 (-20.49, -8.67) * |  | 60.47 | 29.64 | -9.43 (-14.89, -3.62) * |
| Iceland | 67.38 | 62.09 | -1.15 (-1.17, -1.13) * |  | 5.71 | 4.80 | -3.07 (-4.66, -1.45) * |  | 2.04 | 0.44 | -20.15 (-24.13, -15.96) * |  | 17.90 | 5.56 | -15.83 (-19.69, -11.77) * |
| India | 275.74 | 267.17 | -0.07 (-0.69, 0.55) |  | 409.58 | 363.64 | -1.44 (-1.79, -1.09) * |  | 242.84 | 257.63 | 1.17 (0.67, 1.67) * |  | 437.49 | 631.44 | 5.65 (5.07, 6.24) * |
| Indonesia | 351.16 | 343.07 | -0.34 (-0.39, -0.28) * |  | 287.35 | 235.49 | -2.74 (-3.77, -1.69) * |  | 17.98 | 9.09 | -8.6 (-13.33, -3.62) * |  | 119.16 | 82.89 | -4.39 (-9.17, 0.65) |
| Iran | 269.33 | 253.65 | -0.84 (-0.87, -0.82) * |  | 19.23 | 16.53 | -2.42 (-2.9, -1.94) * |  | 3.01 | 2.90 | -0.97 (-1.78, -0.16) * |  | 7.77 | 10.22 | 3.47 (2.7, 4.26) * |
| Iraq | 188.87 | 151.89 | -3.03 (-3.15, -2.9) * |  | 84.78 | 62.00 | -4.25 (-4.53, -3.98) * |  | 20.57 | 18.04 | -1.74 (-2.15, -1.34) * |  | 53.44 | 63.66 | 2.57 (2.31, 2.83) * |
| Ireland | 69.84 | 62.13 | -1.64 (-1.79, -1.49) * |  | 7.62 | 6.05 | -3.69 (-5.22, -2.14) * |  | 1.25 | 0.80 | -6.79 (-8.39, -5.16) * |  | 11.18 | 10.04 | -2.11 (-3.56, -0.64) * |
| Israel | 82.10 | 73.06 | -1.64 (-1.73, -1.55) * |  | 5.50 | 4.24 | -4.06 (-5.78, -2.3) * |  | 2.93 | 2.47 | -2.85 (-3.61, -2.08) * |  | 26.17 | 31.08 | 1.93 (1.14, 2.73) * |
| Italy | 85.76 | 79.14 | -1 (-1.26, -0.75) * |  | 5.40 | 4.44 | -3.2 (-4.98, -1.39) * |  | 1.72 | 1.19 | -5.48 (-7.24, -3.69) * |  | 15.54 | 14.98 | -0.94 (-2.6, 0.74) |
| Jamaica | 222.04 | 198.15 | -1.58 (-1.8, -1.36) * |  | 17.52 | 13.58 | -4.09 (-5.96, -2.17) * |  | 1.76 | 1.96 | 0.48 (-1.59, 2.59) |  | 10.33 | 15.48 | 4.63 (2.37, 6.93) * |
| Japan | 180.46 | 152.28 | -2.41 (-2.52, -2.29) * |  | 9.31 | 7.02 | -3.99 (-4.56, -3.4) * |  | 0.68 | 0.57 | -2.28 (-2.37, -2.19) * |  | 6.11 | 7.22 | 2.37 (2.13, 2.62) * |
| Jordan | 32.72 | 26.63 | -2.81 (-2.99, -2.62) * |  | 10.40 | 7.96 | -3.85 (-4.94, -2.74) * |  | 5.61 | 3.23 | -7.39 (-9.59, -5.15) * |  | 14.60 | 11.41 | -3.29 (-5.42, -1.11) * |
| Kazakhstan | 234.78 | 183.39 | -3.49 (-3.78, -3.21) * |  | 92.19 | 64.90 | -4.59 (-6.73, -2.39) * |  | 421.26 | 168.39 | -12.55 (-15.33, -9.68) * |  | 6142.61 | 3689.57 | -7.27 (-9.66, -4.82) * |
| Kenya | 292.27 | 287.39 | -0.3 (-0.41, -0.2) * |  | 473.61 | 445.59 | -0.77 (-0.9, -0.65) * |  | 68.37 | 68.89 | 0.27 (-1.85, 2.43) |  | 32.61 | 44.45 | 4.61 (2.61, 6.65) * |
| Kiribati | 436.37 | 435.01 | -0.08 (-0.24, 0.08) |  | 497.71 | 492.92 | 0.05 (-0.48, 0.58) |  | 12.98 | 13.62 | 0.69 (-0.52, 1.92) |  | 83.36 | 124.15 | 5.72 (3.97, 7.51) * |
| Kuwait | 120.25 | 100.48 | -2.5 (-2.76, -2.24) * |  | 33.82 | 24.97 | -4.38 (-6.13, -2.59) * |  | 5.20 | 3.98 | -4.33 (-5.24, -3.4) * |  | 13.41 | 14.04 | 0.01 (-0.96, 0.98) |
| Kyrgyzstan | 259.62 | 213.28 | -2.81 (-2.93, -2.7) * |  | 112.88 | 85.33 | -4.03 (-4.43, -3.63) * |  | 421.20 | 277.75 | -5.73 (-6.5, -4.95) * |  | 6218.01 | 6085.65 | -0.31 (-0.61, -0.02) * |
| Laos | 174.05 | 157.09 | -1.47 (-1.52, -1.42) * |  | 308.45 | 277.11 | -1.49 (-1.84, -1.15) * |  | 20.57 | 12.69 | -5.97 (-9.84, -1.93) * |  | 136.53 | 115.68 | -1.7 (-5.59, 2.35) |
| Latvia | 185.09 | 151.57 | -2.84 (-2.91, -2.76) * |  | 50.37 | 37.71 | -4.32 (-5.59, -3.04) * |  | 64.05 | 39.34 | -7.01 (-9.88, -4.05) * |  | 982.55 | 861.87 | -2.15 (-4.96, 0.73) |
| Lebanon | 122.61 | 103.19 | -2.41 (-2.56, -2.26) * |  | 33.09 | 26.04 | -3.77 (-5.02, -2.51) * |  | 5.15 | 4.78 | -1.54 (-2.32, -0.76) * |  | 13.29 | 16.88 | 2.86 (2, 3.72) * |
| Lesotho | 343.76 | 290.75 | -2.39 (-2.44, -2.33) |  | 684.87 | 661.10 | -0.2 (-1.03, 0.62) |  | 250.14 | 290.30 | 2.5 (1.64, 3.37) * |  | 119.15 | 187.29 | 7 (6.26, 7.74) * |
| Liberia | 281.95 | 229.67 | -2.84 (-3.24, -2.43) * |  | 436.73 | 364.44 | -2.45 (-2.69, -2.2) * |  | 87.15 | 71.46 | -2.42 (-3.43, -1.39) * |  | 41.10 | 46.10 | 1.92 (1.18, 2.66) * |
| Libya | 159.28 | 147.96 | -0.87 (-1.16, -0.59) * |  | 35.09 | 32.08 | -1.45 (-1.97, -0.93) * |  | 6.48 | 6.11 | -1.19 (-1.86, -0.51) * |  | 16.53 | 21.58 | 3.35 (2.32, 4.4) * |
| Lithuania | 185.81 | 156.27 | -2.42 (-2.47, -2.38) * |  | 71.56 | 53.52 | -4 (-4.66, -3.34) * |  | 139.66 | 106.83 | -3.74 (-4.55, -2.93) * |  | 2115.34 | 2340.71 | 1.4 (0.87, 1.94) * |
| Luxembourg | 70.43 | 63.90 | -1.35 (-1.46, -1.25) * |  | 7.08 | 5.86 | -3.53 (-5.57, -1.45) * |  | 2.83 | 1.48 | -9.76 (-11.24, -8.25) * |  | 24.35 | 18.66 | -4.71 (-6.24, -3.16) * |
| Madagascar | 300.71 | 256.87 | -2.23 (-2.53, -1.94) * |  | 759.49 | 646.03 | -2.45 (-3.07, -1.83) * |  | 123.83 | 172.92 | 3.99 (1.08, 6.98) * |  | 60.92 | 111.56 | 8.02 (5.03, 11.1) * |
| Malawi | 175.64 | 145.89 | -2.6 (-2.79, -2.41) * |  | 513.26 | 444.80 | -2.09 (-2.23, -1.95) * |  | 54.06 | 84.20 | 6.34 (5.63, 7.06) * |  | 25.42 | 54.32 | 11.16 (10.05, 12.28) * |
| Malaysia | 256.28 | 250.56 | -0.38 (-0.46, -0.3) |  | 129.91 | 124.39 | -0.95 (-1.94, 0.04) |  | 11.64 | 10.13 | -1.99 (-4.78, 0.87) |  | 77.07 | 92.40 | 2.49 (-0.27, 5.31) |
| Maldives | 226.39 | 221.44 | -0.33 (-0.44, -0.22) * |  | 80.76 | 72.62 | -2.19 (-3.87, -0.48) * |  | 5.58 | 3.06 | -8.21 (-12.95, -3.2) * |  | 36.76 | 27.91 | -3.87 (-8.63, 1.14) |
| Mali | 301.70 | 239.04 | -3.24 (-3.56, -2.92) * |  | 418.70 | 343.88 | -2.55 (-2.94, -2.15) * |  | 111.00 | 90.84 | -2.54 (-3.29, -1.78) * |  | 52.07 | 58.60 | 1.89 (1.45, 2.33) * |
| Malta | 78.37 | 69.42 | -1.7 (-1.78, -1.62) * |  | 7.96 | 7.06 | -2.51 (-3.93, -1.07) * |  | 1.73 | 0.53 | -16.28 (-19.01, -13.46) * |  | 15.47 | 6.61 | -12.04 (-14.72, -9.28) * |
| Marshall Islands | 410.02 | 404.53 | -0.2 (-0.22, -0.18) * |  | 212.68 | 198.34 | -1.1 (-1.52, -0.69) * |  | 39.98 | 19.78 | -9.01 (-12.93, -4.91) * |  | 248.97 | 180.37 | -3.93 (-7.44, -0.28) * |
| Mauritania | 263.29 | 194.56 | -4.24 (-4.41, -4.06) * |  | 259.01 | 190.27 | -4.46 (-4.83, -4.09) * |  | 64.19 | 43.73 | -5.36 (-6.84, -3.84) * |  | 30.25 | 28.21 | -1.06 (-2.30, 0.20) |
| Mauritius | 247.10 | 247.75 | 0.04 (-0.02, 0.09) |  | 44.29 | 39.63 | -2.20 (-4.32, -0.03) * |  | 4.42 | 1.93 | -11.31 (-15.19, -7.26) * |  | 27.66 | 17.59 | -6.43 (-9.93, -2.80) * |
| Mexico | 285.87 | 250.51 | -1.87 (-2.02, -1.71) |  | 26.65 | 26.26 | 0.34 (-0.50, 1.18) |  | 6.96 | 7.98 | 2.56 (1.48, 3.66) * |  | 39.30 | 63.05 | 7.56 (6.61, 8.51) * |
| Micronesia (Federated States of) | 387.07 | 377.97 | -0.35 (-0.38, -0.33) * |  | 180.82 | 164.69 | -1.49 (-2.05, -0.91) * |  | 4.99 | 4.98 | -0.34 (-1.53, 0.86) |  | 32.63 | 45.37 | 4.38 (2.82, 5.97) * |
| Mongolia | 294.04 | 248.31 | -2.39 (-2.54, -2.24) * |  | 187.90 | 152.81 | -2.49 (-3.12, -1.86) * |  | 151.51 | 159.15 | 0.84 (-0.76, 2.47) |  | 2334.46 | 3487.10 | 5.97 (4.08, 7.89) * |
| Montenegro | 167.66 | 140.57 | -2.48 (-2.54, -2.42) * |  | 15.19 | 12.07 | -3.61 (-4.86, -2.35) * |  | 1.90 | 1.10 | -7.37 (-8.38, -6.34) * |  | 29.10 | 23.99 | -2.51 (-3.77, -1.23) * |
| Morocco | 218.30 | 174.89 | -3.10 (-3.23, -2.96) * |  | 156.26 | 122.49 | -2.90 (-3.80, -2.00) * |  | 25.86 | 24.56 | -0.36 (-0.90, 0.18) |  | 66.55 | 86.70 | 4.13 (3.54, 4.73) * |
| Mozambique | 301.63 | 252.57 | -2.50 (-2.72, -2.28) |  | 570.61 | 502.18 | -1.37 (-2.77, 0.05) |  | 239.59 | 258.64 | 1.39 (0.02, 2.77) * |  | 114.72 | 166.86 | 5.71 (4.10, 7.33) * |
| Namibia | 278.50 | 219.64 | -3.39 (-3.61, -3.17) * |  | 601.95 | 482.33 | -3.26 (-3.88, -2.63) * |  | 304.53 | 289.25 | -0.93 (-2.44, 0.61) |  | 144.06 | 186.61 | 3.46 (2.10, 4.82) * |
| Nauru | 366.28 | 338.33 | -1.14 (-1.25, -1.04) * |  | 181.30 | 146.35 | -3.07 (-4.12, -2.02) * |  | 4.65 | 4.15 | -1.78 (-2.31, -1.25) * |  | 30.21 | 37.87 | 2.95 (2.03, 3.87) * |
| Nepal | 165.20 | 139.68 | -1.97 (-2.60, -1.33) * |  | 346.27 | 259.71 | -3.76 (-4.14, -3.37) * |  | 133.45 | 123.83 | -0.74 (-1.49, 0.01) |  | 237.22 | 303.50 | 3.88 (3.34, 4.42) * |
| Netherlands | 68.67 | 62.00 | -1.44 (-1.55, -1.32) * |  | 5.74 | 4.72 | -3.26 (-4.79, -1.70) * |  | 1.22 | 0.91 | -4.74 (-6.20, -3.26) * |  | 10.68 | 11.50 | 0.23 (-1.11, 1.59) |
| Nicaragua | 276.29 | 231.14 | -2.53 (-2.57, -2.49) * |  | 46.06 | 37.24 | -2.92 (-3.20, -2.64) * |  | 7.24 | 6.73 | -0.94 (-1.67, -0.20) * |  | 41.56 | 53.13 | 3.63 (3.06, 4.20) * |
| Niger | 311.66 | 254.11 | -2.86 (-3.09, -2.63) * |  | 394.85 | 352.48 | -1.49 (-1.96, -1.02) * |  | 103.88 | 84.10 | -2.59 (-3.26, -1.91) * |  | 49.42 | 54.26 | 1.65 (1.13, 2.17) * |
| Nigeria | 277.86 | 230.16 | -2.62 (-3.17, -2.07) * |  | 376.92 | 316.75 | -2.08 (-2.94, -1.21) * |  | 146.12 | 130.80 | -1.05 (-1.86, -0.24) * |  | 68.74 | 84.39 | 3.43 (2.72, 4.15) * |
| North Macedonia | 185.72 | 157.68 | -2.31 (-2.34, -2.28) * |  | 20.47 | 17.21 | -2.58 (-3.30, -1.86) * |  | 4.33 | 2.29 | -8.79 (-9.43, -8.15) * |  | 64.41 | 50.21 | -3.62 (-3.91, -3.33) * |
| Norway | 73.61 | 66.49 | -1.42 (-1.56, -1.28) * |  | 4.85 | 3.63 | -4.27 (-5.50, -3.03) * |  | 1.29 | 0.89 | -5.38 (-6.81, -3.92) * |  | 11.29 | 11.19 | -0.39 (-1.46, 0.70) |
| Oman | 139.33 | 115.64 | -2.61 (-2.67, -2.55) * |  | 34.51 | 24.87 | -4.95 (-6.27, -3.60) * |  | 6.29 | 4.71 | -4.77 (-6.34, -3.16) * |  | 16.14 | 16.63 | -0.43 (-2.31, 1.49) |
| Pakistan | 177.04 | 141.38 | -3.13 (-3.19, -3.06) * |  | 519.65 | 437.76 | -2.24 (-3.25, -1.22) * |  | 230.11 | 246.34 | 1.11 (0.30, 1.93) * |  | 412.82 | 603.78 | 5.64 (4.54, 6.74) * |
| Palau | 278.32 | 278.37 | -0.01 (-0.09, 0.08) |  | 93.92 | 84.18 | -1.96 (-3.09, -0.82) * |  | 2.53 | 2.51 | -0.72 (-1.65, 0.21) |  | 16.43 | 22.91 | 4.03 (2.84, 5.23) * |
| Panama | 257.58 | 210.88 | -2.88 (-3.04, -2.72) * |  | 63.18 | 54.61 | -1.74 (-2.96, -0.50) * |  | 14.35 | 12.24 | -1.85 (-2.76, -0.93) * |  | 81.10 | 96.67 | 2.84 (1.63, 4.06) * |
| Papua New Guinea | 425.93 | 421.86 | -0.13 (-0.16, -0.11) * |  | 237.25 | 220.93 | -1.06 (-1.51, -0.61) * |  | 50.12 | 81.05 | 5.53 (0.17, 11.19) * |  | 346.88 | 739.00 | 9.72 (4.33, 15.38) * |
| Paraguay | 276.00 | 227.45 | -2.74 (-2.77, -2.70) * |  | 55.95 | 46.42 | -2.39 (-2.72, -2.06) * |  | 13.15 | 15.02 | 2.06 (1.38, 2.75) * |  | 74.66 | 118.59 | 6.93 (5.94, 7.94) * |
| Peru | 166.67 | 133.57 | -3.11 (-3.25, -2.97) * |  | 106.10 | 78.90 | -3.96 (-4.64, -3.27) * |  | 83.47 | 61.16 | -3.74 (-7.24, -0.10) * |  | 483.90 | 482.99 | 0.49 (-3.06, 4.17) |
| Philippines | 369.77 | 380.11 | 0.39 (0.36, 0.42) |  | 394.17 | 426.42 | 0.57 (-0.46, 1.61) |  | 107.86 | 117.40 | 0.97 (0.57, 1.38) * |  | 710.43 | 1070.43 | 5.69 (5.25, 6.13) * |
| Poland | 181.11 | 150.69 | -2.57 (-2.70, -2.44) * |  | 17.13 | 13.40 | -3.59 (-4.58, -2.59) * |  | 1.33 | 1.04 | -3.59 (-5.51, -1.62) * |  | 20.60 | 22.74 | 1.26 (-0.63, 3.18) |
| Portugal | 80.23 | 70.33 | -1.84 (-2.03, -1.66) * |  | 15.34 | 12.72 | -2.51 (-3.02, -2.00) * |  | 2.19 | 1.82 | -2.39 (-3.86, -0.89) * |  | 19.80 | 22.83 | 2.28 (0.94, 3.65) * |
| Qatar | 124.97 | 104.93 | -2.45 (-2.54, -2.36) * |  | 28.59 | 21.09 | -4.57 (-5.70, -3.42) * |  | 4.58 | 4.24 | -1.47 (-2.49, -0.45) * |  | 11.73 | 14.98 | 3.08 (2.21, 3.96) * |
| Republic of Korea | 183.42 | 150.97 | -2.75 (-2.93, -2.57) * |  | 35.18 | 26.15 | -3.83 (-4.44, -3.23) * |  | 5.04 | 3.53 | -4.47 (-5.31, -3.62) * |  | 45.63 | 44.38 | 0.06 (-0.67, 0.80) |
| Republic of Moldova | 229.39 | 182.84 | -3.17 (-3.32, -3.03) * |  | 87.32 | 50.96 | -7.37 (-8.85, -5.87) * |  | 406.79 | 308.51 | -3.75 (-4.05, -3.46) * |  | 6108.79 | 6759.77 | 1.49 (1.32, 1.66) * |
| Romania | 186.10 | 153.60 | -2.70 (-2.73, -2.66) * |  | 58.45 | 47.11 | -2.76 (-3.13, -2.39) * |  | 19.38 | 15.15 | -3.43 (-5.05, -1.79) * |  | 301.93 | 331.98 | 1.22 (-0.52, 2.99) |
| Russian Federation | 204.38 | 165.76 | -2.96 (-3.19, -2.74) * |  | 78.45 | 51.30 | -5.45 (-7.18, -3.68) * |  | 227.91 | 150.55 | -5.36 (-6.75, -3.96) * |  | 3441.13 | 3298.71 | -0.28 (-1.39, 0.84) |
| Rwanda | 272.11 | 218.90 | -3.06 (-3.19, -2.94) * |  | 388.43 | 322.55 | -2.36 (-2.87, -1.85) * |  | 85.87 | 96.02 | 1.83 (1.15, 2.51) * |  | 40.94 | 61.95 | 6.24 (5.73, 6.75) * |
| Saint Kitts and Nevis | 204.10 | 174.97 | -2.18 (-2.33, -2.03) * |  | 41.87 | 32.89 | -3.49 (-3.96, -3.02) * |  | 1.58 | 1.70 | 0.64 (-1.17, 2.48) |  | 8.96 | 13.46 | 5.30 (3.05, 7.59) * |
| Saint Lucia | 231.45 | 201.13 | -1.96 (-2.11, -1.81) * |  | 41.03 | 33.69 | -2.83 (-3.52, -2.14) * |  | 1.42 | 1.43 | -0.38 (-1.29, 0.54) |  | 8.03 | 11.27 | 4.36 (3.01, 5.73) * |
| Saint Vincent and the Grenadines | 256.85 | 226.87 | -1.75 (-2.04, -1.45) * |  | 44.24 | 37.00 | -2.66 (-3.70, -1.61) * |  | 1.74 | 1.88 | 0.43 (-0.87, 1.76) |  | 9.87 | 14.81 | 5.13 (3.39, 6.89) * |
| Sao Tome and Principe | 248.03 | 186.70 | -3.95 (-4.24, -3.66) * |  | 248.35 | 186.08 | -4.23 (-4.66, -3.79) * |  | 81.94 | 64.76 | -3.80 (-4.70, -2.89) * |  | 39.19 | 41.78 | 0.28 (-0.81, 1.38) |
| Saudi Arabia | 209.12 | 161.27 | -3.62 (-3.79, -3.45) * |  | 95.67 | 67.24 | -4.63 (-5.16, -4.09) * |  | 23.81 | 18.71 | -2.94 (-4.08, -1.79) * |  | 60.57 | 66.03 | 1.65 (0.78, 2.52) * |
| Senegal | 291.49 | 228.00 | -3.43 (-3.78, -3.08) * |  | 400.48 | 336.77 | -2.34 (-2.99, -1.68) * |  | 79.93 | 75.22 | -0.59 (-1.89, 0.74) |  | 38.53 | 48.53 | 3.51 (2.25, 4.79) * |
| Serbia | 176.26 | 148.20 | -2.39 (-2.56, -2.21) * |  | 19.80 | 15.24 | -3.98 (-5.44, -2.50) * |  | 2.04 | 1.31 | -6.13 (-9.16, -3.00) * |  | 30.16 | 28.81 | -0.75 (-3.47, 2.05) |
| Seychelles | 269.95 | 268.06 | -0.14 (-0.35, 0.06) |  | 80.43 | 74.56 | -1.73 (-3.13, -0.31) * |  | 2.22 | 1.64 | -4.51 (-8.11, -0.76) * |  | 14.68 | 14.92 | -0.09 (-3.69, 3.64) |
| Sierra Leone | 309.35 | 237.97 | -3.65 (-3.84, -3.46) * |  | 561.42 | 430.74 | -3.58 (-3.81, -3.35) * |  | 124.11 | 95.41 | -3.45 (-4.16, -2.73) * |  | 57.90 | 61.56 | 1.02 (0.67, 1.38) * |
| Singapore | 182.66 | 152.49 | -2.56 (-2.68, -2.43) * |  | 21.06 | 16.58 | -3.52 (-3.91, -3.13) * |  | 2.81 | 1.87 | -6.10 (-6.74, -5.44) * |  | 24.75 | 23.46 | -1.28 (-2.13, -0.42) * |
| Slovakia | 162.87 | 135.77 | -2.57 (-2.68, -2.45) * |  | 11.55 | 8.95 | -4.15 (-5.84, -2.43) * |  | 1.09 | 0.61 | -8.19 (-10.73, -5.58) * |  | 16.69 | 13.45 | -3.44 (-5.93, -0.89) * |
| Slovenia | 135.47 | 113.76 | -2.46 (-2.63, -2.29) * |  | 10.12 | 7.59 | -4.34 (-6.29, -2.34) * |  | 0.28 | 0.08 | -15.89 (-20.11, -11.45) * |  | 4.19 | 1.80 | -11.29 (-15.51, -6.85) * |
| Solomon Islands | 403.31 | 391.74 | -0.42 (-0.46, -0.38) * |  | 140.20 | 127.04 | -1.56 (-2.21, -0.90) * |  | 3.87 | 3.89 | -0.14 (-0.66, 0.38) |  | 24.96 | 35.45 | 4.77 (3.79, 5.75) * |
| South Africa | 449.12 | 436.77 | -0.36 (-0.47, -0.24) * |  | 606.52 | 562.68 | -1.03 (-1.95, -0.09) * |  | 155.86 | 148.27 | -0.51 (-3.63, 2.72) |  | 74.29 | 95.66 | 3.82 (0.76, 6.97) * |
| Spain | 29.23 | 24.20 | -2.68 (-2.80, -2.56) * |  | 6.12 | 4.89 | -3.36 (-4.16, -2.56) * |  | 0.85 | 0.57 | -5.82 (-6.80, -4.82) * |  | 7.66 | 7.18 | -1.19 (-2.02, -0.36) * |
| Sri Lanka | 263.05 | 264.23 | -0.02 (-0.15, 0.11) |  | 101.47 | 95.30 | -1.70 (-3.34, -0.03) * |  | 4.60 | 3.50 | -4.26 (-7.23, -1.20) * |  | 29.01 | 31.88 | 0.83 (-1.75, 3.48) |
| Sudan | 250.27 | 201.93 | -3.00 (-3.19, -2.81) * |  | 93.98 | 70.33 | -4.09 (-4.29, -3.89) * |  | 18.17 | 14.01 | -3.84 (-4.58, -3.08) * |  | 46.77 | 49.44 | 0.51 (-0.51, 1.55) |
| Suriname | 282.68 | 242.81 | -2.16 (-2.23, -2.09) * |  | 37.68 | 32.18 | -2.38 (-3.17, -1.59) * |  | 1.17 | 1.20 | -0.17 (-1.40, 1.08) |  | 6.61 | 9.46 | 4.49 (2.83, 6.18) * |
| Sweden | 161.69 | 155.86 | -0.56 (-0.62, -0.50) |  | 5.96 | 5.62 | -1.24 (-2.53, 0.06) |  | 2.03 | 1.71 | -2.85 (-4.29, -1.39) * |  | 17.90 | 21.54 | 2.12 (0.89, 3.37) * |
| Switzerland | 68.99 | 62.68 | -1.33 (-1.41, -1.25) * |  | 6.35 | 5.23 | -3.38 (-4.85, -1.89) * |  | 1.62 | 2.04 | 2.27 (0.13, 4.45) * |  | 15.14 | 25.70 | 6.60 (4.40, 8.84) * |
| Syrian Arab Republic | 160.42 | 140.58 | -1.84 (-1.98, -1.71) * |  | 28.03 | 23.75 | -2.79 (-3.72, -1.85) * |  | 8.12 | 6.26 | -4.10 (-4.85, -3.34) * |  | 21.06 | 22.10 | 0.16 (-0.62, 0.94) |
| Tajikistan | 303.29 | 263.88 | -1.93 (-2.09, -1.77) * |  | 119.41 | 100.33 | -2.29 (-2.59, -1.98) * |  | 172.31 | 159.93 | -1.11 (-1.54, -0.67) * |  | 2676.64 | 3504.15 | 3.76 (3.19, 4.34) * |
| Thailand | 236.21 | 232.76 | -0.23 (-0.27, -0.20) |  | 114.45 | 111.87 | -0.29 (-0.84, 0.25) |  | 28.18 | 26.35 | -0.64 (-2.54, 1.30) |  | 181.21 | 240.28 | 4.37 (2.80, 5.96) * |
| Timor-Leste | 374.71 | 370.74 | -0.16 (-0.29, -0.04) |  | 303.28 | 312.32 | 0.15 (-0.28, 0.58) |  | 21.75 | 14.67 | -5.08 (-8.05, -2.02) * |  | 144.12 | 133.76 | -0.74 (-3.67, 2.27) |
| Togo | 290.97 | 221.88 | -3.77 (-4.08, -3.45) * |  | 456.43 | 371.79 | -2.67 (-3.07, -2.28) * |  | 119.14 | 90.57 | -3.4 (-4.72, -2.06) * |  | 55.21 | 58.43 | 1.16 (0.24, 2.09) * |
| Trinidad and Tobago | 238.83 | 207.40 | -1.98 (-2.09, -1.87) * |  | 30.32 | 24.83 | -3.11 (-4.28, -1.93) * |  | 1.18 | 1.23 | -0.19 (-1.73, 1.37) |  | 6.68 | 9.72 | 4.41 (2.45, 6.40) * |
| Tunisia | 146.58 | 120.84 | -2.69 (-2.76, -2.62) * |  | 36.89 | 28.20 | -4.17 (-5.34, -2.98) * |  | 4.11 | 3.64 | -2.17 (-3.66, -0.65) * |  | 10.64 | 12.84 | 2.19 (0.79, 3.60) * |
| Turkey | 201.14 | 162.88 | -2.95 (-2.98, -2.91) * |  | 47.41 | 34.51 | -4.89 (-6.26, -3.50) * |  | 16.72 | 13.34 | -3.94 (-5.17, -2.70) * |  | 43.37 | 47.07 | 0.30 (-0.99, 1.61) |
| Turkmenistan | 262.62 | 217.81 | -2.55 (-2.68, -2.42) * |  | 114.40 | 92.33 | -2.67 (-3.14, -2.19) * |  | 184.15 | 164.00 | -1.52 (-2.54, -0.50) * |  | 2860.38 | 3593.27 | 3.33 (2.17, 4.50) * |
| Uganda | 444.34 | 395.80 | -1.62 (-1.85, -1.40) |  | 330.78 | 325.40 | -0.15 (-0.41, 0.11) |  | 76.67 | 106.18 | 4.75 (4.42, 5.09) * |  | 36.42 | 68.50 | 9.37 (8.76, 9.98) * |
| Ukraine | 202.48 | 176.65 | -1.90 (-1.98, -1.81) * |  | 69.33 | 61.73 | -1.18 (-2.05, -0.31) * |  | 218.24 | 235.90 | 1.15 (-1.76, 4.14) |  | 3405.15 | 5168.69 | 6.08 (2.92, 9.33) * |
| United Arab Emirates | 289.66 | 246.46 | -2.22 (-2.37, -2.07) * |  | 63.13 | 49.47 | -3.85 (-5.31, -2.36) * |  | 10.85 | 9.90 | -1.91 (-2.89, -0.92) * |  | 27.96 | 34.94 | 2.45 (1.36, 3.56) * |
| United Kingdom | 80.35 | 72.16 | -1.49 (-1.63, -1.36) * |  | 8.13 | 5.70 | -5.02 (-6.31, -3.71) * |  | 1.07 | 0.71 | -6.09 (-7.38, -4.77) * |  | 9.22 | 8.90 | -0.98 (-1.95, 0) * |
| United Republic of Tanzania | 193.92 | 148.22 | -3.78 (-4.08, -3.48) * |  | 460.29 | 400.90 | -1.92 (-2.89, -0.93) * |  | 76.46 | 100.19 | 3.67 (1.25, 6.14) * |  | 37.02 | 64.64 | 7.91 (5.28, 10.61) * |
| United States of America | 102.69 | 97.93 | -0.58 (-0.99, -0.17) * |  | 3.35 | 2.98 | -1.67 (-2.74, -0.58) * |  | 0.43 | 0.40 | -1.26 (-2.93, 0.43) |  | 3.82 | 5.05 | 3.76 (2.36, 5.18) * |
| Uruguay | 175.19 | 155.51 | -1.69 (-1.82, -1.56) * |  | 22.26 | 19.45 | -2.28 (-2.91, -1.64) * |  | 0.65 | 0.61 | -1.57 (-3.52, 0.42) |  | 6.04 | 7.68 | 2.63 (0.56, 4.73) * |
| Uzbekistan | 256.87 | 214.58 | -2.54 (-2.73, -2.34) * |  | 94.45 | 76.74 | -2.70 (-3.30, -2.10) * |  | 334.15 | 220.39 | -5.62 (-6.40, -4.83) * |  | 4941.96 | 4828.92 | -0.23 (-0.56, 0.09) |
| Vanuatu | 418.98 | 416.44 | -0.09 (-0.12, -0.05) * |  | 193.15 | 185.68 | -0.72 (-1.08, -0.37) * |  | 2.87 | 3.34 | 1.49 (-0.71, 3.74) |  | 19.29 | 30.47 | 5.89 (3.5, 8.33) * |
| Viet Nam | 429.72 | 413.17 | -0.61 (-0.71, -0.51) * |  | 183.18 | 164.92 | -1.45 (-1.60, -1.31) * |  | 80.19 | 65.23 | -2.48 (-4.53, -0.38) * |  | 510.72 | 594.81 | 2.54 (0.88, 4.23) * |
| Yemen | 244.66 | 219.15 | -1.51 (-1.77, -1.24) * |  | 91.58 | 81.02 | -1.89 (-2.69, -1.09) * |  | 19.72 | 17.49 | -1.73 (-3.60, 0.16) |  | 50.93 | 61.72 | 2.72 (1.00, 4.47) * |
| Zambia | 266.86 | 202.27 | -3.88 (-3.91, -3.85) * |  | 504.87 | 407.89 | -3.00 (-3.19, -2.82) * |  | 96.72 | 146.04 | 5.59 (3.47, 7.75) * |  | 46.45 | 94.22 | 10.06 (7.68, 12.49) * |
| Zimbabwe | 179.40 | 129.72 | -4.61 (-4.85, -4.37) * |  | 425.65 | 362.41 | -2.05 (-2.37, -1.74) * |  | 198.93 | 186.14 | -0.75 (-1.05, -0.45) * |  | 94.16 | 120.09 | 3.68 (3.26, 4.11) * |

Countries were arranged in alphabetical order.

TB: tuberculosis; DS-TB: drug-susceptible tuberculosis; MDR-TB: multidrug-resistant tuberculosis; XDR-TB: extensively drug-resistant tuberculosis.

a: per 1000 population; b: per 100 000 population; c: per 100 000 000 population; d: per 1 000 000 000 population; CI: confidence interval.

# Table S6: Estimated annual percentage change (EAPC, %) for mortality of TB from 2012 to 2019

| Country name | Total TB | | |  | DS-TB | | |  | MDR-TB | | |  | XDR-TB | | |
| --- | --- | --- | --- | --- | --- | --- | --- | --- | --- | --- | --- | --- | --- | --- | --- |
|  | 2012^a^ | 2019^a^ | EAPC (95% CI) |  | 2012^a^ | 2019^a^ | EAPC (95% CI) |  | 2012^b^ | 2019^b^ | EAPC (95% CI) |  | 2012^c^ | 2019^c^ | EAPC (95% CI) |
| Afghanistan | 3.85 | 2.39 | -6.35 (-7.5, -5.19) * |  | 3.69 | 2.28 | -6.41 (-7.59, -5.23) * |  | 1.56 | 1.05 | -5.30 (-6.05, -4.55) * |  | 8.51 | 7.82 | -1.08 (-1.81, -0.35) * |
| Albania | 29.89 | 21.91 | -4.3 (-4.41, -4.19) * |  | 26.26 | 19.74 | -3.98 (-4.10, -3.86) * |  | 34.50 | 20.22 | -7.08 (-7.57, -6.57) * |  | 188.29 | 150.80 | -2.85 (-3.27, -2.43) * |
| Algeria | 0.46 | 0.39 | -2.10 (-2.68, -1.52) * |  | 0.45 | 0.39 | -2.08 (-2.68, -1.48) * |  | 0.07 | 0.05 | -3.67 (-4.17, -3.16) * |  | 0.83 | 0.88 | 0.84 (0.49, 1.20) |
| Angola | 0.32 | 0.28 | -2.36 (-2.68, -2.03) * |  | 0.31 | 0.27 | -2.25 (-2.61, -1.90) * |  | 0.07 | 0.04 | -7.56 (-8.29, -6.83) * |  | 2.32 | 1.87 | -2.85 (-3.48, -2.22) |
| Antigua and Barbuda | 3.23 | 1.95 | -6.78 (-8.77, -4.74) * |  | 2.16 | 1.32 | -6.60 (-8.52, -4.63) * |  | 8.04 | 4.27 | -8.44 (-10.55, -6.29) * |  | 258.40 | 195.17 | -3.71 (-5.77, -1.61) * |
| Argentina | 115.51 | 78.83 | -5.47 (-5.77, -5.18) * |  | 108.69 | 73.71 | -5.56 (-5.89, -5.23) * |  | 67.53 | 50.48 | -4.20 (-4.73, -3.66) * |  | 67.77 | 68.56 | 0.03 (-0.67, 0.74) * |
| Armenia | 1.63 | 1.42 | -1.94 (-2.44, -1.44) * |  | 1.57 | 1.37 | -1.92 (-2.42, -1.41) * |  | 0.53 | 0.41 | -3.42 (-4.11, -2.72) * |  | 9.94 | 10.81 | 1.22 (0.46, 1.98) * |
| Australia | 0.34 | 0.28 | -3.53 (-4.56, -2.48) |  | 0.30 | 0.26 | -3.10 (-4.06, -2.13) * |  | 0.30 | 0.17 | -8.45 (-10.16, -6.70) * |  | 5.60 | 4.59 | -4.03 (-5.91, -2.1) * |
| Austria | 0.21 | 0.21 | -0.03 (-0.43, 0.36) * |  | 0.20 | 0.20 | -0.43 (-0.85, -0.01) * |  | 0.10 | 0.14 | 4.69 (3.37, 6.02) * |  | 1.84 | 3.57 | 9.75 (8.20, 11.33) * |
| Azerbaijan | 8.19 | 6.00 | -4.48 (-4.99, -3.97) * |  | 5.45 | 3.83 | -4.98 (-5.50, -4.47) * |  | 20.70 | 14.85 | -4.90 (-5.46, -4.34) * |  | 665.53 | 683.50 | 0.10 (-0.38, 0.59) |
| Bahamas | 2.72 | 2.09 | -3.71 (-4.09, -3.33) |  | 2.67 | 2.06 | -3.67 (-4.05, -3.30) |  | 0.34 | 0.19 | -7.39 (-9.26, -5.47) * |  | 11.02 | 8.83 | -2.58 (-4.39, -0.73) |
| Bahrain | 0.41 | 0.41 | 0.20 (-0.39, 0.80) * |  | 0.41 | 0.40 | 0.20 (-0.39, 0.80) * |  | 0.03 | 0.03 | -0.17 (-1.31, 0.99) * |  | 0.40 | 0.52 | 4.47 (3.41, 5.55) * |
| Bangladesh | 31.54 | 22.59 | -4.47 (-4.94, -4.00) * |  | 29.39 | 20.71 | -4.69 (-5.17, -4.21) * |  | 20.71 | 17.84 | -1.93 (-2.42, -1.43) * |  | 78.89 | 92.21 | 2.43 (1.94, 2.93) * |
| Barbados | 0.46 | 0.33 | -4.57 (-6.37, -2.75) |  | 0.44 | 0.32 | -4.51 (-6.27, -2.72) |  | 0.19 | 0.12 | -6.65 (-9.14, -4.09) |  | 3.62 | 3.16 | -2.08 (-4.52, 0.42) * |
| Belarus | 70.36 | 64.18 | -1.24 (-2.04, -0.43) * |  | 65.89 | 60.03 | -1.27 (-2.11, -0.42) * |  | 44.24 | 41.01 | -0.79 (-1.19, -0.38) * |  | 44.47 | 55.50 | 3.51 (2.99, 4.03) * |
| Belgium | 1.19 | 0.96 | -2.81 (-3.77, -1.84) * |  | 1.06 | 0.90 | -2.17 (-2.92, -1.41) * |  | 1.00 | 0.43 | -10.78 (-13.36, -8.12) * |  | 32.23 | 19.50 | -6.22 (-8.79, -3.58) |
| Belize | 2.50 | 1.65 | -5.68 (-6.91, -4.43) * |  | 2.25 | 1.54 | -5.19 (-6.47, -3.89) * |  | 2.36 | 1.03 | -11.17 (-11.71, -10.63) * |  | 12.86 | 7.70 | -7.14 (-7.63, -6.64) * |
| Benin | 173.55 | 154.67 | -1.70 (-1.88, -1.51) * |  | 163.33 | 143.89 | -1.86 (-2.07, -1.65) * |  | 101.27 | 106.39 | 0.67 (-0.23, 1.58) |  | 101.72 | 144.83 | 5.14 (4.02, 6.26) * |
| Bhutan | 54.43 | 43.83 | -2.99 (-3.89, -2.07) * |  | 52.49 | 41.88 | -3.12 (-4.05, -2.18) * |  | 19.24 | 19.21 | 0.22 (-0.36, 0.80) * |  | 19.37 | 26.28 | 4.70 (3.96, 5.43) * |
| Bolivia | 10.55 | 9.47 | -1.61 (-2.46, -0.75) * |  | 10.47 | 9.39 | -1.61 (-2.42, -0.79) * |  | 0.65 | 0.66 | -2.11 (-7.78, 3.91) * |  | 12.36 | 17.59 | 2.63 (-3.46, 9.09) |
| Bosnia and Herzegovina | 23.07 | 16.62 | -5.25 (-6.99, -3.49) * |  | 21.00 | 14.79 | -5.56 (-7.25, -3.85) * |  | 18.46 | 15.78 | -3 (-5.48, -0.46) * |  | 223.30 | 260.63 | 1.41 (-1.32, 4.21) * |
| Botswana | 3.05 | 2.32 | -3.79 (-4.09, -3.49) * |  | 2.88 | 2.16 | -3.99 (-4.29, -3.70) * |  | 1.46 | 1.34 | -1.11 (-1.73, -0.50) * |  | 17.58 | 22.25 | 3.47 (2.64, 4.30) * |
| Brazil | 1.96 | 1.87 | -0.11 (-0.96, 0.75) * |  | 1.86 | 1.80 | 0.12 (-0.73, 0.98) * |  | 0.94 | 0.61 | -5.43 (-6.5, -4.35) * |  | 11.37 | 10.08 | -1.03 (-2.11, 0.07) * |
| Brunei Darussalam | 25.40 | 18.85 | -4.11 (-4.31, -3.91) * |  | 23.01 | 17.13 | -4.03 (-4.30, -3.75) * |  | 22.96 | 16.39 | -5.12 (-5.79, -4.45) |  | 87.49 | 84.92 | -0.88 (-1.56, -0.20) |
| Bulgaria | 66.60 | 46.45 | -4.90 (-5.26, -4.54) * |  | 61.55 | 41.82 | -5.26 (-5.67, -4.84) * |  | 49.94 | 45.70 | -1.19 (-1.74, -0.63) * |  | 50.35 | 62.05 | 3.10 (2.34, 3.86) * |
| Burkina Faso | 4.06 | 2.21 | -8.47 (-11.16, -5.69) * |  | 1.62 | 0.83 | -9.40 (-12.50, -6.18) * |  | 18.45 | 9.48 | -9.17 (-11.56, -6.71) * |  | 594.15 | 434.64 | -4.47 (-6.82, -2.06) * |
| Burundi | 5.01 | 4.53 | -1.19 (-1.57, -0.81) * |  | 4.95 | 4.47 | -1.2 (-1.58, -0.81) * |  | 0.52 | 0.47 | -1.38 (-1.85, -0.90) |  | 6.29 | 7.77 | 3.13 (2.50, 3.76) * |
| Cabo Verde | 0.21 | 0.21 | 0.19 (-0.21, 0.60) * |  | 0.20 | 0.20 | 0.21 (-0.18, 0.60) * |  | 0.06 | 0.05 | -1.20 (-2.15, -0.23) |  | 1.09 | 1.44 | 3.57 (2.65, 4.49) * |
| Cambodia | 144.59 | 103.20 | -4.79 (-5.03, -4.54) * |  | 138.23 | 97.40 | -4.96 (-5.15, -4.76) * |  | 62.91 | 57.21 | -1.56 (-2.87, -0.23) * |  | 63.04 | 77.98 | 2.87 (1.35, 4.41) * |
| Cameroon | 300.10 | 280.85 | -1.00 (-1.43, -0.57) * |  | 290.95 | 271.31 | -1.06 (-1.48, -0.63) * |  | 90.56 | 94.11 | 0.57 (-0.20, 1.35) * |  | 90.47 | 126.58 | 4.93 (3.93, 5.94) * |
| Canada | 81.76 | 64.01 | -3.53 (-3.79, -3.26) |  | 78.51 | 60.94 | -3.64 (-3.87, -3.42) |  | 32.17 | 30.30 | -0.99 (-2.35, 0.40) * |  | 32.58 | 41.72 | 3.45 (1.87, 5.06) * |
| Central African Republic | 0.23 | 0.20 | -2.03 (-3.46, -0.57) * |  | 0.22 | 0.18 | -2.45 (-4.04, -0.83) * |  | 0.13 | 0.15 | 1.83 (0.87, 2.81) |  | 2.47 | 3.94 | 6.76 (5.54, 7.99) * |
| Chad | 0.87 | 0.63 | -4.16 (-6.00, -2.28) * |  | 0.86 | 0.62 | -4.14 (-5.96, -2.29) * |  | 0.09 | 0.05 | -6.74 (-10.51, -2.81) * |  | 2.75 | 2.12 | -1.99 (-5.80, 1.96) * |
| Chile | 2.54 | 1.94 | -3.77 (-4.70, -2.82) * |  | 2.47 | 1.89 | -3.80 (-4.73, -2.86) * |  | 0.55 | 0.45 | -3.40 (-4.68, -2.10) * |  | 10.45 | 11.70 | 1.19 (-0.01, 2.41) |
| China | 69.79 | 46.40 | -5.83 (-6.31, -5.35) * |  | 66.02 | 43.91 | -5.84 (-6.35, -5.32) * |  | 37.31 | 24.51 | -5.80 (-6.09, -5.51) * |  | 37.58 | 33.58 | -1.57 (-1.74, -1.39) * |
| Colombia | 3.21 | 1.97 | -6.62 (-7.15, -6.08) * |  | 2.90 | 1.79 | -6.59 (-7.10, -6.07) * |  | 2.74 | 1.53 | -7.47 (-9.50, -5.39) * |  | 38.06 | 29.22 | -3.15 (-5.13, -1.12) * |
| Comoros | 2.19 | 1.74 | -3.21 (-3.55, -2.87) |  | 2.00 | 1.57 | -3.39 (-3.70, -3.08) * |  | 1.70 | 1.47 | -1.94 (-2.61, -1.27) * |  | 20.38 | 24.21 | 2.58 (1.68, 3.48) * |
| Congo | 1.11 | 0.91 | -2.22 (-3.39, -1.03) * |  | 1.06 | 0.87 | -2.30 (-3.44, -1.14) * |  | 0.45 | 0.39 | -1.19 (-2.87, 0.51) |  | 5.43 | 6.52 | 3.33 (1.77, 4.91) * |
| Costa Rica | 0.33 | 0.28 | -2.19 (-2.69, -1.68) * |  | 0.29 | 0.27 | -1.21 (-1.90, -0.52) * |  | 0.32 | 0.10 | -14.24 (-15.99, -12.46) |  | 6.09 | 2.77 | -10.06 (-11.76, -8.32) * |
| Croatia | 16.46 | 13.37 | -2.71 (-3.18, -2.25) * |  | 15.39 | 12.37 | -2.88 (-3.33, -2.42) * |  | 10.57 | 9.85 | -0.55 (-1.35, 0.26) * |  | 10.66 | 13.52 | 3.90 (3.07, 4.74) |
| Cuba | 0.31 | 0.27 | -2.09 (-2.81, -1.37) * |  | 0.30 | 0.26 | -2.31 (-3.12, -1.49) * |  | 0.11 | 0.10 | 0.75 (-1.85, 3.43) * |  | 3.42 | 4.76 | 5.98 (3.14, 8.9) * |
| Cyprus | 71.65 | 54.93 | -3.93 (-5.09, -2.75) * |  | 66.69 | 50.89 | -4.02 (-5.24, -2.79) * |  | 49.07 | 39.91 | -2.78 (-3.33, -2.22) * |  | 49.25 | 54.28 | 1.52 (0.81, 2.24) * |
| Czechia | 0.26 | 0.23 | -1.47 (-2.10, -0.83) * |  | 0.24 | 0.21 | -1.87 (-2.64, -1.09) * |  | 0.14 | 0.17 | 2.2 (0.71, 3.71) |  | 2.73 | 4.55 | 7.16 (5.43, 8.92) * |
| Cote d'Ivoire | 87.75 | 62.99 | -4.59 (-4.82, -4.37) * |  | 77.98 | 53.82 | -5.1 (-5.41, -4.79) * |  | 96.72 | 90.52 | -1.14 (-2.04, -0.23) * |  | 97.52 | 123.28 | 3.19 (2.08, 4.31) * |
| Democratic Republic of the Congo | 0.30 | 0.23 | -3.01 (-4.14, -1.87) * |  | 0.29 | 0.22 | -3.12 (-4.32, -1.9) * |  | 0.09 | 0.08 | -1.13 (-2.62, 0.38) * |  | 1.62 | 2.07 | 3.56 (1.85, 5.29) * |
| Denmark | 3.43 | 3.49 | 0.50 (0.17, 0.83) * |  | 3.37 | 3.43 | 0.52 (0.18, 0.85) * |  | 0.51 | 0.46 | -1.26 (-1.98, -0.54) |  | 6.14 | 7.61 | 3.26 (2.64, 3.90) * |
| Djibouti | 7.37 | 7.39 | -0.20 (-2.86, 2.53) * |  | 7.28 | 7.29 | -0.22 (-2.88, 2.52) * |  | 0.79 | 0.84 | 0.46 (-1.50, 2.46) * |  | 9.67 | 14.05 | 5.14 (2.89, 7.44) * |
| Dominica | 2.15 | 1.53 | -4.69 (-4.90, -4.47) * |  | 2.05 | 1.46 | -4.77 (-4.95, -4.59) * |  | 0.86 | 0.69 | -3.24 (-4.25, -2.22) * |  | 4.68 | 5.10 | 1.12 (-0.12, 2.37) * |
| Dominican Republic | 6.00 | 4.73 | -3.10 (-3.86, -2.34) |  | 5.29 | 4.17 | -3.09 (-3.79, -2.37) |  | 6.39 | 4.77 | -3.78 (-4.95, -2.59) |  | 76.84 | 78.45 | 0.61 (-0.45, 1.68) * |
| Ecuador | 1.87 | 1.19 | -5.66 (-7.88, -3.40) * |  | 1.04 | 0.74 | -3.92 (-6.00, -1.80) * |  | 6.29 | 3.06 | -9.41 (-11.75, -7.02) * |  | 202.31 | 140.47 | -4.71 (-7.01, -2.35) |
| Egypt | 1.77 | 1.23 | -5.71 (-7.28, -4.11) * |  | 1.56 | 1.11 | -5.58 (-7.33, -3.81) * |  | 1.95 | 1.18 | -6.97 (-7.23, -6.69) * |  | 10.63 | 8.77 | -2.81 (-3.24, -2.38) * |
| El Salvador | 0.47 | 0.37 | -3.28 (-4.31, -2.23) |  | 0.45 | 0.36 | -3.21 (-4.24, -2.17) |  | 0.14 | 0.09 | -5.96 (-7.06, -4.85) |  | 2.64 | 2.42 | -1.24 (-2.20, -0.26) * |
| Equatorial Guinea | 93.58 | 60.90 | -5.88 (-6.18, -5.57) * |  | 88.44 | 56.64 | -6.09 (-6.45, -5.74) * |  | 50.89 | 42.02 | -2.66 (-3.53, -1.78) |  | 51.09 | 56.72 | 1.53 (0.44, 2.63) * |
| Estonia | 0.52 | 0.40 | -3.77 (-5.86, -1.64) * |  | 0.49 | 0.36 | -4.09 (-6.31, -1.82) * |  | 0.27 | 0.26 | -0.48 (-1.48, 0.53) * |  | 5.07 | 6.85 | 4.26 (3.18, 5.36) * |
| Eswatini | 19.13 | 15.58 | -2.91 (-3.02, -2.81) * |  | 19.00 | 15.45 | -2.93 (-3.06, -2.81) * |  | 1.12 | 1.08 | -0.94 (-3.34, 1.51) * |  | 15.42 | 20.68 | 3.75 (1.02, 6.54) * |
| Ethiopia | 0.76 | 0.60 | -3.23 (-4.74, -1.70) * |  | 0.73 | 0.57 | -3.32 (-4.85, -1.77) * |  | 0.27 | 0.23 | -2.22 (-3.32, -1.10) * |  | 5.06 | 6.08 | 2.59 (1.59, 3.59) * |
| Finland | 58.06 | 41.43 | -4.62 (-4.96, -4.28) * |  | 54.81 | 38.71 | -4.75 (-5.07, -4.44) * |  | 32.15 | 26.79 | -2.63 (-3.55, -1.70) |  | 32.53 | 36.68 | 1.66 (0.52, 2.82) * |
| France | 0.34 | 0.31 | -1.25 (-1.49, -1.01) * |  | 0.33 | 0.30 | -1.19 (-1.42, -0.96) * |  | 0.10 | 0.08 | -3.81 (-4.6, -3.01) * |  | 1.92 | 2.11 | 0.81 (0.03, 1.60) * |
| Gabon | 0.66 | 0.53 | -2.92 (-3.26, -2.59) * |  | 0.65 | 0.52 | -3.00 (-3.32, -2.68) * |  | 0.08 | 0.08 | 1.35 (-0.37, 3.10) * |  | 0.97 | 1.39 | 5.89 (3.96, 7.85) * |
| Gambia | 4.85 | 3.84 | -2.75 (-4.87, -0.57) |  | 3.34 | 2.45 | -3.73 (-6.09, -1.31) |  | 11.49 | 9.55 | -2.18 (-3.81, -0.53) * |  | 369.37 | 436.99 | 2.87 (1.26, 4.49) * |
| Georgia | 79.35 | 58.34 | -4.47 (-6.02, -2.90) * |  | 74.85 | 54.85 | -4.53 (-6.11, -2.92) * |  | 44.53 | 34.38 | -3.58 (-4.61, -2.53) * |  | 44.79 | 47.16 | 0.79 (-0.48, 2.08) * |
| Germany | 74.91 | 68.81 | -1.07 (-2.17, 0.04) * |  | 70.63 | 65.16 | -1.02 (-2.16, 0.13) * |  | 42.37 | 36.05 | -1.91 (-2.60, -1.20) * |  | 42.85 | 49.31 | 2.42 (1.63, 3.21) * |
| Ghana | 79.94 | 67.24 | -2.44 (-3.69, -1.18) * |  | 75.57 | 63.82 | -2.41 (-3.71, -1.09) * |  | 43.22 | 33.78 | -3.14 (-3.72, -2.55) * |  | 43.61 | 45.84 | 1.04 (0.35, 1.74) |
| Greece | 41.72 | 32.34 | -3.32 (-3.84, -2.80) * |  | 39.84 | 30.48 | -3.50 (-4.00, -3.00) * |  | 18.61 | 18.36 | 0 (-1.17, 1.19) * |  | 18.86 | 25.05 | 4.33 (2.95, 5.73) * |
| Grenada | 0.74 | 0.51 | -4.84 (-8.04, -1.53) * |  | 0.66 | 0.48 | -4.38 (-7.55, -1.09) * |  | 0.64 | 0.29 | -10.41 (-13.61, -7.09) |  | 12.08 | 7.60 | -6.14 (-9.32, -2.85) * |
| Guatemala | 3.89 | 3.15 | -2.87 (-3.45, -2.27) * |  | 3.64 | 2.96 | -2.85 (-3.45, -2.24) * |  | 2.15 | 1.64 | -3.65 (-4.15, -3.15) * |  | 25.97 | 27.12 | 0.75 (0.18, 1.31) * |
| Guinea | 103.35 | 81.28 | -3.36 (-3.94, -2.77) * |  | 98.44 | 77.87 | -3.30 (-3.93, -2.67) * |  | 48.60 | 33.68 | -4.56 (-5.55, -3.55) * |  | 49.14 | 45.63 | -0.49 (-1.45, 0.47) * |
| Guinea-Bissau | 11.92 | 8.86 | -4.11 (-4.56, -3.67) * |  | 11.72 | 8.71 | -4.11 (-4.56, -3.66) * |  | 1.83 | 1.32 | -4.65 (-5.00, -4.29) * |  | 22.04 | 21.91 | -0.19 (-0.72, 0.34) |
| Guyana | 10.24 | 9.17 | -1.67 (-1.86, -1.47) * |  | 9.64 | 8.60 | -1.72 (-1.92, -1.52) * |  | 5.34 | 4.89 | -1.37 (-1.56, -1.18) * |  | 64.70 | 81.43 | 3.22 (2.90, 3.54) |
| Haiti | 0.26 | 0.23 | -1.38 (-2.28, -0.48) * |  | 0.25 | 0.22 | -1.6 (-2.49, -0.70) * |  | 0.10 | 0.11 | 2.31 (0.89, 3.75) * |  | 1.18 | 1.81 | 7.04 (5.45, 8.65) * |
| Honduras | 18.50 | 14.51 | -3.44 (-3.55, -3.33) * |  | 18.33 | 14.36 | -3.46 (-3.57, -3.36) * |  | 1.48 | 1.30 | -1.88 (-2.86, -0.88) * |  | 17.89 | 21.63 | 2.67 (1.58, 3.77) * |
| Hungary | 0.46 | 0.31 | -5.47 (-7.31, -3.60) * |  | 0.44 | 0.30 | -5.28 (-7.06, -3.46) * |  | 0.19 | 0.08 | -10.82 (-14.11, -7.40) * |  | 6.13 | 3.65 | -6.31 (-9.58, -2.93) * |
| Iceland | 53.38 | 38.16 | -4.60 (-4.87, -4.33) * |  | 52.51 | 37.77 | -4.53 (-4.76, -4.30) * |  | 7.58 | 3.28 | -10.46 (-14.38, -6.36) * |  | 104.85 | 62.86 | -6.17 (-10.19, -1.97) * |
| India | 0.50 | 0.39 | -3.13 (-4.77, -1.45) * |  | 0.48 | 0.38 | -3.01 (-4.65, -1.33) * |  | 0.18 | 0.11 | -6.81 (-8.43, -5.15) * |  | 3.42 | 2.93 | -2.29 (-3.81, -0.74) * |
| Indonesia | 0.27 | 0.22 | -2.84 (-5.00, -0.63) * |  | 0.24 | 0.19 | -3.00 (-5.34, -0.61) * |  | 0.29 | 0.23 | -2.64 (-3.72, -1.55) * |  | 5.48 | 6.17 | 2.08 (1.11, 3.06) * |
| Iran | 47.83 | 36.11 | -4.16 (-4.79, -3.53) * |  | 42.05 | 31.18 | -4.41 (-5.03, -3.79) * |  | 55.71 | 46.89 | -2.63 (-3.38, -1.87) * |  | 211.06 | 241.79 | 1.76 (0.81, 2.71) * |
| Iraq | 5.54 | 3.87 | -4.84 (-5.54, -4.13) * |  | 5.21 | 3.61 | -4.95 (-5.69, -4.19) * |  | 3.14 | 2.46 | -3.48 (-4.10, -2.84) * |  | 17.10 | 18.27 | 0.86 (0.15, 1.58) * |
| Ireland | 1.52 | 1.17 | -3.91 (-5.11, -2.70) * |  | 1.46 | 1.12 | -3.98 (-5.18, -2.77) * |  | 0.53 | 0.46 | -2.48 (-3.69, -1.26) * |  | 2.88 | 3.40 | 1.95 (0.52, 3.4) * |
| Israel | 0.32 | 0.21 | -6.96 (-8.67, -5.22) * |  | 0.30 | 0.20 | -6.34 (-8.09, -4.55) * |  | 0.19 | 0.04 | -20.56 (-22.55, -18.53) * |  | 3.64 | 1.07 | -16.70 (-18.67, -14.68) * |
| Italy | 0.31 | 0.27 | -1.58 (-2.43, -0.73) * |  | 0.29 | 0.25 | -1.54 (-2.41, -0.66) * |  | 0.18 | 0.14 | -3.03 (-3.94, -2.10) * |  | 3.45 | 3.75 | 1.62 (0.66, 2.59) * |
| Jamaica | 0.32 | 0.35 | 2.02 (-0.14, 4.22) |  | 0.31 | 0.33 | 2.02 (-0.19, 4.28) |  | 0.09 | 0.11 | 1.44 (-0.81, 3.75) |  | 1.12 | 1.78 | 6.20 (3.64, 8.83) * |
| Japan | 0.72 | 0.53 | -4.15 (-5.37, -2.92) * |  | 0.64 | 0.48 | -3.87 (-5.01, -2.70) * |  | 0.76 | 0.44 | -6.97 (-8.79, -5.12) * |  | 4.17 | 3.30 | -2.79 (-4.51, -1.04) * |
| Jordan | 0.98 | 0.77 | -3.36 (-4.62, -2.08) * |  | 0.96 | 0.75 | -3.38 (-4.66, -2.09) * |  | 0.16 | 0.13 | -2.90 (-3.78, -2.00) * |  | 2.94 | 3.42 | 1.87 (1.04, 2.70) * |
| Kazakhstan | 85.41 | 65.22 | -3.80 (-4.29, -3.30) * |  | 82.59 | 62.89 | -3.84 (-4.39, -3.29) * |  | 27.91 | 22.90 | -2.50 (-3.86, -1.12) * |  | 28.07 | 31.16 | 1.79 (0.54, 3.05) * |
| Kenya | 11.75 | 6.93 | -7.80 (-9.00, -6.58) * |  | 5.77 | 3.69 | -6.88 (-8.07, -5.68) * |  | 45.25 | 22.20 | -10.03 (-11.33, -8.71) * |  | 1456.52 | 1016.91 | -5.38 (-6.61, -4.14) * |
| Kiribati | 75.50 | 50.35 | -5.70 (-5.85, -5.54) * |  | 73.75 | 49.39 | -5.65 (-5.79, -5.52) * |  | 15.42 | 8.07 | -8.21 (-11.65, -4.63) |  | 213.12 | 154.03 | -3.88 (-7.32, -0.32) * |
| Kuwait | 108.16 | 89.63 | -2.92 (-3.72, -2.12) * |  | 107.49 | 88.97 | -2.94 (-3.72, -2.15) * |  | 5.90 | 5.60 | -1.48 (-4.17, 1.29) * |  | 80.94 | 106.25 | 3.19 (0.22, 6.25) * |
| Kyrgyzstan | 71.86 | 64.79 | -1.45 (-3.30, 0.43) * |  | 65.61 | 55.12 | -2.62 (-4.25, -0.97) * |  | 61.91 | 95.40 | 7.68 (2.61, 13.01) * |  | 62.43 | 130.70 | 12.51 (6.99, 18.32) * |
| Lao People's Democratic Republic | 1.86 | 1.52 | -2.71 (-2.95, -2.47) * |  | 1.84 | 1.50 | -2.72 (-2.97, -2.47) * |  | 0.22 | 0.18 | -2.72 (-3.43, -2.00) * |  | 2.61 | 2.96 | 1.90 (1.26, 2.55) * |
| Latvia | 5.41 | 3.83 | -4.75 (-5.99, -3.49) * |  | 5.21 | 3.70 | -4.73 (-5.97, -3.47) * |  | 1.66 | 1.06 | -6.21 (-7.51, -4.89) * |  | 31.44 | 28.12 | -1.64 (-2.85, -0.42) * |
| Lebanon | 1.71 | 1.19 | -3.85 (-6.85, -0.76) * |  | 1.66 | 1.14 | -3.92 (-7.01, -0.72) * |  | 0.53 | 0.43 | -2.37 (-3.47, -1.26) * |  | 2.90 | 3.23 | 2.01 (0.82, 3.22) * |
| Lesotho | 8.25 | 3.96 | -10.22 (-13.25, -7.07) * |  | 3.75 | 2.29 | -6.82 (-9.54, -4.02) * |  | 34.03 | 11.50 | -14.91 (-18.05, -11.65) * |  | 1095.74 | 525.16 | -10.55 (-13.70, -7.29) * |
| Liberia | 66.68 | 45.00 | -5.52 (-5.70, -5.33) * |  | 65.49 | 44.45 | -5.45 (-5.60, -5.30) * |  | 10.46 | 4.65 | -10.41 (-14.05, -6.62) * |  | 144.83 | 89.08 | -6.16 (-9.85, -2.32) * |
| Libya | 1.01 | 0.80 | -3.19 (-3.4, -2.98) * |  | 0.97 | 0.77 | -3.28 (-3.51, -3.05) * |  | 0.36 | 0.33 | -1.24 (-2.05, -0.42) * |  | 1.95 | 2.45 | 3.35 (2.37, 4.33) * |
| Lithuania | 2.18 | 2.07 | -0.56 (-1.27, 0.15) * |  | 2.16 | 2.04 | -0.54 (-1.27, 0.19) * |  | 0.24 | 0.20 | -2.58 (-3.68, -1.47) * |  | 2.95 | 3.37 | 2.01 (0.99, 3.04) * |
| Luxembourg | 5.67 | 3.62 | -6.19 (-6.67, -5.71) * |  | 5.60 | 3.59 | -6.17 (-6.62, -5.71) * |  | 0.64 | 0.31 | -9.33 (-12.08, -6.5) * |  | 8.93 | 5.91 | -5.14 (-7.89, -2.30) * |
| Madagascar | 57.01 | 45.83 | -3.14 (-3.61, -2.66) * |  | 54.46 | 43.75 | -3.17 (-3.67, -2.66) * |  | 25.23 | 20.55 | -2.48 (-3.36, -1.59) * |  | 25.77 | 28.40 | 1.82 (0.97, 2.68) * |
| Malawi | 214.64 | 166.71 | -3.81 (-4.50, -3.12) * |  | 197.95 | 151.09 | -4.06 (-4.81, -3.30) * |  | 165.21 | 154.15 | -1.15 (-1.44, -0.87) * |  | 166.31 | 210.11 | 3.21 (2.91, 3.50) * |
| Malaysia | 5.36 | 3.54 | -6.29 (-7.38, -5.18) * |  | 3.51 | 2.25 | -6.65 (-7.72, -5.57) * |  | 14.00 | 8.81 | -6.93 (-8.06, -5.79) * |  | 450.26 | 403.71 | -2.12 (-3.38, -0.84) |
| Maldives | 0.22 | 0.17 | -3.66 (-5.11, -2.20) * |  | 0.19 | 0.16 | -3.07 (-4.52, -1.60) * |  | 0.19 | 0.09 | -10.76 (-11.82, -9.69) * |  | 3.52 | 2.26 | -6.47 (-7.43, -5.50) * |
| Mali | 2.13 | 1.68 | -4.04 (-5.24, -2.83) * |  | 1.57 | 1.28 | -3.53 (-4.64, -2.40) * |  | 4.24 | 2.69 | -6.93 (-8.89, -4.93) * |  | 136.31 | 122.94 | -2.15 (-4.06, -0.19) * |
| Malta | 1.46 | 1.63 | 2.14 (1.08, 3.21) * |  | 1.39 | 1.55 | 2.12 (1.08, 3.18) * |  | 0.61 | 0.69 | 2.19 (0.68, 3.73) * |  | 3.30 | 5.12 | 6.86 (5.06, 8.69) * |
| Marshall Islands | 18.81 | 13.63 | -4.49 (-4.84, -4.13) * |  | 18.07 | 12.96 | -4.62 (-4.99, -4.25) * |  | 7.00 | 6.22 | -1.77 (-2.07, -1.46) * |  | 38.20 | 46.23 | 2.66 (2.18, 3.14) * |
| Mauritania | 6.76 | 3.73 | -8.39 (-9.84, -6.91) * |  | 3.06 | 1.40 | -11.14 (-12.78, -9.46) * |  | 27.97 | 16.02 | -7.74 (-9.41, -6.04) * |  | 900.52 | 734.99 | -2.96 (-4.77, -1.11) * |
| Mauritius | 0.59 | 0.48 | -2.80 (-2.97, -2.64) * |  | 0.57 | 0.47 | -2.70 (-2.90, -2.50) * |  | 0.15 | 0.09 | -7.28 (-10.64, -3.79) * |  | 4.82 | 4.03 | -2.43 (-6.09, 1.38) |
| Mexico | 109.87 | 91.94 | -2.46 (-3.01, -1.91) * |  | 105.74 | 86.39 | -2.79 (-3.26, -2.31) * |  | 40.93 | 54.82 | 4.02 (1.47, 6.64) * |  | 41.16 | 74.30 | 8.55 (5.67, 11.52) * |
| Micronesia (Federated States of) | 26.00 | 20.06 | -3.66 (-3.81, -3.50) * |  | 24.62 | 19.53 | -3.34 (-3.70, -2.97) * |  | 12.06 | 4.37 | -12.3 (-17.26, -7.04) |  | 167.12 | 83.74 | -8.13 (-13.18, -2.78) * |
| Mongolia | 1.50 | 1.26 | -2.17 (-2.72, -1.62) * |  | 1.43 | 1.22 | -2.08 (-2.63, -1.53) * |  | 0.54 | 0.33 | -5.46 (-7.76, -3.09) * |  | 17.28 | 15.25 | -0.53 (-3.03, 2.03) * |
| Montenegro | 66.15 | 53.85 | -2.82 (-3.21, -2.43) * |  | 62.25 | 50.59 | -2.85 (-3.26, -2.43) * |  | 38.59 | 32.18 | -2.34 (-2.87, -1.80) * |  | 38.86 | 43.89 | 1.97 (1.49, 2.45) |
| Morocco | 15.94 | 11.38 | -4.59 (-5.24, -3.94) * |  | 12.98 | 8.84 | -5.20 (-6.01, -4.40) * |  | 22.38 | 17.49 | -3.56 (-3.73, -3.40) * |  | 715.91 | 796.96 | 1.43 (1.16, 1.70) * |
| Mozambique | 33.47 | 22.89 | -5.36 (-5.63, -5.08) * |  | 31.64 | 21.65 | -5.36 (-5.65, -5.07) * |  | 18.05 | 12.17 | -5.31 (-6.24, -4.37) * |  | 18.21 | 16.70 | -1.06 (-1.86, -0.25) * |
| Namibia | 0.16 | 0.13 | -2.68 (-3.22, -2.14) * |  | 0.15 | 0.12 | -2.29 (-2.80, -1.78) * |  | 0.06 | 0.02 | -14.64 (-15.85, -13.42) * |  | 1.13 | 0.54 | -10.53 (-11.69, -9.36) * |
| Nauru | 1.07 | 0.88 | -2.29 (-3.35, -1.22) * |  | 1.06 | 0.87 | -2.24 (-3.29, -1.19) * |  | 0.16 | 0.10 | -5.83 (-8.49, -3.10) * |  | 2.18 | 1.82 | -1.39 (-4.29, 1.60) |
| Nepal | 6.80 | 5.12 | -3.86 (-4.32, -3.39) * |  | 6.67 | 5.06 | -3.78 (-4.19, -3.37) * |  | 1.06 | 0.49 | -9.56 (-13.56, -5.38) * |  | 14.58 | 9.22 | -5.3 (-9.33, -1.10) * |
| Netherlands | 111.49 | 91.66 | -2.70 (-3.11, -2.29) * |  | 108.97 | 87.53 | -3.03 (-3.41, -2.65) * |  | 24.91 | 40.76 | 7.36 (4.98, 9.78) * |  | 25.11 | 55.41 | 12.03 (9.37, 14.77) |
| Nicaragua | 2.53 | 2.15 | -2.38 (-2.82, -1.93) * |  | 2.37 | 2.00 | -2.52 (-2.96, -2.09) * |  | 1.37 | 1.29 | -0.86 (-1.52, -0.20) * |  | 16.58 | 21.29 | 3.62 (3.10, 4.16) * |
| Niger | 9.83 | 8.03 | -2.56 (-3.40, -1.70) * |  | 9.60 | 7.86 | -2.54 (-3.36, -1.71) * |  | 1.99 | 1.45 | -3.96 (-6.22, -1.65) * |  | 27.67 | 27.82 | 0.52 (-1.68, 2.78) * |
| Nigeria | 190.31 | 151.20 | -3.39 (-4.06, -2.71) * |  | 171.80 | 134.43 | -3.57 (-4.25, -2.90) * |  | 183.21 | 165.40 | -1.84 (-2.64, -1.02) * |  | 184.49 | 225.38 | 2.49 (1.50, 3.49) * |
| North Macedonia | 71.28 | 50.06 | -4.89 (-5.18, -4.60) * |  | 63.68 | 43.87 | -5.16 (-5.39, -4.92) * |  | 75.21 | 61.12 | -2.83 (-3.6, -2.06) * |  | 75.71 | 83.27 | 1.46 (0.83, 2.09) |
| Norway | 65.95 | 58.97 | -1.47 (-2.06, -0.88) * |  | 62.15 | 55.74 | -1.45 (-2.06, -0.83) * |  | 37.64 | 31.86 | -1.9 (-2.57, -1.23) * |  | 38.00 | 43.26 | 2.34 (1.59, 3.10) * |
| Oman | 55.46 | 44.82 | -3.12 (-4.49, -1.74) * |  | 50.72 | 40.91 | -3.16 (-4.62, -1.69) * |  | 46.86 | 38.62 | -2.73 (-3.18, -2.28) * |  | 47.15 | 52.18 | 1.46 (0.86, 2.07) |
| Pakistan | 4.06 | 3.07 | -3.98 (-4.54, -3.41) * |  | 3.90 | 2.93 | -4.07 (-4.63, -3.50) * |  | 1.42 | 1.19 | -2.43 (-3.18, -1.68) * |  | 17.14 | 19.61 | 2.06 (1.36, 2.76) * |
| Palau | 0.30 | 0.27 | -1.40 (-1.97, -0.82) * |  | 0.28 | 0.26 | -1.30 (-1.88, -0.73) * |  | 0.14 | 0.11 | -3.91 (-4.66, -3.16) |  | 2.66 | 2.85 | 0.65 (-0.04, 1.34) * |
| Panama | 0.48 | 0.40 | -2.16 (-3.29, -1.03) * |  | 0.45 | 0.38 | -2.13 (-3.26, -0.99) * |  | 0.25 | 0.19 | -3.49 (-4.48, -2.49) * |  | 4.75 | 5.03 | 1.16 (0.23, 2.09) * |
| Papua New Guinea | 45.01 | 34.09 | -3.91 (-4.24, -3.57) * |  | 41.14 | 30.52 | -4.19 (-4.56, -3.82) * |  | 37.29 | 33.92 | -1.37 (-1.51, -1.22) |  | 142.34 | 175.51 | 3.01 (2.65, 3.36) * |
| Paraguay | 19.04 | 10.78 | -7.93 (-8.59, -7.25) * |  | 18.93 | 10.69 | -7.94 (-8.62, -7.26) * |  | 1.03 | 0.70 | -5.96 (-7.58, -4.31) |  | 14.28 | 13.27 | -1.61 (-3.46, 0.27) * |
| Peru | 1.78 | 1.23 | -4.72 (-6.52, -2.88) * |  | 1.68 | 1.18 | -4.63 (-6.40, -2.83) * |  | 0.86 | 0.54 | -6.59 (-9.24, -3.88) * |  | 4.69 | 3.99 | -2.37 (-5.32, 0.68) |
| Philippines | 6.55 | 4.53 | -5.35 (-5.79, -4.91) * |  | 6.20 | 4.27 | -5.39 (-5.84, -4.93) * |  | 3.13 | 2.17 | -5.24 (-5.52, -4.96) * |  | 37.49 | 35.65 | -0.85 (-1.13, -0.57) |
| Poland | 9.06 | 5.86 | -5.99 (-6.94, -5.03) * |  | 7.57 | 4.87 | -6.17 (-6.77, -5.56) * |  | 13.27 | 8.49 | -5.53 (-8.57, -2.39) * |  | 159.87 | 140.87 | -1.12 (-4.12, 1.98) |
| Portugal | 31.59 | 25.87 | -2.81 (-2.93, -2.68) * |  | 29.98 | 23.56 | -3.26 (-3.68, -2.84) * |  | 14.10 | 19.45 | 2.72 (-3.71, 9.58) * |  | 195.08 | 371.97 | 7.57 (0.65, 14.96) |
| Qatar | 42.46 | 35.74 | -1.96 (-3.15, -0.76) * |  | 39.13 | 33.40 | -1.81 (-3.10, -0.50) * |  | 29.21 | 19.67 | -4.57 (-6.31, -2.79) |  | 404.64 | 376.00 | -0.09 (-1.8, 1.65) * |
| Republic of Korea | 64.10 | 46.29 | -4.33 (-4.71, -3.95) * |  | 57.88 | 40.83 | -4.63 (-5.04, -4.23) * |  | 59.88 | 51.94 | -1.95 (-2.22, -1.67) * |  | 228.34 | 267.37 | 2.33 (1.94, 2.72) * |
| Republic of Moldova | 1.20 | 0.89 | -3.97 (-5.02, -2.92) * |  | 1.18 | 0.87 | -3.99 (-5.02, -2.95) * |  | 0.19 | 0.14 | -4.45 (-6.15, -2.73) * |  | 6.19 | 6.23 | 0.46 (-1.15, 2.11) * |
| Romania | 1.26 | 0.96 | -3.86 (-4.75, -2.96) * |  | 1.21 | 0.92 | -3.89 (-4.75, -3.01) * |  | 0.39 | 0.29 | -4.02 (-5.58, -2.43) * |  | 7.32 | 7.58 | 0.66 (-0.81, 2.15) |
| Russian Federation | 4.73 | 4.03 | -2.40 (-2.71, -2.08) * |  | 4.70 | 4.00 | -2.42 (-2.72, -2.11) * |  | 0.28 | 0.28 | -0.50 (-2.79, 1.84) * |  | 3.85 | 5.37 | 4.22 (1.64, 6.88) * |
| Rwanda | 5.12 | 4.09 | -2.93 (-3.87, -1.98) * |  | 4.83 | 3.78 | -3.21 (-4.14, -2.27) * |  | 2.64 | 2.69 | 0.44 (-0.86, 1.75) |  | 31.73 | 44.46 | 5.14 (3.62, 6.68) * |
| Saint Kitts and Nevis | 1.77 | 1.40 | -3.52 (-5.12, -1.88) * |  | 1.70 | 1.33 | -3.68 (-5.29, -2.03) * |  | 0.63 | 0.63 | -0.15 (-1.33, 1.04) * |  | 3.43 | 4.67 | 4.32 (3.23, 5.43) * |
| Saint Lucia | 4.54 | 3.38 | -4.03 (-4.64, -3.43) |  | 4.20 | 3.09 | -4.26 (-5.03, -3.48) |  | 2.60 | 2.03 | -2.86 (-5.42, -0.24) * |  | 83.48 | 92.77 | 2.13 (-0.69, 5.03) * |
| Saint Vincent and the Grenadines | 1.23 | 0.88 | -4.61 (-5.77, -3.43) |  | 1.20 | 0.86 | -4.60 (-5.73, -3.45) |  | 0.25 | 0.15 | -6.48 (-8.74, -4.17) * |  | 8.16 | 7.10 | -1.64 (-3.87, 0.65) * |
| Sao Tome and Principe | 7.52 | 4.15 | -8.82 (-10.38, -7.24) * |  | 4.36 | 2.36 | -9.07 (-10.70, -7.42) * |  | 23.88 | 12.27 | -9.76 (-11.23, -8.27) * |  | 768.66 | 562.15 | -5.10 (-6.57, -3.62) |
| Saudi Arabia | 79.44 | 57.83 | -4.38 (-4.65, -4.11) * |  | 75.41 | 53.87 | -4.64 (-4.88, -4.4) * |  | 39.94 | 39.07 | -0.20 (-1.01, 0.62) * |  | 40.27 | 52.91 | 4.08 (3.41, 4.76) |
| Senegal | 11.55 | 7.16 | -6.62 (-6.81, -6.43) * |  | 10.90 | 6.70 | -6.76 (-6.94, -6.58) * |  | 6.16 | 4.32 | -4.64 (-5.19, -4.09) * |  | 33.69 | 32.32 | -0.29 (-0.77, 0.19) * |
| Serbia | 15.55 | 12.03 | -3.71 (-3.88, -3.54) * |  | 15.44 | 11.93 | -3.72 (-3.89, -3.56) * |  | 0.90 | 0.82 | -1.84 (-4.05, 0.41) * |  | 12.83 | 16.17 | 2.89 (0.37, 5.46) |
| Seychelles | 4.65 | 3.77 | -2.72 (-3.23, -2.22) * |  | 4.61 | 3.74 | -2.69 (-3.20, -2.18) * |  | 0.34 | 0.19 | -8.08 (-11.07, -4.98) * |  | 4.68 | 3.57 | -3.68 (-6.70, -0.56) * |
| Sierra Leone | 7.51 | 5.21 | -5.12 (-5.76, -4.47) * |  | 7.17 | 4.96 | -5.15 (-5.76, -4.54) * |  | 3.16 | 2.28 | -4.70 (-6.07, -3.31) * |  | 17.23 | 16.90 | -0.44 (-2.06, 1.21) |
| Singapore | 0.40 | 0.32 | -3.36 (-4.86, -1.84) * |  | 0.37 | 0.29 | -3.29 (-4.81, -1.76) * |  | 0.27 | 0.19 | -5.02 (-6.36, -3.65) * |  | 5.09 | 4.96 | -0.43 (-1.67, 0.83) * |
| Slovakia | 1.20 | 0.79 | -6.04 (-7.87, -4.16) * |  | 1.16 | 0.77 | -5.91 (-7.73, -4.06) * |  | 0.39 | 0.19 | -10.25 (-12.48, -7.96) * |  | 7.29 | 5.00 | -5.86 (-8.06, -3.61) * |
| Slovenia | 0.40 | 0.29 | -4.00 (-5.30, -2.69) * |  | 0.39 | 0.29 | -3.96 (-5.24, -2.66) * |  | 0.02 | 0.01 | -14.46 (-17.32, -11.49) * |  | 0.67 | 0.30 | -10.11 (-12.99, -7.14) * |
| Solomon Islands | 0.45 | 0.36 | -3.22 (-3.90, -2.54) * |  | 0.44 | 0.35 | -3.16 (-3.82, -2.49) * |  | 0.09 | 0.05 | -7.27 (-8.62, -5.90) |  | 2.80 | 2.29 | -2.53 (-3.78, -1.26) * |
| South Africa | 78.97 | 56.56 | -4.57 (-4.85, -4.30) * |  | 75.07 | 53.68 | -4.61 (-4.86, -4.36) * |  | 38.61 | 28.44 | -4.01 (-4.74, -3.28) * |  | 38.49 | 38.48 | 0.27 (-0.35, 0.89) |
| Spain | 55.09 | 46.66 | -2.37 (-3.53, -1.21) * |  | 52.49 | 44.20 | -2.46 (-3.70, -1.22) * |  | 25.75 | 24.29 | -0.66 (-1.28, -0.04) * |  | 25.95 | 33.31 | 3.81 (3.29, 4.33) * |
| Sri Lanka | 1.80 | 1.73 | -0.41 (-1.35, 0.54) * |  | 1.78 | 1.71 | -0.41 (-1.35, 0.55) * |  | 0.16 | 0.15 | -1.61 (-2.51, -0.70) * |  | 1.97 | 2.42 | 2.91 (1.87, 3.95) * |
| Sudan | 22.32 | 17.93 | -3.11 (-3.94, -2.28) * |  | 20.59 | 16.52 | -3.12 (-3.93, -2.30) * |  | 17.04 | 13.85 | -3.12 (-4.26, -1.96) * |  | 17.00 | 18.75 | 1.18 (-0.19, 2.57) |
| Suriname | 1.60 | 1.47 | -1.43 (-3.53, 0.71) |  | 1.52 | 1.40 | -1.46 (-3.57, 0.70) |  | 0.64 | 0.58 | -1.46 (-3.20, 0.32) * |  | 7.65 | 9.61 | 3.15 (1.14, 5.19) * |
| Sweden | 0.77 | 0.71 | -1.18 (-1.95, -0.39) * |  | 0.72 | 0.66 | -1.08 (-1.85, -0.32) * |  | 0.49 | 0.40 | -2.78 (-3.80, -1.75) * |  | 2.67 | 2.99 | 1.65 (0.42, 2.89) |
| Switzerland | 128.25 | 82.85 | -6.15 (-6.90, -5.40) * |  | 103.24 | 66.51 | -6.23 (-6.86, -5.59) * |  | 247.64 | 161.15 | -5.88 (-7.22, -4.53) * |  | 249.95 | 220.69 | -1.70 (-2.93, -0.46) * |
| Syrian Arab Republic | 111.34 | 90.32 | -2.94 (-2.97, -2.91) * |  | 104.34 | 84.78 | -2.93 (-2.99, -2.87) * |  | 69.27 | 54.60 | -3.13 (-3.65, -2.60) * |  | 69.30 | 74.02 | 1.15 (0.74, 1.57) * |
| Tajikistan | 73.41 | 53.77 | -4.22 (-4.73, -3.71) * |  | 69.10 | 50.73 | -4.21 (-4.76, -3.65) * |  | 42.64 | 29.97 | -4.53 (-5.33, -3.71) * |  | 42.81 | 40.87 | -0.26 (-1.00, 0.48) * |
| Thailand | 10.83 | 7.97 | -4.12 (-4.74, -3.50) * |  | 10.06 | 7.49 | -4.00 (-4.48, -3.53) * |  | 6.73 | 4.02 | -6.44 (-9.16, -3.64) * |  | 93.39 | 76.78 | -2.08 (-4.78, 0.69) |
| Timor-Leste | 12.20 | 10.24 | -2.37 (-3.39, -1.33) * |  | 8.68 | 7.04 | -2.72 (-3.63, -1.80) * |  | 26.61 | 21.89 | -2.91 (-4.35, -1.44) * |  | 856.40 | 1002.92 | 2.10 (0.42, 3.81) |
| Togo | 39.67 | 36.76 | -1.03 (-1.16, -0.9) * |  | 38.94 | 36.31 | -0.96 (-1.07, -0.85) * |  | 6.36 | 3.82 | -6.21 (-9.18, -3.14) * |  | 89.27 | 73.87 | -1.82 (-4.76, 1.22) |
| Trinidad and Tobago | 11.25 | 8.87 | -3.59 (-4.29, -2.89) * |  | 7.57 | 5.92 | -3.53 (-4.10, -2.96) * |  | 27.87 | 20.21 | -5.05 (-6.12, -3.97) * |  | 896.24 | 924.72 | -0.16 (-1.42, 1.12) * |
| Tunisia | 1.39 | 1.04 | -4.04 (-4.20, -3.89) * |  | 1.35 | 1.01 | -4.11 (-4.26, -3.96) * |  | 0.36 | 0.31 | -1.93 (-2.39, -1.47) * |  | 1.96 | 2.31 | 2.54 (2.19, 2.88) * |
| Turkey | 1.45 | 0.97 | -5.37 (-5.80, -4.93) * |  | 1.33 | 0.89 | -5.42 (-5.87, -4.97) * |  | 1.15 | 0.81 | -5.06 (-5.9, -4.21) * |  | 6.28 | 6.00 | -0.82 (-1.85, 0.23) |
| Turkmenistan | 1.22 | 1.12 | -1.19 (-1.59, -0.79) * |  | 1.20 | 1.11 | -1.17 (-1.58, -0.77) * |  | 0.15 | 0.13 | -2.61 (-3.31, -1.9) * |  | 1.83 | 2.16 | 2.08 (1.38, 2.79) |
| Uganda | 84.92 | 68.54 | -3.08 (-4.11, -2.03) * |  | 81.48 | 64.53 | -3.34 (-4.32, -2.34) * |  | 34.06 | 39.51 | 2.04 (-0.34, 4.48) |  | 34.12 | 53.57 | 6.54 (3.87, 9.28) * |
| Ukraine | 6.30 | 6.79 | 2.08 (-1.58, 5.88) |  | 3.34 | 3.29 | 1.09 (-2.66, 4.99) |  | 22.39 | 24.03 | 1.68 (-2.23, 5.75) |  | 721.33 | 1097.32 | 6.87 (2.64, 11.28) * |
| United Arab Emirates | 103.33 | 79.21 | -3.92 (-4.83, -3.00) * |  | 97.72 | 73.41 | -4.20 (-5.12, -3.28) * |  | 55.53 | 57.23 | 0.35 (-0.74, 1.45) * |  | 55.84 | 77.76 | 4.76 (3.43, 6.10) * |
| United Kingdom | 0.18 | 0.18 | -0.05 (-0.30, 0.19) * |  | 0.18 | 0.17 | -0.07 (-0.32, 0.18) * |  | 0.05 | 0.05 | -0.35 (-0.96, 0.26) * |  | 0.97 | 1.33 | 4.50 (3.99, 5.01) * |
| United Republic of Tanzania | 1.27 | 1.13 | -2.13 (-3.61, -0.64) * |  | 1.26 | 1.12 | -2.16 (-3.61, -0.68) * |  | 0.07 | 0.08 | 0.21 (-3.75, 4.33) |  | 1.40 | 2.05 | 4.99 (0.65, 9.50) * |
| United States of America | 12.27 | 8.54 | -5.10 (-5.29, -4.90) |  | 6.23 | 4.79 | -3.67 (-3.98, -3.36) |  | 45.68 | 25.74 | -8.02 (-8.29, -7.75) |  | 1470.52 | 1183.05 | -3.22 (-3.49, -2.95) * |
| Uruguay | 2.15 | 2.09 | -0.23 (-0.93, 0.48) * |  | 2.12 | 2.06 | -0.22 (-0.94, 0.49) * |  | 0.26 | 0.24 | -1.20 (-1.68, -0.72) |  | 3.14 | 3.96 | 3.31 (2.72, 3.91) * |
| Uzbekistan | 31.40 | 22.26 | -4.85 (-4.97, -4.72) * |  | 27.48 | 20.15 | -4.48 (-4.96, -3.99) * |  | 34.41 | 17.70 | -8.47 (-11.31, -5.54) * |  | 476.96 | 338.68 | -4.16 (-6.98, -1.25) * |
| Vanuatu | 25.10 | 21.88 | -1.92 (-2.24, -1.60) * |  | 25.01 | 21.77 | -1.93 (-2.24, -1.62) * |  | 0.82 | 0.88 | 0.17 (-3.04, 3.48) |  | 11.25 | 16.68 | 4.95 (1.47, 8.56) * |
| Viet Nam | 6.92 | 6.92 | 0.18 (-0.64, 1.00) * |  | 6.57 | 6.56 | 0.16 (-0.60, 0.93) * |  | 3.38 | 3.29 | 0.22 (-1.70, 2.17) * |  | 18.40 | 24.36 | 4.67 (2.86, 6.51) * |
| Yemen | 61.70 | 40.12 | -5.92 (-6.19, -5.65) |  | 58.28 | 37.80 | -5.97 (-6.25, -5.69) |  | 33.82 | 22.91 | -5.15 (-6.64, -3.63) |  | 34.13 | 31.06 | -1.05 (-2.45, 0.37) * |
| Zambia | 125.86 | 81.58 | -6.00 (-6.22, -5.79) * |  | 120.02 | 75.29 | -6.45 (-6.73, -6.17) * |  | 57.73 | 62.09 | 0.97 (-0.59, 2.56) |  | 58.29 | 84.53 | 5.35 (3.51, 7.22) * |
| Zimbabwe | 167.06 | 121.24 | -4.48 (-4.77, -4.18) * |  | 151.64 | 108.91 | -4.63 (-4.99, -4.27) * |  | 152.67 | 121.65 | -3.08 (-3.62, -2.54) * |  | 154.85 | 165.92 | 1.10 (0.39, 1.81) * |

Countries were arranged in alphabetical order.

TB: tuberculosis; DS-TB: drug-susceptible tuberculosis; MDR-TB: multidrug-resistant tuberculosis; XDR-TB: extensively drug-resistant tuberculosis.

a: per 100 000 population; b: per 1000 000 population; c: per 100 000 000 population; CI: confidence interval.

# Table S7: The number of countries (n, %) and the median EAPC (%) with significant change of incidence, prevalence and mortality of tuberculosis from 2012 to 2019

|  | **Total TB** | | |  | **DS-TB** | | |  | **MDR-TB** | | |  | **XDR-TB** | | |
| --- | --- | --- | --- | --- | --- | --- | --- | --- | --- | --- | --- | --- | --- | --- | --- |
|  | Median  (IQR) | Upward  (n, %) | Downward  (n, %) |  | Median  (IQR) | Upward  (n, %) | Downward  (n, %) |  | Median  (IQR) | Upward  (n, %) | Downward  (n, %) |  | Median  (IQR) | Upward  (n, %) | Downward  (n, %) |
| **Incidence** | | | | | | | | | | | | | | | |
| **Total** | **-2.21 (1.63)** | **3 (1.9)** | **158 (98.1)** |  | **-2.27 (1.62)** | **3 (1.8)** | **160 (98.2)** |  | **-2.88 (4.09)** | **29 (22.5)** | **100 (77.5)** |  | **3.84 (3.74)** | **124 (84.9)** | **22 (15.1)** |
| **WHO region** |  |  |  |  |  |  |  |  |  |  |  |  |  |  |  |
| Africa | -2.00 (0.80) | 1 (2.5) | 39 (97.5) |  | -2.06 (0.81) | 1 (2.4) | 40 (97.6) |  | -1.78 (4.68) | 16 (43.2) | 21 (56.8) |  | 4.50 (3.94) | 38 (97.4) | 1 (2.6) |
| Americas | -1.94 (1.64) | 0 (0) | 29 (100.0) |  | -2.00 (1.11) | 0 (0) | 29 (100.0) |  | -1.69 (5.30) | 6 (42.9) | 8 (57.1) |  | 4.67 (2.99) | 31 (96.9) | 1 (3.1) |
| Eastern Mediterranean | -2.97 (0.92) | 0 (0) | 20 (100.0) |  | -2.96 (0.96) | 0 (0) | 20 (100.0) |  | -2.41 (3.01) | 1 (6.3) | 15 (93.8) |  | 3.34 (1.21) | 15 (93.8) | 1 (6.3) |
| Europe | -2.82 (1.43) | 0 (0) | 46 (100.0) |  | -2.83 (1.57) | 0 (0) | 47 (100.0) |  | -5.21 (4.82) | 4 (8.9) | 41 (91.1) |  | 1.38 (6.85) | 21 (55.3) | 17 (44.7) |
| South-East Asia | -2.61 (1.84) | 1 (12.5) | 7 (87.5) |  | -2.72 (1.90) | 1 (12.5) | 7 (87.5) |  | -4.02 (3.66) | 0 (0) | 6 (100.0) |  | 4.11 (2.64) | 5 (83.3) | 1 (16.7) |
| Western Pacific | -1.49 (1.49) | 1 (5.6) | 17 (94.4) |  | -1.69 (1.36) | 1 (5.6) | 17 (94.4) |  | -4.18 (3.87) | 2 (18.2) | 9 (81.8) |  | 5.66 (2.80) | 14 (93.3) | 1 (6.7) |
| **Income level** |  |  |  |  |  |  |  |  |  |  |  |  |  |  |  |
| Low | -1.73 (0.82) | 0 (0) | 24 (100.0) |  | -1.76 (0.78) | 0 (0) | 24 (100.0) |  | -2.18 (4.71) | 9 (39.1) | 14 (60.9) |  | 3.74 (4.43) | 30 (68.2) | 14 (31.8) |
| Lower-middle | -2.21 (1.64) | 2 (4.7) | 41 (95.3) |  | -2.19 (1.41) | 2 (4.4) | 43 (95.6) |  | -2.13 (3.77) | 8 (26.7) | 22 (73.3) |  | 4.37 (2.93) | 22 (100.0) | 0 (0) |
| Upper-middle | -2.35 (1.47) | 1 (2.3) | 43 (97.7) |  | -2.36 (1.33) | 1 (2.3) | 43 (97.7) |  | -2.89 (6.07) | 7 (21.2) | 26 (78.8) |  | 4.13 (3.17) | 40 (100.0) | 0 (0) |
| High | -2.82 (1.80) | 0 (0) | 50 (100.0) |  | -2.83 (1.81) | 0 (0) | 50 (100.0) |  | -3.69 (4.70) | 5 (11.6) | 38 (88.4) |  | 2.95 (6.08) | 32 (80.0) | 8 (20.0) |
| **Prevalence** | | | | | | | | | | | | | | | |
| **Total** | **-2.31 (1.25)** | **1 (0.6)** | **169 (99.4)** |  | **-2.83 (1.74)** | **1 (0.6)** | **166 (99.4)** |  | **-2.95 (4.44)** | **30 (21.6)** | **109 (78.4)** |  | **3.51 (4.02)** | **115 (82.7)** | **24 (17.3)** |
| **WHO region** |  |  |  |  |  |  |  |  |  |  |  |  |  |  |  |
| Africa | -2.93 (1.26) | 0 (0) | 42 (100.0) |  | -2.15 (0.85) | 1 (2.4) | 41 (97.6) |  | -1.43 (4.94) | 17 (43.6) | 22 (56.4) |  | 4.61 (4.29) | 38 (97.4) | 1 (2.6) |
| Americas | -1.94 (0.55) | 0 (0) | 34 (100.0) |  | -2.69 (1.23) | 0 (0) | 32 (100.0) |  | -1.15 (4.76) | 6 (33.3) | 12 (66.7) |  | 4.36 (2.92) | 30 (96.8) | 1 (3.2) |
| Eastern Mediterranean | -2.57 (0.86) | 0 (0) | 20 (100.0) |  | -3.76 (1.31) | 0 (0) | 20 (100.0) |  | -2.94 (3.23) | 1 (5.9) | 16 (94.1) |  | 2.79 (1.19) | 12 (85.7) | 2 (14.3) |
| Europe | -2.35 (1.06) | 0 (0) | 50 (100.0) |  | -3.59 (1.18) | 0 (0) | 49 (100.0) |  | -5.54 (4.59) | 2 (4.3) | 44 (95.7) |  | -0.31 (6.97) | 17 (48.6) | 18 (51.4) |
| South-East Asia | -0.34 (2.43) | 0 (0) | 7 (100.0) |  | -2.74 (1.44) | 0 (0) | 7 (100.0) |  | -4.26 (4.52) | 1 (14.3) | 6 (85.7) |  | 3.88 (0.73) | 5 (100.0) | 0 (0) |
| Western Pacific | -1.14 (1.79) | 1 (5.9) | 16 (94.1) |  | -1.99 (1.59) | 0 (0) | 17 (100.0) |  | -3.27 (4.79) | 3 (25.0) | 9 (75.0) |  | 4.77 (3.06) | 13 (86.7) | 2 (13.3) |
| **Income level** |  |  |  |  |  |  |  |  |  |  |  |  |  |  |  |
| Low | -2.85 (1.23) | 0 (0) | 24 (100.0) |  | -2.11 (0.95) | 1 (4.3) | 22 (95.7) |  | -2.42 (5.23) | 10 (41.7) | 14 (58.3) |  | 3.76 (4.59) | 27 (65.9) | 14 (34.1) |
| Lower-middle | -2.54 (1.79) | 1 (2.2) | 44 (97.8) |  | -2.52 (2.21) | 0 (0) | 44 (100.0) |  | -2.17 (4.94) | 10 (28.6) | 25 (71.4) |  | 4.38 (3.00) | 20 (95.2) | 1 (4.8) |
| Upper-middle | -2.26 (1.10) | 0 (0) | 48 (100.0) |  | -3.05 (1.42) | 0 (0) | 45 (100.0) |  | -3.17 (6.20) | 7 (18.9) | 30 (81.1) |  | 3.51 (3.73) | 38 (97.4) | 1 (2.6) |
| High | -1.88 (1.02) | 0 (0) | 53 (100.0) |  | -3.47 (1.71) | 0 (0) | 55 (100.0) |  | -4.51 (4.56) | 3 (7.0) | 40 (93.0) |  | 2.40 (6.14) | 30 (78.9) | 8 (21.1) |
| **Mortality** | | | | | | | | | | | | | | | |
| **Total** | **-3.77 (1.99)** | **2 (1.2)** | **161 (98.8)** |  | **-3.68 (1.97)** | **2 (1.2)** | **163 (98.8)** |  | **-3.94 (4.51)** | **8 (5.5)** | **138 (94.5)** |  | **2.01 (5.71)** | **96 (68.1)** | **45 (31.9)** |
| **WHO region** |  |  |  |  |  |  |  |  |  |  |  |  |  |  |  |
| Africa | -3.53 (1.88) | 0 (0) | 43 (100.0) |  | -3.61 (1.89) | 0 (0) | 44 (100.0) |  | -2.83 (2.49) | 3 (8.6) | 32 (91.4) |  | 2.87 (3.02) | 31 (88.6) | 4 (11.4) |
| Americas | -2.89 (1.65) | 1 (4.2) | 23 (95.8) |  | -2.92 (1.81) | 1 (4.2) | 23 (95.8) |  | -2.61 (2.29) | 0 (0) | 25 (100.0) |  | 3.18 (1.79) | 16 (57.1) | 2 (7.1) |
| Eastern Mediterranean | -4.33 (1.09) | 1 (5.3) | 18 (94.7) |  | -4.62 (1.24) | 1 (5.3) | 18 (94.7) |  | -3.13 (4.34) | 1 (5.6) | 17 (94.4) |  | 2.01 (4.27) | 12 (70.6) | 5 (29.4) |
| Europe | -3.66 (2.09) | 0 (0) | 49 (100.0) |  | -3.53 (1.81) | 0 (0) | 49 (100.0) |  | -6.73 (5.15) | 3 (6.5) | 43 (93.5) |  | -2.41 (7.10) | 15 (39.5) | 23 (60.5) |
| South-East Asia | -4.12 (0.57) | 0 (0) | 9 (100.0) |  | -4.19 (0.53) | 0 (0) | 9 (100.0) |  | -6.21 (6.70) | 0 (0) | 9 (100.0) |  | -0.88 (7.31) | 3 (42.9) | 4 (57.1) |
| Western Pacific | -3.66 (2.50) | 0 (0) | 19 (100.0) |  | -3.36 (2.76) | 0 (0) | 20 (100.0) |  | -6.21 (4.50) | 1 (7.7) | 12 (92.3) |  | 1.65 (7.82) | 9 (56.3) | 7 (43.8) |
| **Income level** |  |  |  |  |  |  |  |  |  |  |  |  |  |  |  |
| Low | -3.14 (1.86) | 0 (0) | 23 (100.0) |  | -3.17 (1.64) | 0 (0) | 23 (100.0) |  | -2.57 (1.49) | 2 (10.0 | 18 (90.0 |  | 2.49 (2.85) | 24 (55.8) | 19 (44.2) |
| Lower-middle | -4.16 (2.14) | 0 (0) | 45 (100.0) |  | -4.16 (2.13) | 0 (0) | 46 (100.0) |  | -3.40 (4.40) | 1 (2.8) | 35 (97.2) |  | 2.48 (4.71) | 20 (95.2) | 1 (4.8) |
| Upper-middle | -3.68 (1.89) | 2 (4.5) | 42 (95.5) |  | -3.70 (2.30) | 2 (4.5) | 42 (95.5) |  | -4.90 (5.09) | 1 (2.3) | 42 (97.7) |  | 1.95 (6.47) | 28 (70.0) | 12 (30.0) |
| High | -3.52 (2.30) | 0 (0) | 51 (100.0) |  | -3.31 (1.98) | 0 (0) | 52 (100.0) |  | -5.24 (4.22) | 4 (8.5) | 43 (91.5) |  | 1.16 (7.00) | 24 (64.9) | 13 (35.1) |

TB: tuberculosis; DS-TB: drug-susceptible tuberculosis; MDR-TB: multidrug-resistant tuberculosis; XDR-TB: extensively drug-resistant tuberculosis.

# Table S8: The number of countries (n, %) and the median EAPC (%) with significant change of urbanization level from 2012 to 2019

|  | **Urbanization score** | | |
| --- | --- | --- | --- |
|  | Median  (IQR) | Upward  (n, %) | Downward  (n, %) |
| **Total** | **3.43 (0.88)** | **82 (100.0)** | **0 (0)** |
| **WHO region** |  |  |  |
| Africa | 3.58 (0.60) | 21 (100.0) | 0 (0) |
| Americas | 3.21 (0.45) | 17 (100.0) | 0 (0) |
| Eastern Mediterranean | 4.00 (0.19) | 3 (100.0) | 0 (0) |
| Europe | 3.25 (1.03) | 23 (100.0) | 0 (0) |
| South-East Asia | 4.03 (1.12) | 8 (100.0) | 0 (0) |
| Western Pacific | 3.46 (0.76) | 10 (100.0) | 0 (0) |
| **Income level** |  |  |  |
| Low | 3.83 (0.75) | 12 (100.0) | 0 (0) |
| Lower-middle | 3.83 (0.69) | 25 (100.0) | 0 (0) |
| Upper-middle | 3.12 (0.43) | 23 (100.0) | 0 (0) |
| High | 3.55 (0.93) | 22 (100.0) | 0 (0) |

# Table S9: Results of the fixed-effects models on influence factors associated with burden of tuberculosis

| **Factors** | **Total TB (IRR, 95% CI)** | | |  | **DS-TB (RR, 95% CI)** | | |
| --- | --- | --- | --- | --- | --- | --- | --- |
|  | **Incidence** | **Prevalence** | **Mortality** |  | **Incidence** | **Prevalence** | **Mortality** |
| **Urbanization score** | **1.000 (0.998,1.002)** | **1.001 (0.999,1.002)** | **0.999 (0.996,1.001)** |  | **1.001 (0.999,1.002)** | **0.999 (0.998,1.001)** | **0.999 (0.996,1.002)** |
| PM_2.5_ | 1.003 (1.001,1.004) * | 1.001 (0.9999,1.002) | 1.007 (1.005,1.010) * |  | 1.002 (1.001,1.004) * | 1.003 (1.001,1.005) * | 1.007 (1.004,1.009) * |
| GNI per capita | 0.988 (0.969,1.007) | 1.015 (1.002,1.028) * | 0.994 (0.968,1.020) |  | 0.990 (0.971,1.009) | 1.009 (0.990,1.029) | 0.997 (0.971,1.024) |
| UHC index | 1.000 (0.998,1.002) | 0.999 (0.998,1.000) | 0.993 (0.991,0.996) * |  | 1.000 (0.998,1.002) | 1.002 (1.000,1.004) * | 0.993 (0.991,0.996) * |
| Older adults | 0.982 (0.974,0.990) * | 1.010 (1.004,1.015) * | 0.982 (0.971,0.993) * |  | 0.983 (0.975,0.990) * | 0.983 (0.976,0.991) * | 0.983 (0.972,0.994) * |
| Population density | 0.951 (0.751,1.204) | 0.913 (0.782,1.065) | 0.465 (0.337,0.640) * |  | 0.984 (0.780,1.241) | 1.136 (0.899,1.435) | 0.484 (0.351,0.667) * |
| Wind speed | 1.001 (0.994,1.009) | 1.0004 (0.996,1.005) | 1.009 (0.999,1.019) |  | 1.001 (0.994,1.008) | 1.007 (0.999,1.014) | 1.010 (0.9996,1.020) |
| Relative humidity | 1.000 (0.999,1.001) | 1.000 (0.9998,1.001) | 1.000 (0.9996,1.001) |  | 1.000 (0.999,1.001) | 1.000 (0.9997,1.001) | 1.000 (0.9996,1.001) |
| Temperature | 0.999 (0.996,1.001) | 0.999 (0.998,1.001) | 1.001 (0.998,1.005) |  | 0.999 (0.996,1.001) | 0.999 (0.996,1.002) | 1.001 (0.998,1.005) |
|  | **MDR-TB (RR, 95% CI)** | | |  | **XDR-TB (RR, 95% CI)** | | |
|  | **Incidence** | **Prevalence** | **Mortality** |  | **Incidence** | **Prevalence** | **Mortality** |
| **Urbanization score** | **0.990 (0.985,0.996) *** | **0.989 (0.984,0.994) *** | **0.993 (0.988,0.998) *** |  | **0.991 (0.986,0.996) *** | **0.990 (0.985,0.995) *** | **0.993 (0.988,0.998) *** |
| PM_2.5_ | 1.006 (1.002,1.011) * | 1.007 (1.002,1.012) * | 1.010 (1.005,1.015) * |  | 1.005 (1.000,1.010) * | 1.005 (1.001,1.010) * | 1.009 (1.004,1.013) * |
| GNI per capita | 0.915 (0.865,0.967) * | 0.931 (0.881,0.983) * | 0.919 (0.872,0.969) * |  | 0.915 (0.868,0.965) * | 0.932 (0.885,0.981) * | 0.918 (0.872,0.967) * |
| UHC index | 1.006 (1.001,1.011) * | 1.008 (1.003,1.013) * | 0.996 (0.991,1.000) |  | 1.005 (1.000,1.010) * | 1.007 (1.003,1.012) * | 0.995 (0.990,0.9999) * |
| Older adults | 0.934 (0.913,0.956) * | 0.935 (0.914,0.956) * | 0.944 (0.923,0.965) * |  | 0.938 (0.918,0.959) * | 0.938 (0.918,0.959) * | 0.947 (0.926,0.968) * |
| Population density | 1.157 (0.587,2.282) | 1.075 (0.554,2.087) | 0.964 (0.508,1.829) |  | 0.902 (0.472,1.721) | 0.832 (0.443,1.564) | 0.810 (0.430,1.526) |
| Wind speed | 0.999 (0.979,1.020) | 1.001 (0.981,1.022) | 1.008 (0.988,1.028) |  | 0.999 (0.979,1.019) | 1.001 (0.982,1.021) | 1.007 (0.987,1.027) |
| Relative humidity | 1.000 (0.998,1.002) | 1.000 (0.998,1.002) | 1.000 (0.998,1.002) |  | 1.000 (0.998,1.002) | 1.000 (0.998,1.002) | 0.9999 (0.998,1.002) |
| Temperature | 1.003 (0.995,1.011) | 1.003 (0.995,1.011) | 1.003 (0.995,1.010) |  | 1.002 (0.994,1.010) | 1.003 (0.995,1.010) | 1.003 (0.995,1.010) |

RR: rate ratio; CI: confidence interval; PM_2.5_: fine particulate matter; GNI: gross national income, the GNI per capita and population density were in log scale. TB: tuberculosis; DS-TB: drug sensitive tuberculosis; MDR-TB: multidrug-resistant tuberculosis excluding extensively drug-resistant tuberculosis; XDR-TB: extensively drug-resistant tuberculosis. **P*<0.05.

# Table S10: Results of sensitivity analyses on the association between urbanization level and burden of tuberculosis

| **Factors** | **Total TB (RR, 95% CI)** | | | |  | | **DS-TB (RR, 95% CI)** | | |
| --- | --- | --- | --- | --- | --- | --- | --- | --- | --- |
|  | **Incidence** | **Prevalence** | **Mortality** |  | | **Incidence** | | **Prevalence** | **Mortality** |
| **Sensitivity analysis 1** | 1.0004 (0.998, 1.002) | 1.0003 (0.999, 1.002) | 0.997 (0.994, 0.9998) * |  | | 1.0002 (0.998, 1.002) | | 0.999 (0.997, 1.001) | 0.998 (0.995, 1.0003) |
| **Sensitivity analysis 2** | 1.0001 (0.998, 1.002) | 1.001 (0.999, 1.002) | 1.001 (0.998, 1.003) |  | | 1.001 (0.999, 1.003) | | 1.0001 (0.998, 1.002) | 1.0005 (0.998, 1.003) |
| **Sensitivity analysis 3** | 1.001 (0.998, 1.003) | 1.000 (0.999, 1.001) | 0.998 (0.995, 1.0004) |  | | 1.001 (0.998, 1.003) | | 0.999 (0.997, 1.001) | 0.998 (0.995, 1.0005) |
|  |  |  |  |  | |  | |  |  |
| **Factors** | **MDR-TB (RR, 95% CI)** | | | |  | | **XDR-TB (RR, 95% CI)** | | |
|  | **Incidence** | **Prevalence** | **Mortality** |  | | **Incidence** | | **Prevalence** | **Mortality** |
| **Sensitivity analysis 1** | 0.994 (0.988, 0.9999) * | 0.993 (0.987, 0.999) * | 0.995 (0.990, 0.9998) * |  | | 0.994 (0.988, 0.9998) * | | 0.994 (0.988, 0.9997) * | 0.994 (0.989, 0.999) * |
| **Sensitivity analysis 2** | 0.989 (0.981, 0.998) * | 0.989 (0.980, 0.997) * | 0.993 (0.986, 0.9999) * |  | | 0.990 (0.982, 0.998) * | | 0.990 (0.981, 0.998) * | 0.993 (0.985, 0.9999) * |
| **Sensitivity analysis 3** | 0.993 (0.986, 0.999) * | 0.991 (0.985, 0.997) * | 0.989 (0.983, 0.995) * |  | | 0.993 (0.987, 0.999) * | | 0.991 (0.985, 0.997) * | 0.989 (0.983, 0.995) * |

RR: rate ratio; CI: confidence interval; PM_2.5_: fine particulate matter; GNI: gross national income, the GNI per capita and population density were in log scale. TB: tuberculosis; DS-TB: drug sensitive tuberculosis; MDR-TB: multidrug-resistant tuberculosis excluding extensively drug-resistant tuberculosis; XDR-TB: extensively drug-resistant tuberculosis. **P*<0.05.

In sensitivity analysis 1, we used lower uncertain interval of estimated data from GBD for the burden of tuberculosis.

In sensitivity analysis 2, we used upper uncertain interval of estimated data from GBD for the burden of tuberculosis.

In sensitivity analysis 3, we used socioeconomic development index to replace universal health coverage index in the main models.

# Table S11: Lag effects of urbanization level on burden of tuberculosis in 178 countries and territories

| **Lag years** | **Total TB**  **(RR, 95% CI)** | |  | **DS-TB**  **(RR, 95% CI)** | |  | **MDR-TB**  **(RR, 95% CI)** | |  | **XDR-TB**  **(RR, 95% CI)** |
| --- | --- | --- | --- | --- | --- | --- | --- | --- | --- | --- |
| Incidence |  | |  |  | |  |  | |  |  |
| 0 | 1.000 (0.998, 1.002) | |  | 1.001 (0.999, 1.002) | |  | **0.990 (0.985, 0.996) *** | |  | **0.991 (0.986, 0.996) *** |
| 1 | 1.001 (0.9996, 1.003) | |  | 1.001 (0.9998, 1.003) | |  | **0.994 (0.990, 0.999) *** | |  | **0.995 (0.991, 0.999) *** |
| 2 | 1.001 (0.9999, 1.003) | |  | 1.001 (0.99998, 1.003) | |  | 0.999 (0.995, 1.003) | |  | 0.999 (0.996, 1.003) |
| 3 | 1.001 (0.999, 1.002) | |  | 1.001 (0.999, 1.002) | |  | 1.003 (0.9998, 1.006) | |  | 1.003 (0.99996, 1.006) |
| 4 | 1.000 (0.999, 1.002) | |  | 1.000 (0.998, 1.002) | |  | 1.003 (0.9997, 1.006) | |  | 1.003 (0.9997, 1.006) |
| 5 | 0.999 (0.997, 1.001) | |  | 0.999 (0.997, 1.001) | |  | 1.001 (0.999, 1.004) | |  | 1.001 (0.999, 1.004) |
| 6 | 1.004 (1.0003, 1.007) * | |  | 1.004 (1.000, 1.007) * | |  | 1.004 (0.999, 1.008) | |  | 1.004 (0.9996, 1.008) |
|  |  |  |  |  |  |  |  |  |  |  |
| Prevalence |  |  |  |  |  |  |  |  |  |  |
| 0 | 1.001 (0.999, 1.002) | |  | 0.999 (0.998, 1.001) | |  | **0.989 (0.984, 0.994) *** | |  | **0.99 (0.985, 0.995) *** |
| 1 | 1.001 (0.9997, 1.002) | |  | 1.001 (0.9997, 1.003) | |  | **0.994 (0.990, 0.998) *** | |  | **0.995 (0.991, 0.999) *** |
| 2 | 1.001 (0.999, 1.002) | |  | 1.002 (1.0002, 1.003) * | |  | 0.999 (0.996, 1.003) | |  | 0.9995 (0.996, 1.003) |
| 3 | 1.000 (0.999, 1.001) | |  | 1.001 (0.999, 1.002) | |  | 1.003 (0.9999, 1.006) * | |  | 1.003 (1.00003, 1.006) * |
| 4 | 1.000 (0.999, 1.001) | |  | 1.001 (0.999, 1.003) | |  | 1.003 (1.001, 1.006) * | |  | 1.003 (1.001, 1.006) * |
| 5 | 1.000 (0.999, 1.001) | |  | 0.999 (0.997, 1.001) | |  | 1.001 (0.999, 1.004) | |  | 1.001 (0.999, 1.004) |
| 6 | 1.000 (0.999, 1.002) | |  | 1.003 (0.999, 1.007) | |  | 1.003 (0.998, 1.008) | |  | 1.003 (0.999, 1.008) |
|  |  |  |  |  |  |  |  |  |  |  |
| Mortality |  |  |  |  |  |  |  |  |  |  |
| 0 | 0.999 (0.996, 1.001) | |  | 0.999 (0.996, 1.002) | |  | **0.993 (0.988, 0.998) *** | |  | **0.993 (0.988, 0.998) *** |
| 1 | 0.999 (0.997, 1.002) | |  | 0.999 (0.997, 1.002) | |  | **0.995 (0.991, 0.999) *** | |  | **0.995 (0.991, 0.9996) *** |
| 2 | 0.999 (0.997, 1.001) | |  | 0.999 (0.997, 1.001) | |  | 0.998 (0.994, 1.002) | |  | 0.998 (0.995, 1.002) |
| 3 | 0.998 (0.996, 0.9997) * | |  | 0.998 (0.996, 0.9997) * | |  | 0.9996 (0.996, 1.003) | |  | 0.9996 (0.996, 1.003) |
| 4 | 0.999 (0.997, 1.001) | |  | 0.999 (0.997, 1.001) | |  | 1.001 (0.998, 1.004) | |  | 1.001 (0.998, 1.004) |
| 5 | 0.997 (0.995, 0.999) * | |  | 0.997 (0.995, 0.999) * | |  | 0.9995 (0.997, 1.002) | |  | 0.9995 (0.997, 1.002) |
| 6 | 0.999 (0.996, 1.001) | |  | 0.999 (0.996, 1.001) | |  | 0.999 (0.996, 1.002) | |  | 0.999 (0.996, 1.002) |

RR: rate ratio; CI: confidence interval; TB: tuberculosis; DS-TB: drug sensitive tuberculosis; MDR-TB: multidrug-resistant tuberculosis excluding extensively drug-resistant tuberculosis; XDR-TB: extensively drug-resistant tuberculosis. **P*<0.05.

# Figure S1: Diagram of data preparation and analysis procedure


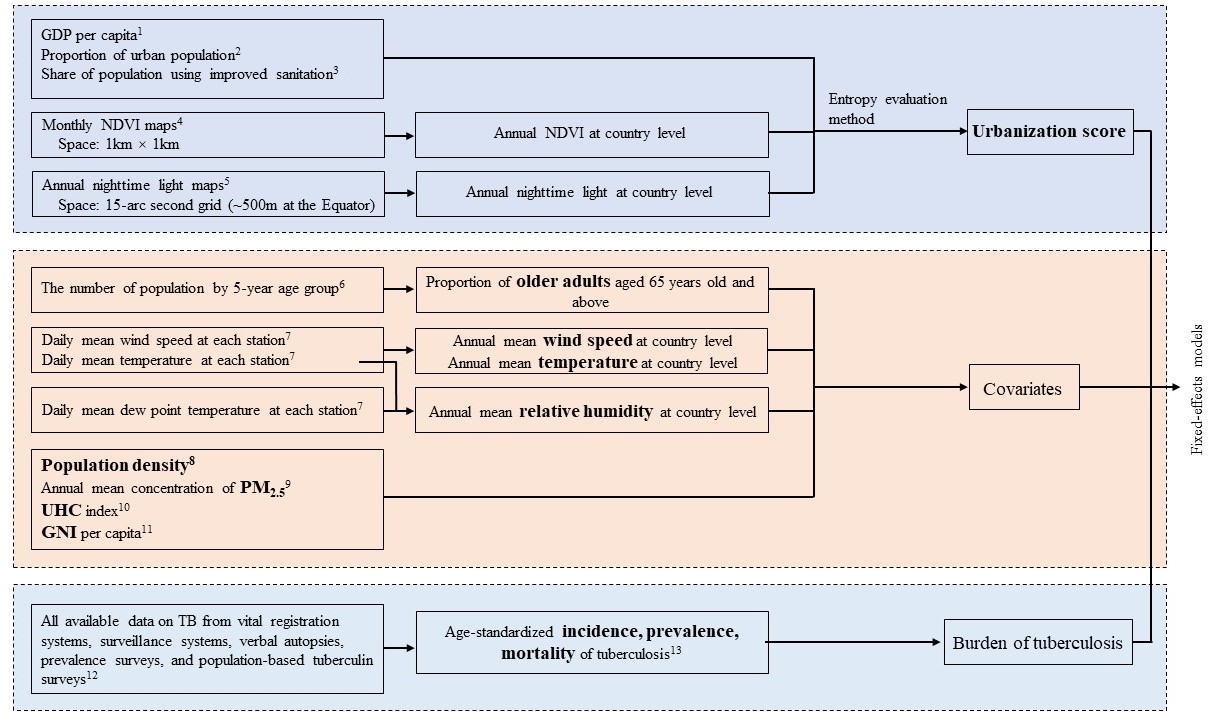


The bold text presents the variables inputted in the final fixed-effects models.

**Data sources**

1. World Bank. GDP per capita (current US$). 2022. https://data.worldbank.org/indicator/NY.GDP.PCAP.CD?view=chart.

2. World Bank. Urban population (% of total population). 2022. https://data.worldbank.org/indicator/SP.URB.TOTL.IN.ZS?view=chart.

3. WHO/UNICEF Joint Monitoring Programme (JMP) for Water Supply and Sanitation – processed by Our World in Data. “Usage of improved sanitation facilities” [dataset]. WHO/UNICEF Joint Monitoring Programme (JMP) for Water Supply and Sanitation [original data]. 2022. https://ourworldindata.org/explorers/water-and-sanitation?facet=none&Resource=Sanitation&Level+of+Use%2FAccess=Improved&Residence=Total&Relative+to+population=Share+of+population&country=IND~USA~KEN~OWID_WRL~BGD~ZAF~CHN.

4. Didan, K. MODIS/Terra Vegetation Indices 16-Day L3 Global 1km SIN Grid V061 [Data set]. NASA EOSDIS Land Processes Distributed Active Archive Center. 2021. https://doi.org/10.5067/MODIS/MOD13A2.061.

5. Earth Observation Group. Annual VNL V2. 2021. https://eogdata.mines.edu/nighttime_light/annual/v20/.

6. United Nations, Department of Economic and Social Affairs, Population Division. World Population Prospects 2022. 2022. https://population.un.org/wpp/Download/Standard/Population/.

7. National Centers for Environmental Information. Index of /data/global-summary-of-the-day. 2024. https://www.ncei.noaa.gov/data/global-summary-of-the-day/archive/.

8. World Bank. Population density (people per sq. km of land area). 2022. https://data.worldbank.org/indicator/EN.POP.DNST.

9. World Health Organization. SDG Indicator 11.6.2 Concentrations of fine particulate matter (PM2.5). 2022. https://www.who.int/data/gho/data/indicators/indicator-details/GHO/concentrations-of-fine-particulate-matter-(pm2-5).

10. World Health Organization. The Global Health Observatory: UHC Service Coverage Index (SDG 3.8.1). 2023. https://www.who.int/data/gho/data/indicators/indicator-details/GHO/uhc-index-of-service-coverage.

11. World Bank. GNI per capita, Atlas method (current US$). 2024. https://data.worldbank.org/indicator/NY.GNP.PCAP.CD?view=chart.

12. Global Burden of Disease Collaborative Network. Global Health Data Exchange: Global Burden of Disease Study 2019 (GBD 2019) Data Input Sources Tool. https://ghdx.healthdata.org/gbd-2019/data-input-sources?components=4&causes=297&locations=1.

13. Global Burden of Disease Collaborative Network. Global Burden of Disease Study 2019 (GBD 2019) Results. Seattle, United States: Institute for Health Metrics and Evaluation (IHME), 2020. https://vizhub.healthdata.org/gbd-results/.

# Figure S2: Estimated annual percentage change (EAPC, %) for incidence, prevalence, and mortality of total tuberculosis from 2012 to 2019


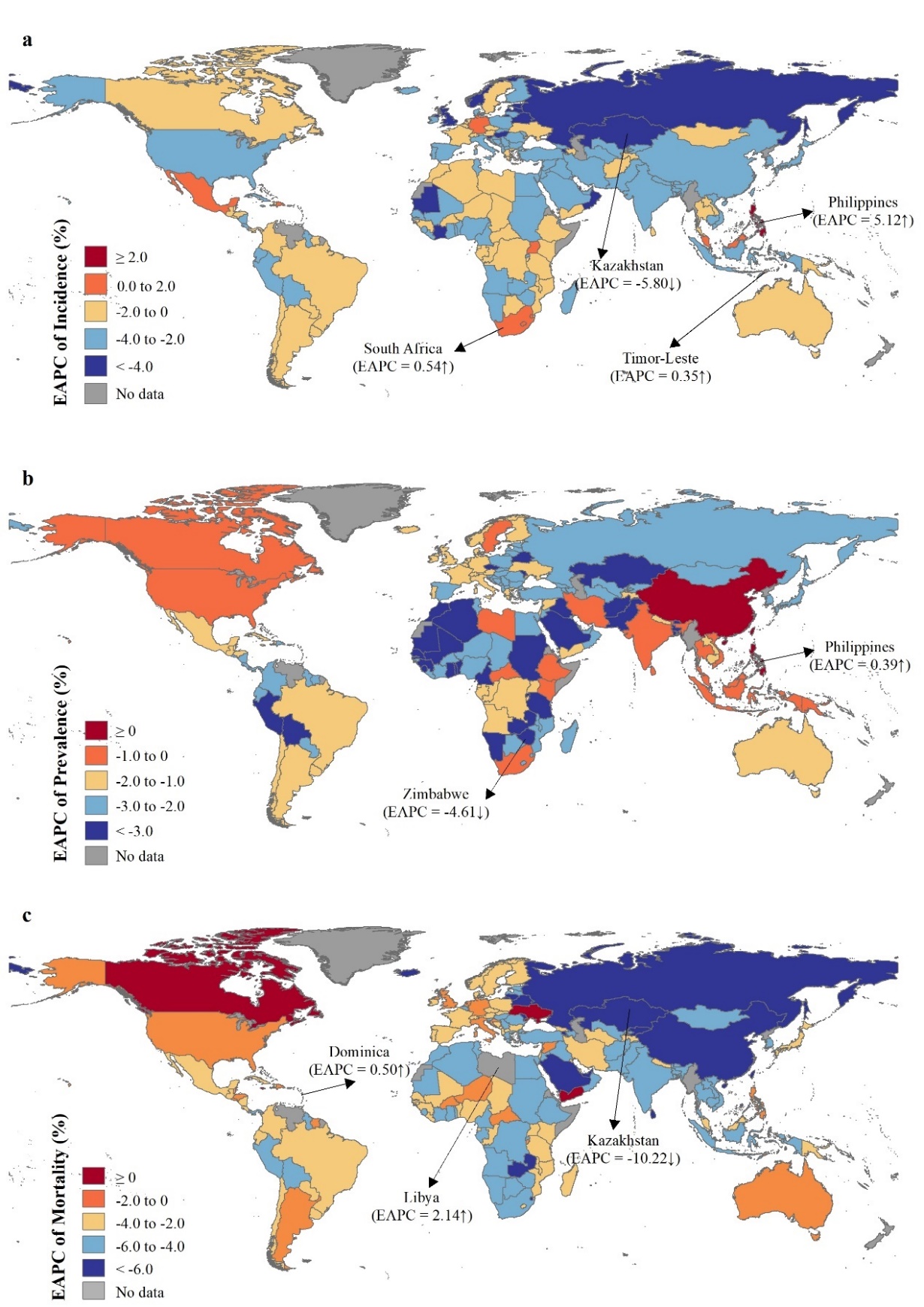


(a) estimated annual percentage change (%) for incidence of total tuberculosis; (b) estimated annual percentage change (%) for prevalence of total tuberculosis; (c) estimated annual percentage change (%) for mortality of total tuberculosis

# Figure S3: Estimated annual percentage change (EAPC, %) for burden of drug-susceptible tuberculosis from 2012 to 2019


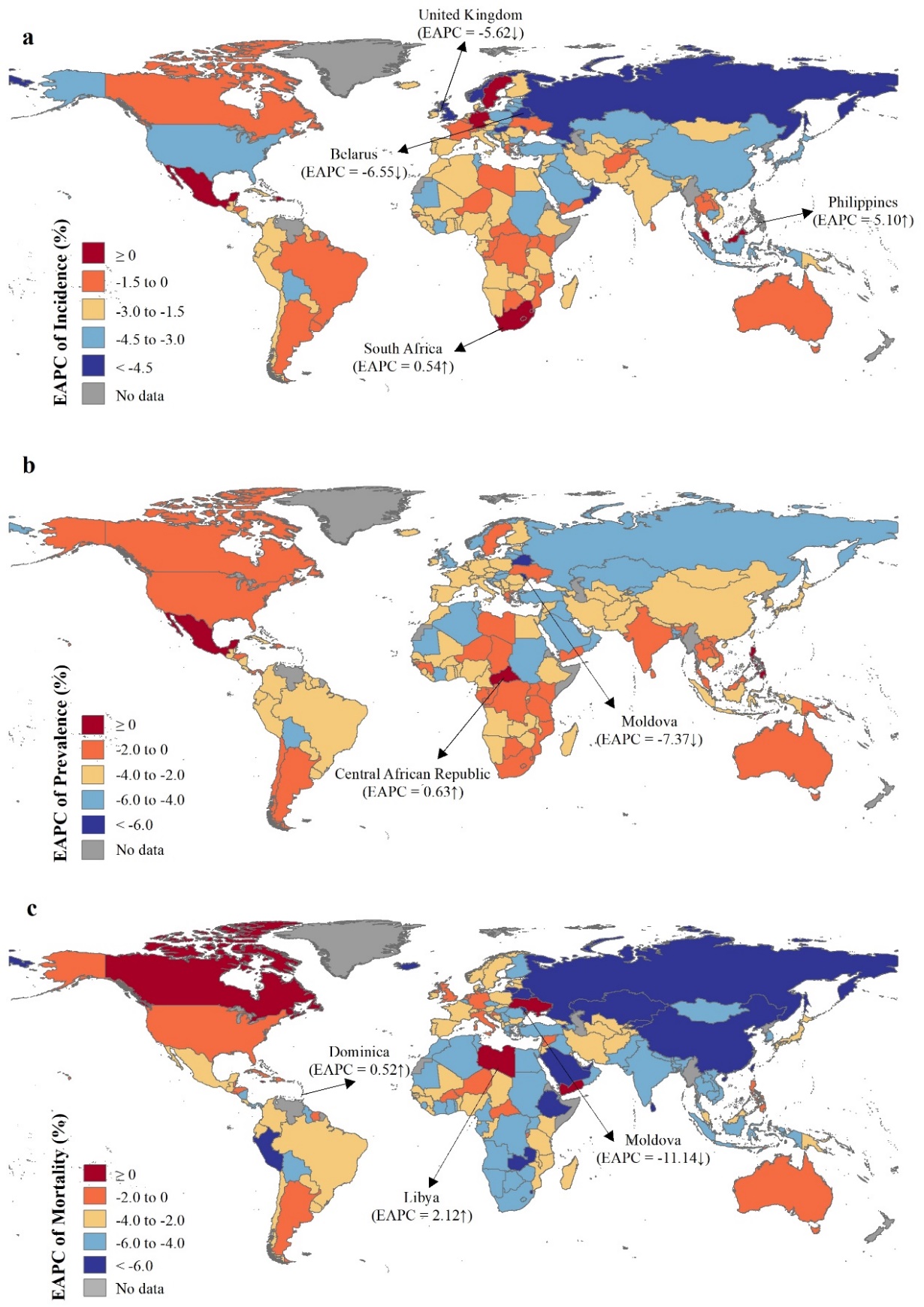


(a) estimated annual percentage change (%) for incidence of drug-susceptible tuberculosis; (b) estimated annual percentage change (%) for prevalence of drug-susceptible tuberculosis; (c) estimated annual percentage change (%) for mortality of drug-susceptible tuberculosis.

# Figure S4: Estimated annual percentage change (EAPC, %) for burden of multidrug-resistant tuberculosis from 2012 to 2019


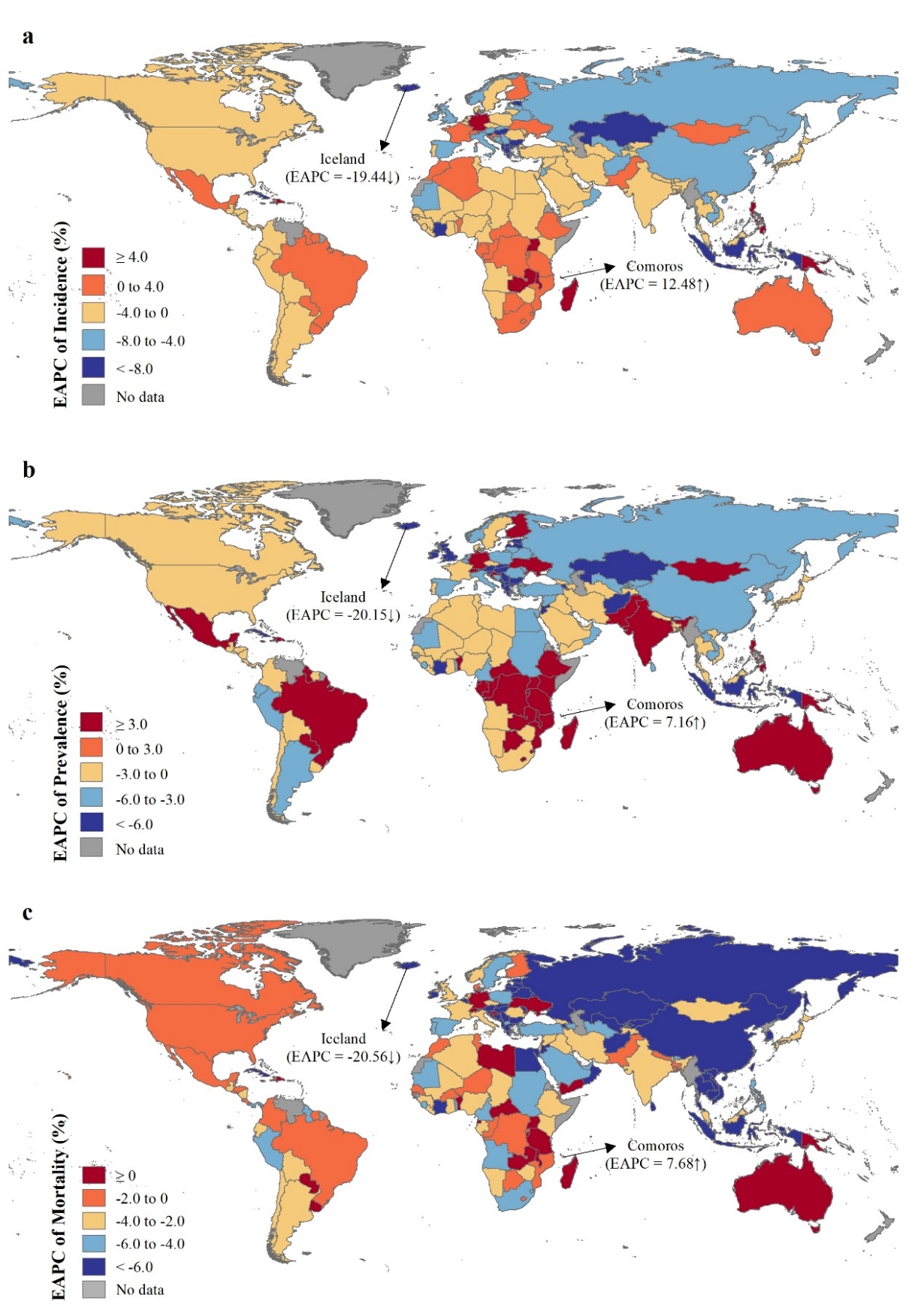


(a) estimated annual percentage change (%) for incidence of multidrug-resistant tuberculosis; (b) estimated annual percentage change (%) for prevalence of multidrug-resistant tuberculosis; (c) estimated annual percentage change (%) for mortality of multidrug-resistant tuberculosis.

# Figure S5: Estimated annual percentage change (EAPC, %) for incidence, prevalence, and mortality of extensively drug-resistant tuberculosis from 2012 to 2019


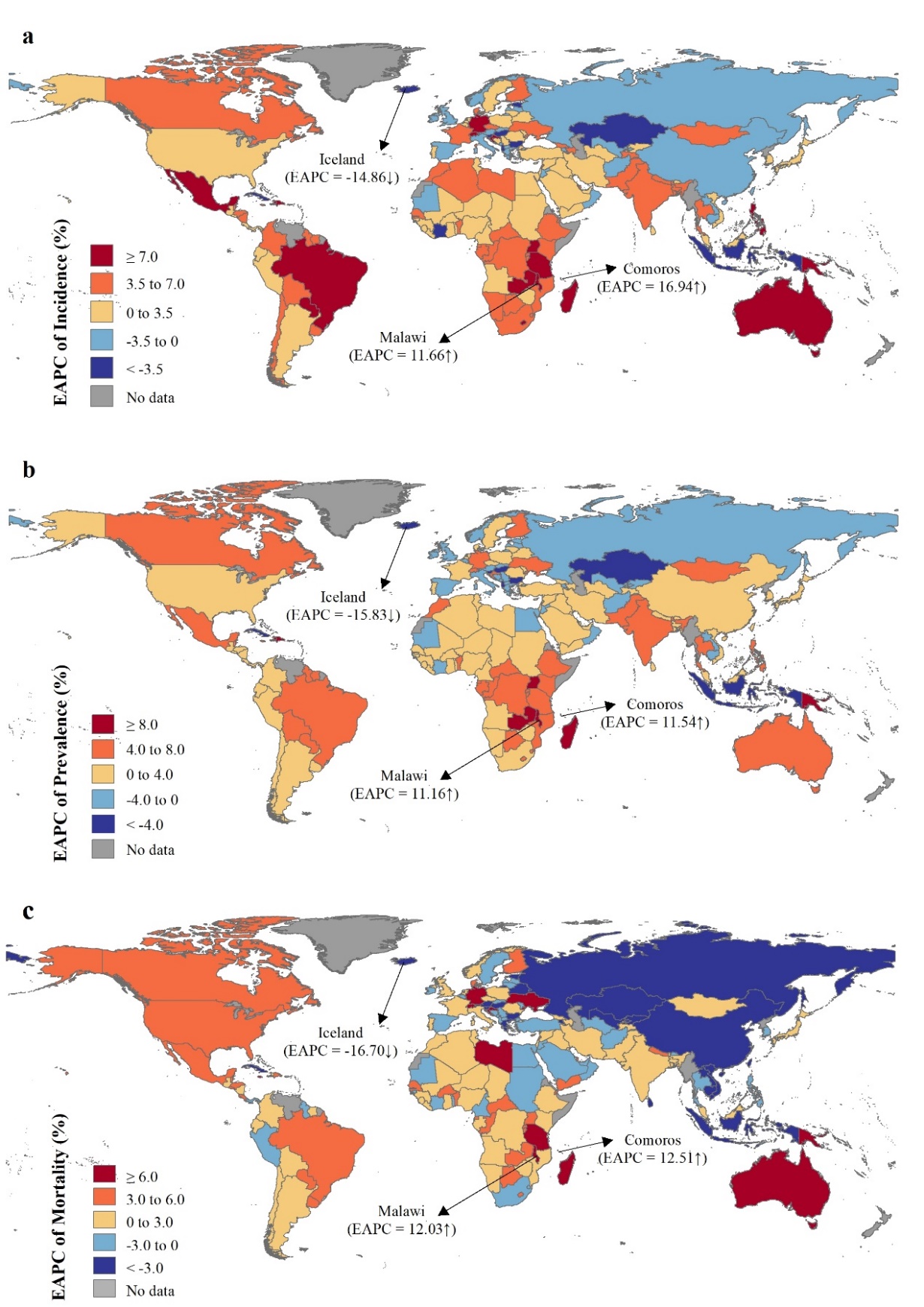


(a) estimated annual percentage change (%) for incidence of extensively drug-resistant tuberculosis; (b) estimated annual percentage change (%) for prevalence of extensively drug-resistant tuberculosis; (c) estimated annual percentage change (%) for mortality of extensively drug-resistant tuberculosis

# Figure S6: Association between single indicators of urbanization and the burden of tuberculosis


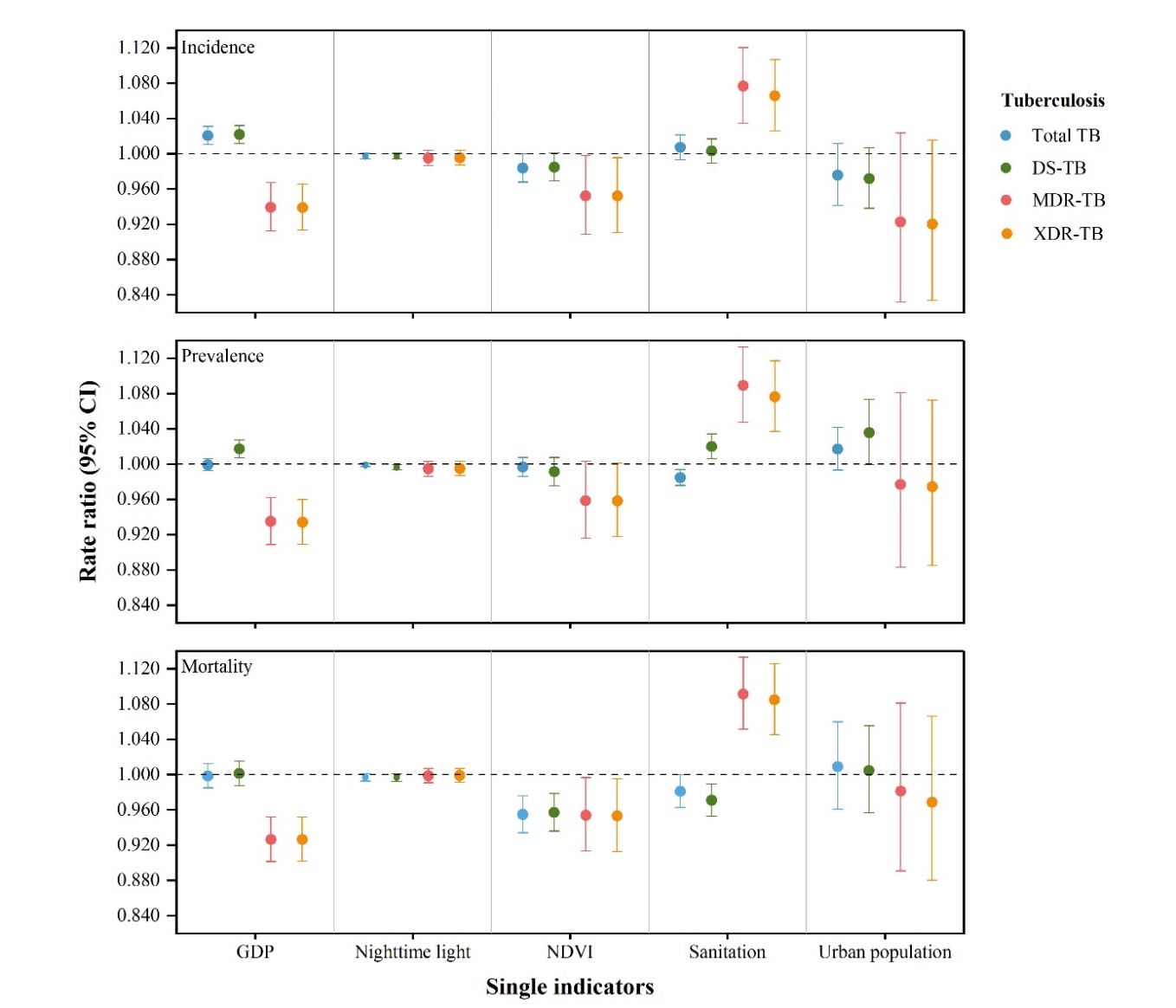


CI: confidence interval; GDP: gross domestic product (per capita); NDVI: normalized difference vegetation index. Sanitation indicated the proportion of population using improved sanitation. Urban population indicates the proportion of people living in urban areas.
